# Supplementary material for: Apoptosis, G1 Phase Stall, and Premature Differentiation Account for Low Chimeric Competence of Human and Rhesus Monkey Naive Pluripotent Stem Cells
Source: Stem Cell Reports. 2020 Dec 30;16(1):56–74. doi: 10.1016/j.stemcr.2020.12.004 (PMC7815945; doi:10.1016/j.stemcr.2020.12.004)
Supplement: Document S1. Supplemental Experimental Procedures, Figures S1–S7, and Table S1 [file mmc1.pdf]

Stem Cell Reports, Volume *16*

Supplemental Information

Apoptosis, G1 Phase Stall, and Premature Differentiation Account for Low Chimeric Competence of Human and Rhesus Monkey Naive Pluripotent Stem Cells

Irène Aksoy, Cloé Rognard, Anaïs Moulin, Guillaume Marcy, Etienne Masfaraud, Florence Wianny, Véronique Cortay, Angèle Bellemin-Ménard, Nathalie Doerflinger, Manon Dirheimer, Chloé Mayère, Pierre-Yves Bourillot, Cian Lynch, Olivier Raineteau, Thierry Joly, Colette Dehay, Manuel Serrano, Marielle Afanassieff, and Pierre Savatier

Stem Cell Reports, Volume *16*

Supplemental Information

Apoptosis, G1 Phase Stall, and Premature Differentiation Account for Low Chimeric Competence of Human and Rhesus Monkey Naive Pluripotent Stem Cells

Irène Aksoy, Cloé Rognard, Anaïs Moulin, Guillaume Marcy, Etienne Masfaraud, Florence Wianny, Véronique Cortay, Angèle Bellemin-Ménard, Nathalie Doerflinger, Manon Dirheimer, Chloé Mayère, Pierre-Yves Bourillot, Cian Lynch, Olivier Raineteau, Thierry Joly, Colette Dehay, Manuel Serrano, Marielle Afanassieff, and Pierre Savatier

Inventory of Supplemental Information

Figure S1

Figure S2

Figure S3

Figure S4

Figure S5

Figure S6

Figure S7

Table S1

Supplemental Experimental Procedures

Summary of media composition

List of antibodies

Supplemental References

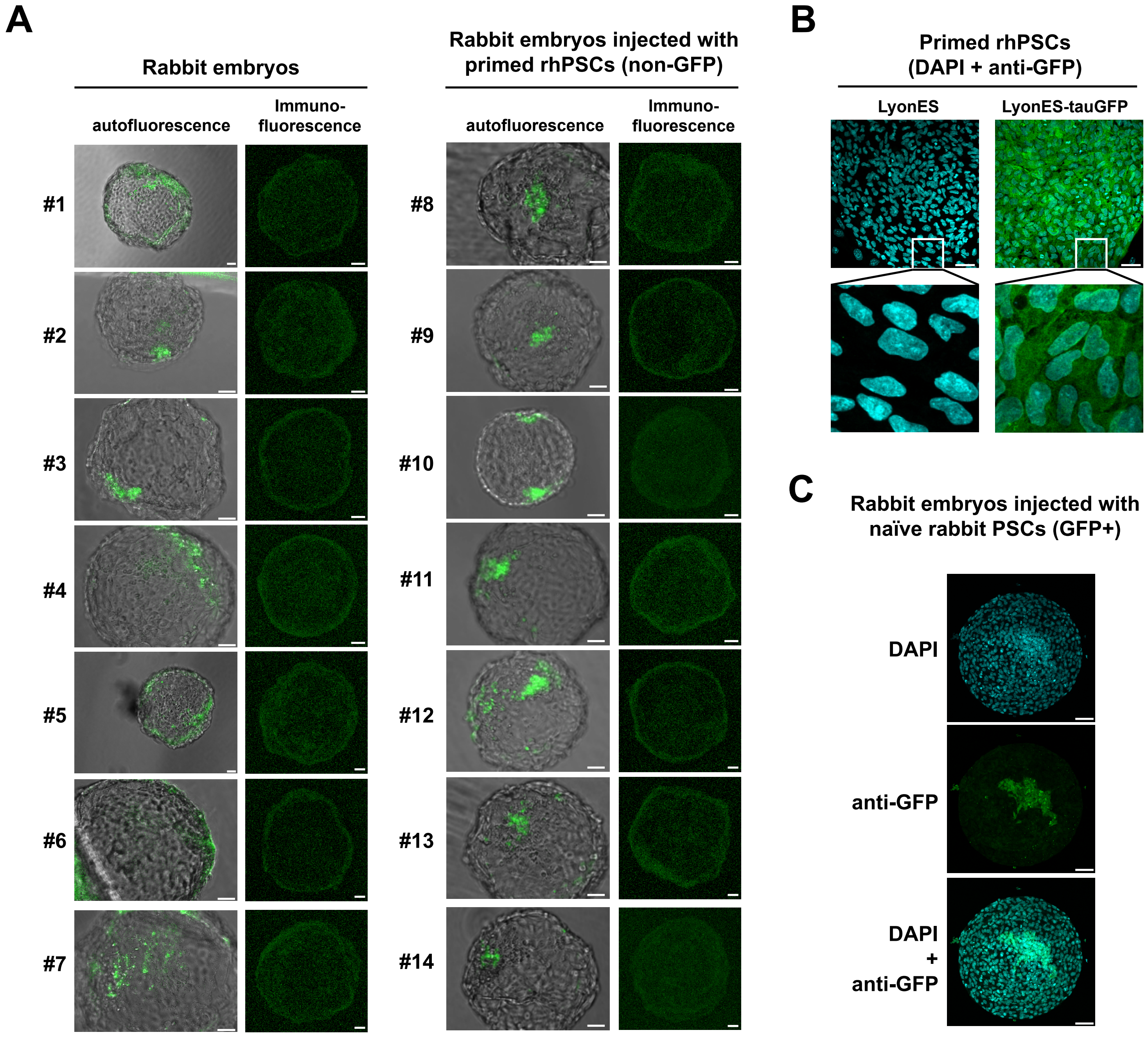

Figure S1: Evaluation of autofluorescence in rabbit embryos. Related to Figure 1. (A) Epifluoresence imaging and immunostaining of GFP in uninjected rabbit embryos (left panel) and in rabbit embryos injected with primed rhesus PSCs not expressing GFP (n = 40). **(B)** Immunostaining of GFP in primed LyonES-tGFP-(S3) rhesus PSCs. **(C)** Immunostaining of GFP in a late blastocyst-stage (E5) rabbit embryo after microinjection of rabbit naïve PSCs into morula-stage (E2) embryos (confocal imaging; scale bars: 50 μm).

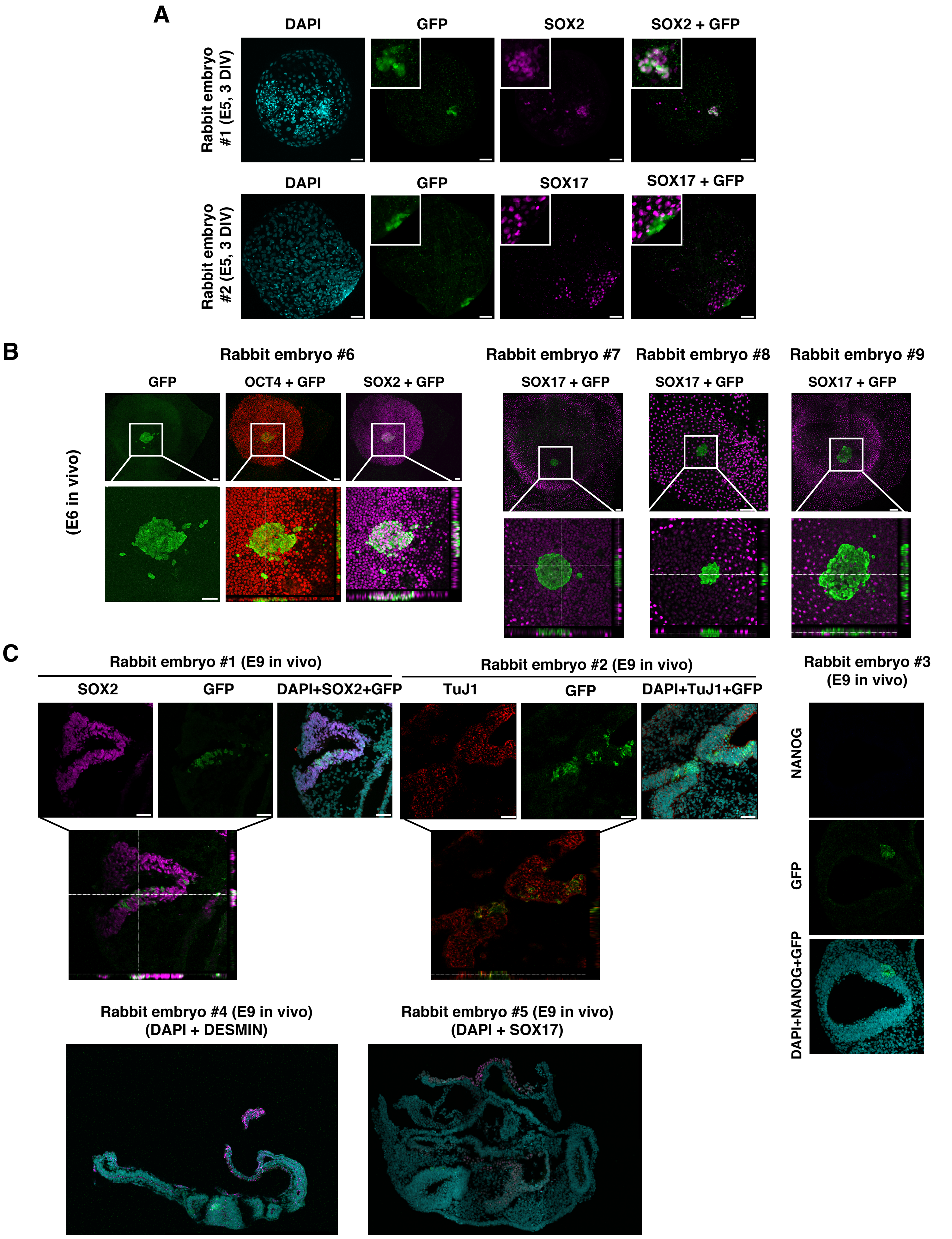

Figure S2: Colonization of rabbit embryos by mouse ESCs. Related to Figure 1. (A) Immunostaining of GFP, SOX2 and SOX17 in rabbit embryos at the late blastocyst-stage (E5) after microinjection of mESC-GFP-2i/LIF cells into morula-stage embryos (confocal imaging; scale bars: 50 μm) (n = 29). **(B)** Immunostaining of GFP, OCT4, SOX2 and SOX17 performed on rabbit embryos at the pre-gastrula stage (E6) after microinjection of mESC-GFP cells into morula-stage (E2) embryos and transfer to a surrogate mother. Bottom panels represent orthogonal sections. (confocal imaging; scale bars: 50 μm). **(C)** Immunostaining of GFP, SOX2, TuJ1, NANOG, DESMIN, and SOX17, in rabbit embryos at the post-implantation stage (E9) after microinjection of mESC-GFP cells into morula-stage embryos (confocal imaging; scale bars: 50 μm).

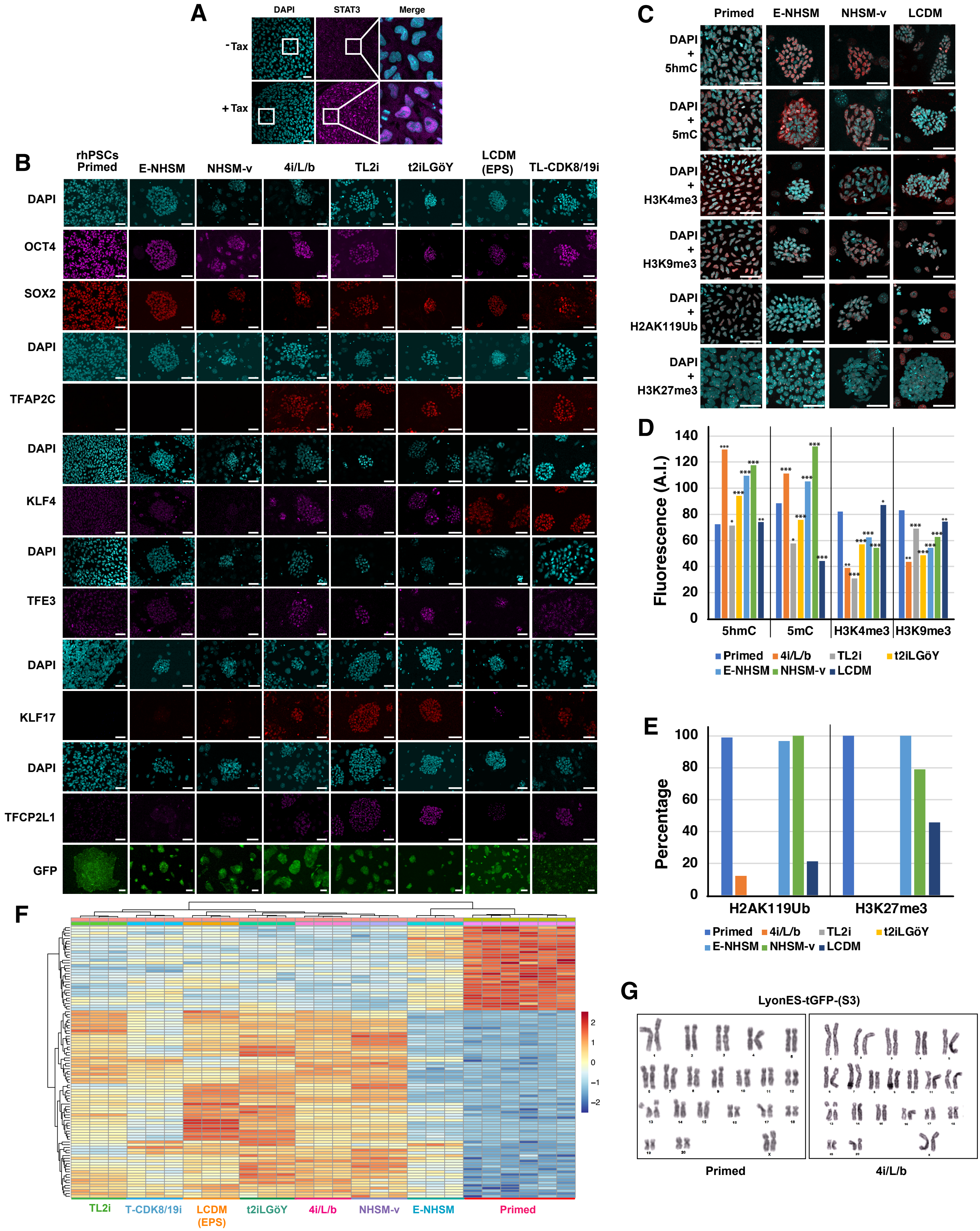

Figure S3: Characterization of LyonES-tGFP-(S3) cells. Related to Figure 2. **(A)** Immunolabeling of STAT3 in LyonES-tGFP-(S3) cells, before and after treatment with 250nM tamoxifen for 48 h (confocal imaging; scale bars: 50 μm). **(B)** **(C)** Immunolabeling of pluripotency markers and methylation marks in LyonES-tGFP-(S3) cells, before and after applying culture protocols for naïve conversion (confocal imaging; scale bars: 50 μm). **(D)** Histogram representing the quantification of 5hmC, 5mC, H3K4me3 and H3K9me3 methylation marks (Student's t test, p-value ≤ 0,05 : *; p-value ≤ 0,01 : **; p < 0,001: ***). **(E)** Histogram representing the percentage of H2AK119Ub and H3K27me3 marks. **(F)** Heatmap of transcriptome data (mean values/cell category most differentially expressed 100 genes, listed in Table S2) using Pearson correlation coefficient as a measure of Euclidian distance between rows and between columns. (G) G-banding karyotype of LyonES-tGFP-(S3) primed cells (left panel) and a batch of LyonES-tGFP-(S3) naïve 4i/L/b cells.

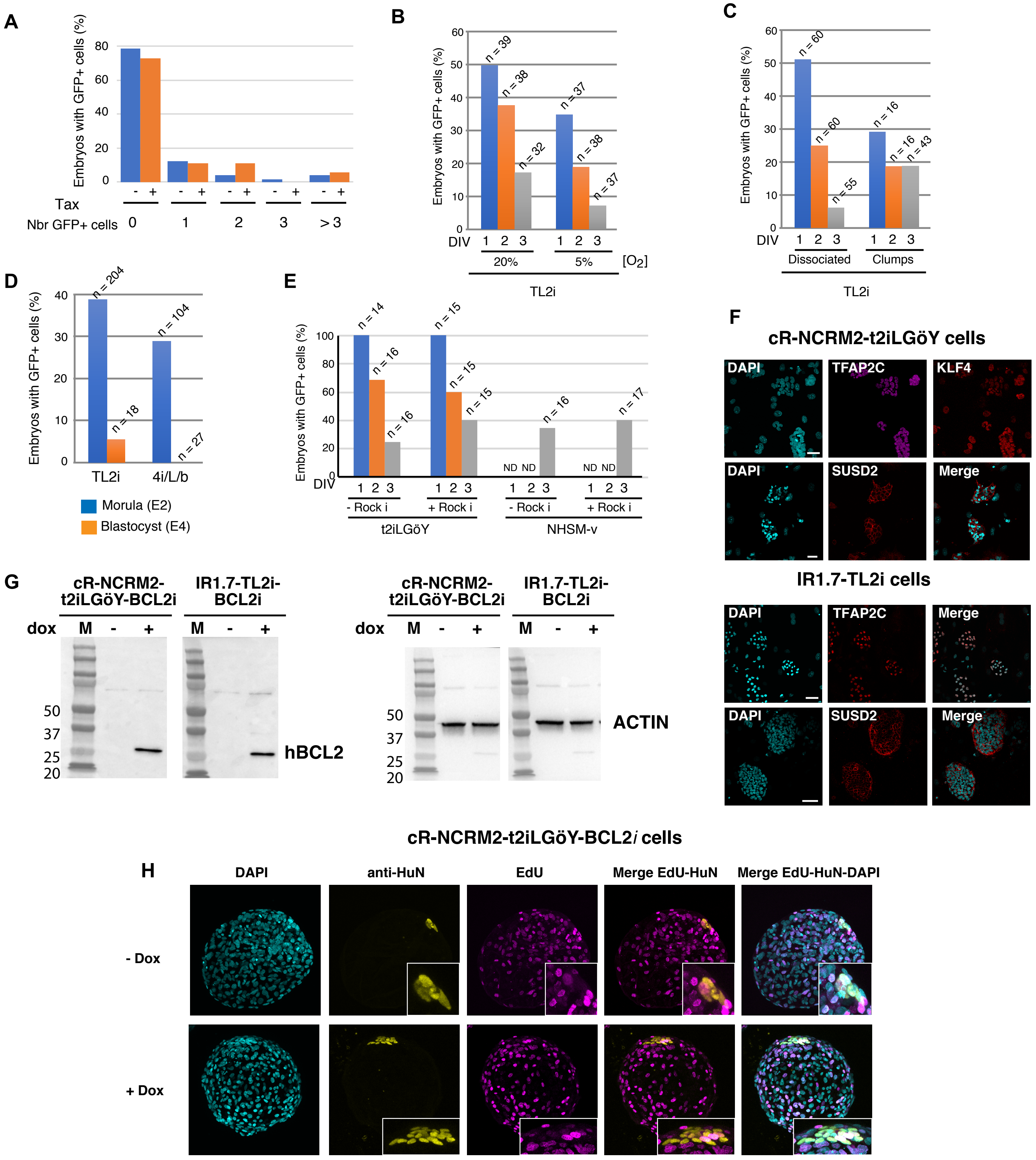

Figure S4: Optimization of the conditions for microinjection and Colonization of rabbit embryos by human PSCs. Related to Figures 3 and 4. (A) Histogram of percentage of rabbit embryos with GFP+ cells after microinjection of rhesus TL2i cells at the morula-stage (E2) and in vitro culture for 3 days in gradual medium in the presence (+) or in the absence (-) of 250nM tamoxifen. **(B)** Histogram of percentage of rabbit embryos with GFP+ cells after microinjection of rhesus TL2i cells at the morula-stage (E2) under normoxic (20%) and hypoxic (5%) conditions. Analyses were performed at 1, 2, and 3 DIV (n = 221). **(C)** Histogram of the percentage of rabbit embryos with GFP+ cells after microinjection of dissociated or clumps of rhesus TL2i cells into morula-stage (E2) embryos. Analyses were performed at 1, 2, and 3 DIV (n = 250). **(D)** Histogram of the percentage of rabbit embryos with GFP+ cells after microinjection of rhesus 4i/L/b and TL2i cells into morula-stage (E2) or blastocyst-stage (E4) embryos. Analyses were performed at 3 DIV (n = 353). **(E)** Histogram of the percentage of rabbit embryos with GFP+ cells after microinjection of rhesus t2iLGöY and E-NHSM cells into morula-stage (E2) embryos, in the presence or absence of ROCK inhibitor (n = 124). Analyses were performed at 3 DIV. **(F)** Immunostaining of TFAP2C and SUSD2 in NCRM2-t2iLGöY and IR1.7 TL2i cells (confocal imaging; scale bars: 50 μm). **(G)** Western blots of hBCL2 in cR-NCRM2- t2iLGöY and IR1.7-TL2i cells before (-) and after (+) treatment with 1 μg/mL doxycycline for 72 hours. **(H)** Immunostaining of HuN anf EdU in late blastocyst-stage rabbit embryos (E5, 3 DIV) after microinjection of human cR-NCRM2-t2iLGöY-BCL2*i* PSCs before (-Dox) and after (+Dox) treatment with 1 μg/mL doxycycline from 24 hours before injection to 72 hours after injection.

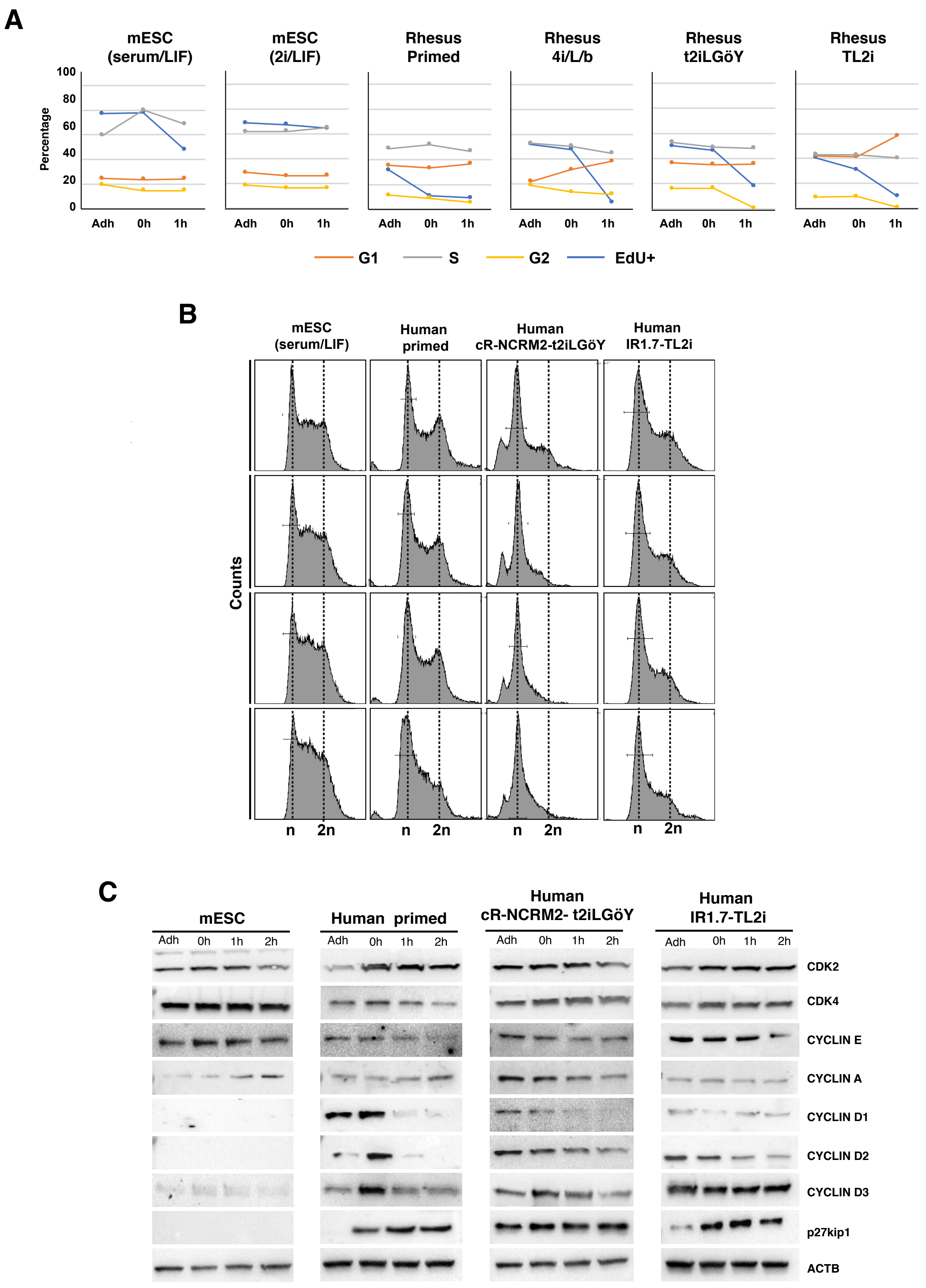

Figure S5: Cell-cycle parameters of mouse ESCs, rhesus PSCs, and human iPSCs, before and after reprogramming to naïve states. Related to Figure 6. (A) Histograms showing the cell cycle distribution of mESCs (serum/LIF and 2i/LIF) and rhesus PSCs (primed, 4i/L/b, t2iLGöY and TL2i) in Adh, 0h, 1h, and 2h conditions analyzed by flow cytometry after EdU incorporation and PI staining (% EdU+, G1, and S/G2 cells). **(B)** Cell cycle distribution of mESCs (serum/LIF) and human PSCs (primed, cR-NCRM2-t2iLGöY and IR7.1-TL2i) in Adh, 0h, 1h, and 2h conditions analysed by flow cytometry after PI staining. **(C)** Western blot analysis of cell-cycle regulators in mESCs (serum/LIF) and human iPSCs (primed, cR-NCRM2-t2iLGöY and IR7.1-TL2i) in Adh, 0h, 1h, and 2h conditions (results of 3 experiments).

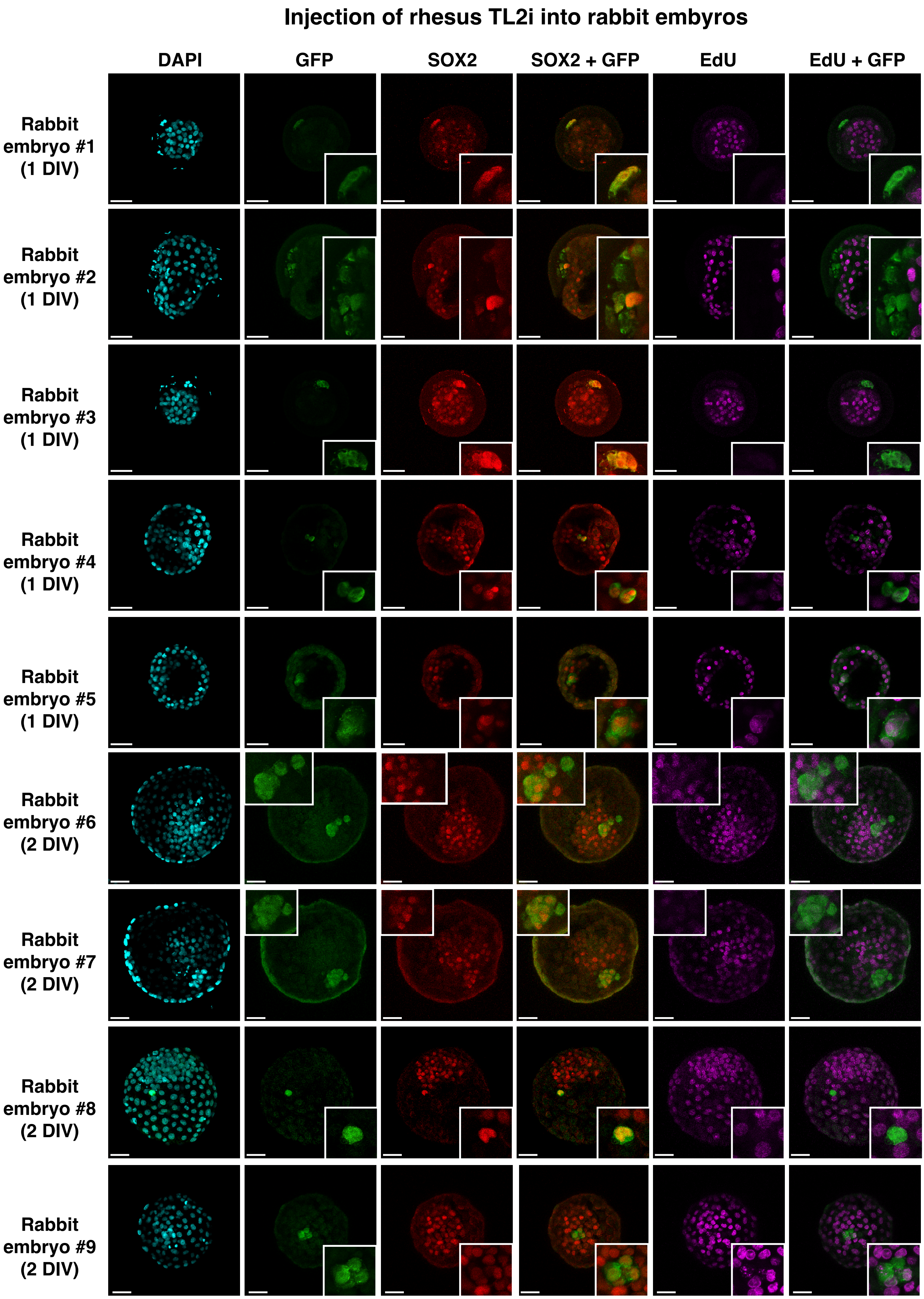

Figure S6: DNA replication of Rhesus PSCs after microinjection into rabbit embryos. Related to Figure 7. Immunostaining of GFP, Sox2, and EdU in late blastocyst-stage rabbit embryos (E5, 3 DIV) after microinjection of rhesus TL2i cells into morula-stage (E2) embryos (confocal imaging; scale bars: 50 μm).

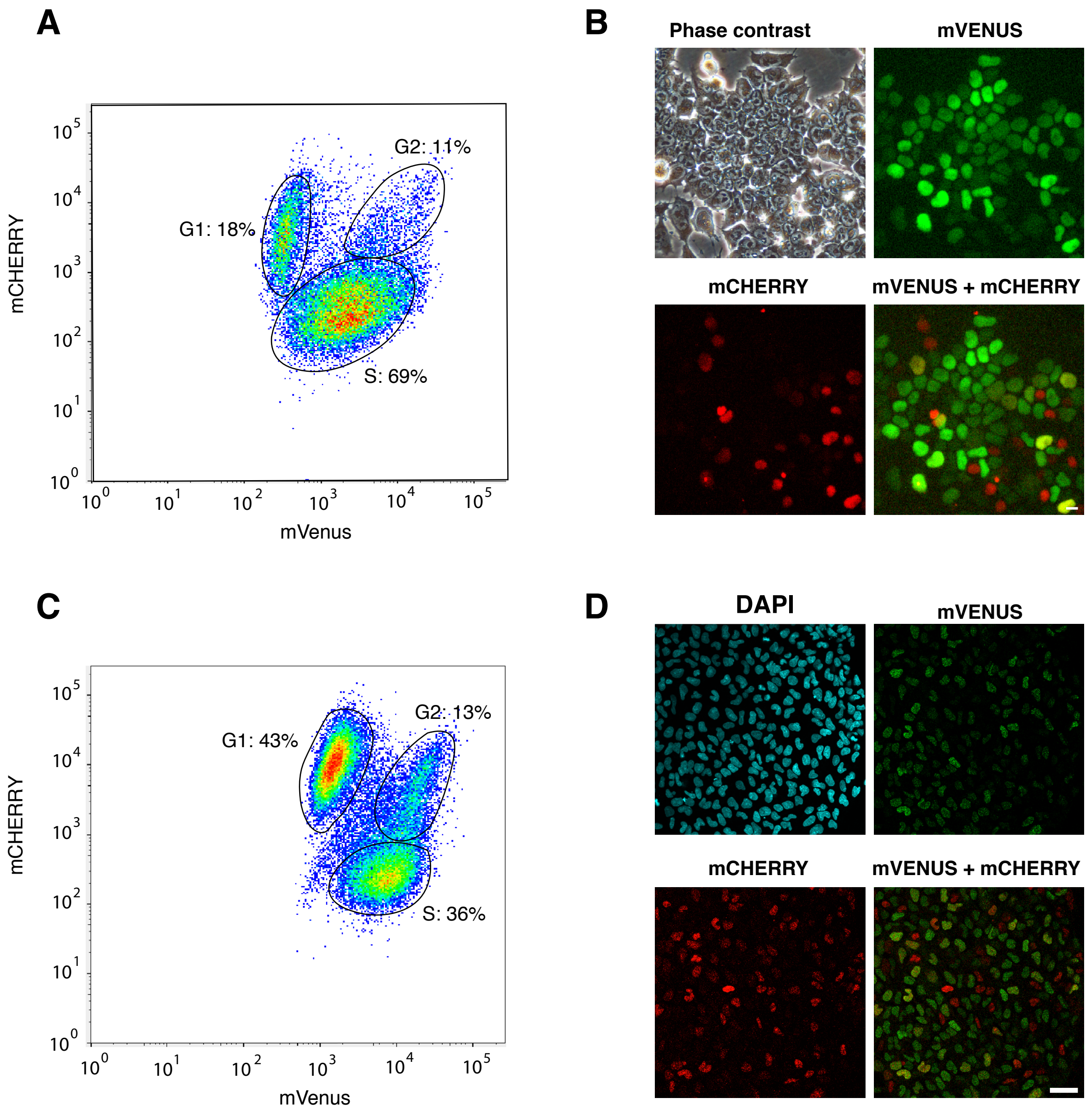

Figure S7: Characterization of mouse ESCs and rhesus PSCs harboring the FUCCI(CA) cell cycle reporter. Related to Figure 7. (A) Flow cytometry analysis and **(B)** epifluorescence imaging of mESCs-FUCCI (CA) cells. **(C)** Flow cytometry analysis and **(D)** epifluorescence imaging of rhesus primed-FUCCI (CA) cells (scale bars: 50 μm).

Supplemental Experimental Procedures

Cell lines, media composition, and culture

mESC lines were routinely cultured in Glasgow's modified Eagle's medium (Gibco) supplemented with 10% fetal calf serum (CRC0406; PerbioScience) and 1,000 U/mL LIF on gelatin-coated dishes. They were also cultured in N2B27 supplemented with 1,000 U/mL LIF, 1 μM PD0325901, and 3 μM CHIR99021 (2i/LIF), as indicated. Both the LyonES-tGFP and Lyon-ES-tGFP-(S3) rhesus PSC lines (Wianny et al., 2008, Chen et al., 2015a) and the human iPSC line IR7.1 (Chen et al., 2015a), (Ng et al., 2012) cell lines were routinely cultured at 37 °C in 5% CO_2 and 5% O_2 in knockout Dulbecco's modified Eagle's medium (KO-DMEM) supplemented with 20% knockout serum replacement (KOSR), 1 mM glutamine, 0.1 mM β-mercaptoethanol (Sigma), 1% non-essential amino acid (Gibco), and 4–8 ng/mL FGF2 (Gibco) on growth-inactivated murine embryonic fibroblasts.

Primed to naïve conversion was performed using previously described protocols, including E-NHSM (https://hannalabweb.weizmann.ac.il) (Gafni et al., 2013), NHSM-v (Chen et al., 2015b), 4i/L/b (Fang et al., 2014), 5iL/A (Theunissen et al., 2014), t2iLGöY (Guo et al., 2017), TL2i (Chen et al., 2015a), TL-CDK8/19i [modified from (Lynch et al., 2020)], and LCDM (EPS) (Yang et al., 2017a). Primed cells were dissociated and plated on fresh feeder cells for 24 h before shifting to the naïve culture media. Fresh medium was added daily to the culture plates. The medium composition for E-NHSM reprogramming (https://hannalabweb.weizmann.ac.il) (Gafni et al., 2013) was as follows: Neurobasal: DMEM-F12 (1:1) supplemented with N2B27 (Gibco), L-ascorbic acid, 0.5% KOSR, 10 ng/mL human LIF (Peprotech), 5 μM IWR1 (Sigma), 1.5 μM CHIR99021 (Miltenyi Biotec), 1 μM PD0325901 (Miltenyi Biotec), 2 μM BIRB796 (Axon), 5 μM SP600125 (Tocris), 2 μM Gö6983 (Tocris), 20 ng/mL activin A (Peprotech), and 1 μM CGP77675 (Axon). Cells were passaged every 3–4 days by single-cell dissociation using Accutase (Gibco). ROCK inhibitor (Y-27632; Miltenyi Biotec) was added during passaging for 24 h. The NHSM-v protocol is a modified version of the NHSM protocol applied to cynomolgus macaque ESCs (Chen et al., 2015b). These cells were cultured in KO-DMEM supplemented with 20% KOSR, 8 ng/mL FGF2 (Gibco), 10 ng/mL hLIF (Peprotech), 3 μM CHIR99021 (Miltenyi Biotec), 1 μM PD0325901 (Miltenyi Biotec), 10μM SP600125 (Tocris), and 10 μM SB203580 (Tocris). Cells were passaged every 3–4 days by single-cell dissociation using 1 mg/mL Accutase (Gibco). ROCK inhibitor (Y-27632; Miltenyi Biotec) was added during passaging for 24 h. The 4i/L/b media was adapted from the protocol published by Fang *et al.* (Fang et al., 2014). Cells were cultured in KO-DMEM supplemented with 20% KOSR (Gibco), 0.5 μM PD0325901 (Miltenyi Biotec), 3 μM CHIR99021 (Miltenyi Biotec), 10 μM SP600125 (Tocris), 10 μM SB203580 (Tocris), 10 ng/mL hLIF (Peprotech), and 2.5 ng/mL FGF2 (Gibco). Cells were passaged every 3–4 days by single-cell dissociation using 0.05% Trypsin-ethylenediaminetetraacetic acid (Trypsin-EDTA; Gibco). ROCK inhibitor (Y-27632; Miltenyi Biotec) was added during passaging for 24 h. Primed cells were converted to 5i/L/A state using the following medium composition (Theunissen et al., 2014): DMEM-F12 (1:1) medium supplemented with N2 (Gibco), B27 (Gibco), 0.5% KOSR (Gibco), 1 μM PD0325901 (Miltenyi Biotec), 1 μM IM-12 (Enzo), 0.5 μM SB590885 (Tocris), 1 μM WH-4-023 (Tocris), and 20 ng/mL activin A (Peprotech). ROCK inhibitor (Y-27632; Miltenyi Biotec) was added during passaging for 24 h. Primed cells were converted to the t2iLGöY state using the following medium composition (Guo et al., 2017): first in cRM1 media for three days with 1 μM PD0325901 (Miltenyi Biotec), 10 ng/mL hLIF (Peprotech) and 1mM Valproic acid (VPA), followed by two days in cRM2 media with DMEM-F12 (1:1) medium supplemented with N2, B27 (Gibco), 1 μM PD0325901 (Miltenyi Biotec) and 250 μM Vitamin C (Sigma-Aldrich) and then the final media containing DMEM-F12 (1:1) medium supplemented with N2, B27 (Gibco), 1 μM PD0325901 (Miltenyi Biotec), 1,000 U/mL LIF, 1 μM Gö6983 (Bio-techne), 2 μM XAV939 (Sigma). Cells were passaged every 4–5 days by single-cell dissociation using Tryple (Gibco). ROCK inhibitor (Y-27632; Miltenyi Biotec) was added during passaging for 24 h. For reprogramming to the TL2i state, PSCs expressing the inducible fusion protein STAT3-ER^{T2} were cultivated in the TL2i medium composed of KO-DMEM (Gibco), 20% KOSR (Gibco), 10,000 U/mL LIF, 3 μM CHIR99021 (Miltenyi Biotec), 1 μM PD0325901 (Miltenyi Biotec), and 250 nM 4′-Hydroxy-Tamoxifen (4′OHT; Calbiochem). Cells were passaged every 3–4 days by single-cell dissociation using 0.05% Trypsin-EDTA (Gibco). ROCK inhibitor (Y-27632; Miltenyi Biotec) was added during passaging for 24 h. For TL-CDK8/19i protocol, cells were cultured in medium composed of KO-DMEM (Gibco), 20% KOSR (Gibco), 10,000 U/mL LIF, and 1 μM CNIO-47799. Cells were passaged every 3–4 days by single-cell dissociation using 0.05% Trypsin-EDTA (Gibco). ROCK inhibitor (Y-27632; Miltenyi Biotec) was added during passaging for 24 h.Primed cells were converted to the LCDM (EPS) (Yang et al., 2017) state using medium with the following composition: DMEM-F12 (1:1), N2 (Gibco), B27 (Gibco), 5% KOSR (Gibco), 10 ng/mL hLIF (Peprotech), 1 μM CHIR99021 (Miltenyi Biotec), 2 μM (S)-(+)-Dimethindene maleate (Tocris), 2 μM minocycline hydrochloride (Santa Cruz Biotechnology), and 0.75 μM IWR1 (Sellekchem). Cells were passaged every 3–4 days by single-cell dissociation using 0.05% Trypsin-EDTA (Gibco). ROCK inhibitor (Y-27632; Miltenyi Biotec) was added during passaging for 24 h. All media compositions are summarized below.

To avoid the accumulation of karyotypic abnormalities, new naïve cell batches were regularly produced and none of the naïve cells were cultured for more than 2 months after reprogramming.

G-banding karyotyping

G-banding karyotyping was performed by Cell Guidance Systems (www.cellgs.com).

Generation of FUCCI reporter rhesus PSCs and mESCs

The *PB-Puro^R-CAG-mVenus : hGeminin-IRES-mCherry : hCdt1* plasmid was generated as follows. First, an EcoRI/XbaI DNA fragment from the *mCherry-hCdt1(1/100)Cy(-)/pcDNA3* plasmid (Sakaue-Sawano et al., 2017) containing the mCherry : hCdt1 cassette was sub-cloned between the EcoRI and XbaI restriction sites in *PB-CAG-cHA-IRES-hyg* to generate *PB-Hygro-mcherry : hCdt1*. Second, a AlfII/XbaI fragment from the *mVenus-hGeminin(1/110)/pcDNA3* plasmid (Sakaue-Sawano et al., 2017) containing the mVenus : hGeminin cassette was subcloned between the SapI and AgeI restriction sites in *PB-Hygro-mcherry : hCdt1* to generate *PB-Hygro-mcherry : hCdt1-mVenus : hGeminin*. Third, a SapI/AgeI fragment containing the *Puro^R* gene and the CAG promoter was sub-cloned between the SapI and AgeI restriction sites in *PB-Hygro-mcherry : hCdt1-mVenus : hGeminin* to generate the *PB-Puro^R-CAG-mVenus : hGeminin-IRES-mCherry : hCdt1* plasmid. Rhesus PSCs and mESCs were transfected using the NEON transfection system according to the instructions provided by the manufacturer. Briefly, cells were dissociated and resuspended at a density of 10.10^6 cells/mL. For transfection, 100 µL of the cell suspension was mixed with 2.5 µg of the *PB-Puro^R-CAG-mVenus : hGeminin-IRES-mCherry : hCdt1* vector and 2.5 µg of *PBase* plasmid. Transfection parameters used for rhesus PSCs were 1,050 V, 20 ms, and 2 pulses. Cells were plated on growth-inactivated murine embryonic fibroblasts in medium supplemented with 10 µM ROCK inhibitor (Y-27632; Miltenyi Biotec) and selected in 250 ng/mL G418. For mESCs, the parameters used were 1,200 V, 20 ms, and 2 pulses. Cells were analyzed using a FACS LSR II (Becton-Dickinson) equipped with 355, 488, and 561 nm lasers. Data were acquired and analyzed using the DiVa software.

Generation of BCL2 inducible human PSCs

The *BCL2* plasmid was generated as follows. First, a DNA fragment from the *pCS2+Flag-BCL2* plasmid was amplified by PCR using primers flanked by *Kpn*I and *Spe*I restriction sites at the 5' and 3' end, respectively. The PCR fragment was then subcloned in pXlone-GFP-Neo3R-P2A-TRE-EF1 plasmid. This plasmid was obtained after modification of the *Xlone-GFP* plasmid (a gift from Xiaojun Lian, Addgene plasmid #96930) in which the Blasticidin resistance gene was replaced by Neomycin. cR-NCRM2-t2iLGöY and IR1.7-TL2i cells were transfected using the NEON transfection system according to the instructions provided by the manufacturer. Briefly, cells were dissociated and resuspended at a density of 10.10^6 cells/mL. For transfection, 100 µL of the cell suspension was mixed with 2.5 µg of the *pXlone-Flag-BCL2-Neo3R-P2A-TRE-EF1* vector and 2.5 µg of *PBase* plasmid. Transfection parameters used were 1,050 V, 20 ms, and 2 pulses. Cells were plated on growth-inactivated murine embryonic fibroblasts in medium supplemented with 10 µM ROCK inhibitor (Y-27632; Miltenyi Biotec) and selected in 250 ng/mL G418.

Generation of cynomolgus monkey embryos and cell microinjection

Macaque cynomolgus embryos were produced through ovarian stimulation, followed by intra-cytoplasmic sperm injection (ICSI) of oocytes, as previously described (Tachibana et al., 2012). Briefly, females received twice-daily injections of 25 IU of recombinant human follicle-stimulating hormone (MSD laboratories) from days 1 to 8 (starting at day 1–4 of the menstrual cycle). They received 30 IU of recombinant human luteinizing hormone (Merck) on days 7 and 8. Females received an injection of gonadotropin-releasing hormone antagonist (MSD laboratories) (0.075 mg/kg body weight) on day 8, and an injection of 1,040 IU of human chorionic gonadotropin (Merck) at day 8. Estradiol, luteinizing hormone, progesterone dosage, and ultrasonographic scans were performed to monitor ovarian response. Follicle aspiration and oocyte retrieval were performed by laparoscopy 36 h after injection of human chorionic gonadotropin. Oocytes were transferred to HEPES-buffered TALP medium containing 3 mg/mL of bovine serum albumin (Sigma). Cumulus and granulosa cells were removed by incubating the oocytes for 30 s with hyaluronidase (300 µg/mL). Metaphase II stage oocytes were selected for ICSI and transferred to drops of hamster embryo culture medium-9 (HECM9) covered by liquid paraffin (Origio) at 37 °C in a 5% O_2 + 5% CO_2 atmosphere. Four days after fertilization (E4), morula-stage embryos were microinjected with 10 cells and further cultured in drops of PSC medium for 4 h. PSC media were those used for culturing the cells prior to injection, depending on the reprogramming protocols. After 4 h, embryos were transferred into a 1:1 mix of PSC medium and HECM9 and further cultured for 20 h. After 24 h, embryos were transferred to HECM9 and further cultured.

Production of rabbit embryos, cell microinjection, embryo culture, and transfer

Rabbit embryos were produced by ovarian stimulation. Briefly, sexually mature New Zealand white rabbits were injected with follicle-stimulating hormone and gonadotropin-releasing hormone, followed by artificial insemination or breeding, as previously described. Eight-cell-stage embryos (E1.5) were flushed from

explanted oviducts 36–40 h after insemination and cultured in a 1:1:1 mixture of RPMI 1640 medium, DMEM, and Ham's F10 (RDH medium; Thermo Fisher Scientific) at 38 °C in 5% CO_2 until cell microinjection. For the latter procedure, 10 cells were microinjected under the zona pellucida of morula (E2.5)- or blastocyst (E4)-stage rabbit embryos. The embryos were further cultured using the same experimental procedure as for monkey embryo. For embryo transfer, surrogate mothers were prepared through intramuscular injection of 1.6 µg of buserelin acetate (Intervet). Morula-stage embryos (6–8) were transferred to each oviduct of the recipient by laparoscopy. Four days after transfer, pre-implantation embryos (E6) were recovered by flushing the explanted uterine horns. Post-implantation-stage embryos (E9) were recovered by dissecting the uterine horns.

Immunoblotting
For immunoblotting, cells were grown on Geltrex to avoid contamination by mouse embryonic fibroblasts. Frozen cell pellets were lysed in RIPA buffer complemented with protease and phosphatase inhibitors. Protein lysates were then cleared by centrifugation (14,000 r.p.m. for 30 min). After SDS–PAGE and electroblotting on polyvinylidene difluoride, the membranes were incubated with specific primary antibodies (antibodies used in this study are listed below). Blots were incubated with horseradish peroxidase-coupled anti-mouse or -rabbit immunoglobulin G (Jackson ImmunoResearch) and developed with Clarity Western ECL Substrate (BIO-RAD).

Histology, histochemistry, immunostaining
Monkey (E7) and rabbit (E3, E4, E5, and E6) pre-implantation embryos were fixed in 2% paraformaldehyde (PFA) for 20 min at room temperature. After three washes in phosphate-buffered saline (PBS) containing 0.1% Tween-20 (PBS-0.1%T), embryos were permeabilized in PBS-1%T overnight at 4 °C on a rotating shaker. Embryos were subsequently placed in blocking solution (PBS-0.1%T) supplemented with 5% donkey serum for 1 h at room temperature. There were incubated with primary antibodies diluted in blocking solution overnight at 4°C (antibodies used in this study are listed below). After four washes (3 × 5 min + 1 × 30 min) in PBS-0.1%T, embryos were incubated in secondary antibodies diluted in blocking solution at a concentration of 1:300 for 1 h at room temperature and transferred through several washes of PBS-0.1%T before staining the nuclei with 4′,6-diamidino-2-phenylindole (DAPI; 0.5 µg/mL). Embryos were analyzed by confocal imaging (DM 6000 CS SP5; Leica). Acquisitions were performed using a water immersion objective (25×/1.25 0.75, PL APO HCX; Leica).

E8 rabbit post-implantation embryos were fixed in 2% PFA at 4 °C for 1 h. For cryoprotection, embryos were placed in 10% sucrose for 1 h followed by 30% sucrose overnight at 4 °C. Embryos were embedded in NEG50 compound (Invitrogen) and frozen at −80 °C. Sections of 15–20 µm were generated using a Microm HM550 cryostat and maintained at −80 °C until immunostaining was performed. For immunostaining, sections were thawed for 30 min at room temperature, saturated with PBS for 15 min at room temperature, and permeabilized (as necessary) with three baths of PBS containing 0.5% Triton X100 (Sigma-Aldrich). Sections were incubated in blocking solution: PBS with 0.1% Triton X100 and 10% donkey serum (Jackson Immunoresearch Laboratory) for 30 min. Subsequently, the sections were incubated with primary antibodies overnight at 4 °C. After several washes, the sections were incubated with the appropriate fluorescence-conjugated secondary antibodies at room temperature for 1 h. Nuclei were stained with DAPI (0.5 µg/mL). Sections were mounted on coverslips with Fluoromount G (Thermo Fisher Scientific). Tiled scans were automatically acquired using the LAS AF software (Leica).

Cells were fixed with 2% PFA in PBS at room temperature for 20 min, washed thrice (10 min each) with PBS and permeabilized in PBS containing 0.5% Triton X100. Non-specific binding sites were blocked using PBS supplemented with 10% donkey serum for 1 h at room temperature. The cells were incubated overnight at 4 °C with primary antibodies. After three rinses (10 min each) with PBS, the cells were incubated with secondary antibodies at room temperature for 1 h. The nuclei were stained with DAPI (0.5 µg/mL). The cells were mounted on coverslips by using the mounting medium M1289 (Sigma). Cells and embryo sections were analyzed by confocal imaging (DM 6000 CS SP5; Leica). Acquisitions were performed using an oil immersion objective (45×/1.25 0.75, PL APO HCX; Leica).

EdU staining was performed using the Click-iT™ Edu Imaging kit (Fisher Scientific). Briefly, embryos microinjected with PSCs were incubated for 1 h with 10 µM EdU in RDH medium. Embryos were fixed with 2–4% PFA, and immunostaining was performed according to the instructions provided by the manufacturer. Embryos were analyzed by confocal imaging (DM 6000 CS SP5; Leica), and acquisitions were performed using a water immersion objective (25×/1.25 0.75, PL APO HCX; Leica).

Two-photon recording
Embryos microinjected with rhesus and mESCs were placed in drops (5 µL) of medium and covered with liquid paraffin (Origio). For recording, embryos were cultured under the inverted Axio-Observer Z1 (Zeiss) two-photon microscope for 3 days, until they reach the E5 stage. Images were acquired using the Zeiss Zen software, and image analysis was performed using the ImageJ software.

Flow cytometry analysis of cell cycle distribution

Cells were labeled for 15 minutes with 10µM EdU in the culture medium using the Click-it Plus EdU flow cytometry kit (ThermoFischer). Cells were then washed and processed for the detection of incorporated EdU according to the manufacturer's instructions. After immunostaining, cells were incubated for 20 min with 1 mg/ml RNAse in PBS-0.13 mM EGTA. Propidium iodide (1 µg/ml) was added just before analysis. Cells were analyzed using a FACS LSR II (Becton-Dickinson) equipped with 355, 488, and 561 nM lasers. Data were acquired and analyzed using DiVa software.

RNA sequencing

RNA from cell lines was extracted from 4–5.10^6 cells using the RNeasy mini-kit (Qiagen). The libraries were prepared using 200 ng of RNA with the NextFlex Rapid Directional mRNA-Seq kit (Bioo-Scientific). Samples were sequenced on a NextSeq500 sequencing machine (Illumina) as single reads of 75 bp. The bcl2fastq conversion software was used for demultiplexing (Illumina), and data trimming was performed using Cutadapt. The sequencing depth for each sample was approximately 30 million reads, which were subsequently mapped to the Mmul8 rhesus macaque genome using the HISAT2 alignment program and quantified with the HTseq script.

Bioinformatics analysis

RNA-seq data obtained from rhesus primed and naïve cell lines were analyzed using the R software. PCA, differential expression analysis (with false discovery rate <0.1), and hierarchical clustering were performed with DESeq2 (Love et al., 2014). For cell lines, PCA was performed using the top 500 genes selected by highest row variance. For the comparison of cell lines with embryo data, we used the Seurat R package (version 3.1). Briefly, the two compendia of datasets were transformed to a Seurat object and merged. The transcript counts were subsequently log-transformed and normalized. Finally, the variable genes (3,000) were used as input for PCA.

Table S1: Summary of interspecies chimera experiments. Related to Figure 1, 2, 4 and 5.

	Rabbit host		
	E3 (1 DIV)	E4 (2 DIV)	E5 (3 DIV)
Rhesus PSCs			
Primed			
**Nb GFP+ embryos/injected embryos**	**10/104**	**0/89**	**0/90**
Nb NANOG+ embryos/GFP+ embryos	0/7		
Nb SOX2+ embryos/GFP+ embryos	0/2		
Nb GATA6+ embryos/GFP+ embryos	0/1		
TL2i			
**Nb GFP+ embryos/injected embryos**	**109/141**	**78/145**	**79/204**
Nb NANOG+ embryos/GFP+ embryos	14/16	7/12	2/7
Nb SOX2+ embryos/GFP+ embryos	45/53	30/52	8/40
Nb GATA6+ embryos/GFP+ embryos	6/31	8/22	14/33
4i/L/b			
**Nb GFP+ embryos/injected embryos**	**45/59**	**32/65**	**30/104**
Nb NANOG+ embryos/GFP+ embryos	5/7	3/12	1/7
Nb SOX2+ embryos/GFP+ embryos	3/5	0/1	1/6
Nb GATA6+ embryos/GFP+ embryos	4/33	5/19	6/17
t2iLGoY			
**Nb GFP+ embryos/injected embryos**	**92/96**	**72/97**	**69/114**
Nb NANOG+ embryos/GFP+ embryos	10/40	0/21	0/11
Nb SOX2+ embryos/GFP+ embryos	14/43	6/31	3/10
Nb GATA6+ embryos/GFP+ embryos	3/39	10/38	8/20
LCDM (EPS)			
**Nb GFP+ embryos/injected embryos**	**66/89**	**36/89**	**44/157**
Nb NANOG+ embryos/GFP+ embryos	0/19	2/12	10/17
Nb SOX2+ embryos/GFP+ embryos	20/31	11/15	5/20
Nb GATA6+ embryos/GFP+ embryos	1/24	0/13	2/19
E_NHSM			
**Nb GFP+ embryos/injected embryos**	**15/71**	**1/70**	**0/71**
Nb NANOG+ embryos/GFP+ embryos	0/24		
Nb SOX2+ embryos/GFP+ embryos	0/20		
Nb GATA6+ embryos/GFP+ embryos	2/27		
NHSM_v			
**Nb GFP+ embryos/injected embryos**	**11/68**	**8/84**	**10/118**
Nb NANOG+ embryos/GFP+ embryos	0/3		
Nb SOX2+ embryos/GFP+ embryos	1/3	0/3	0/7
Nb GATA6+ embryos/GFP+ embryos	0/5	0/5	1/3
TL_CDK8/19ii			
**Nb GFP+ embryos/injected embryos**	**43/79**	**19/80**	**10/101**
Nb NANOG+ embryos/GFP+ embryos	10/20	2/7	1/4
Nb SOX2+ embryos/GFP+ embryos	2/12	2/11	1/5
Nb GATA6+ embryos/GFP+ embryos	6/15	1/3	0/1
Mouse ESCs			
Serum + LIF			
**Nb GFP+ embryos/injected embryos**			**235/238**
Nb NANOG+ embryos/GFP+ embryos			77/77
Nb SOX2+ embryos/GFP+ embryos			58/58
Nb SOX17+ embryos/GFP+ embryos			0/30
2iLIF			
Nb GFP+ embryos/injected embryos			19/29
Nb SOX2+ embryos/GFP+ embryos			7/7
Nb SOX17+ embryos/GFP+ embryos			0/7
**Total**	**775**	**786**	**1226**
**Total injected embryos**	**2787**		

	Rabbit host		
	E3 (1 DIV)	E4 (2 DIV)	E5 (3 DIV)
Human PSCs			
hiPSCs_t2iLGoY			
**Nb GFP+ embryos/injected embryos**	**27/29**	**20/31**	**97/350**
Nb NANOG+ embryos/GFP+ embryos			5/23
Nb SOX2+ embryos/GFP+ embryos	24/29'	11/20	45/97
Nb GATA6+ embryos/GFP+ embryos			3/14
Nb SOX17+ embryos/GFP+ embryos			15/30
Nb T-BRA+ embryos/GFP+ embryos			4/11
Nb NESTIN+ embryos/GFP+ embryos			0/5
hiPSCs_TL2i			
**Nb GFP+ embryos/injected embryos**	**33/39**	**18/36**	**75/298**
Nb NANOG+ embryos/GFP+ embryos	15/15	3/7	0/9
Nb SOX2+ embryos/GFP+ embryos	9/11	4/8	2/33
Nb GATA6+ embryos/GFP+ embryos	1/7	2/3	8/22
Nb SOX17+ embryos/GFP+ embryos			26/30
Nb T-BRA+ embryos/GFP+ embryos			0/7
Nb NESTIN+ embryos/GFP+ embryos			0/5
**Total**	**68**	**67**	**648**
**Total injected embryos**	**783**		

BCL2 inducible cell lines

	Rabbit host	
	E5 (3 DIV)	
hiPSCs_t2iLGoY_BCL2		
	**-Dox**	**+Dox**
**Nb GFP+ embryos/injected embryos**	**20/66**	**82/82**
Nb NANOG+ embryos/GFP+ embryos	10/26	15/27
Nb SOX2+ embryos/GFP+ embryos	8/20	0/28
Nb GATA6+ embryos/GFP+ embryos	4/20	4/27
hiPSCs_TL2i_BCL2		
	**-Dox**	**+Dox**
**Nb GFP+ embryos/injected embryos**	**26/77**	**62/76**
Nb NANOG+ embryos/GFP+ embryos	0/26	0/26
Nb SOX2+ embryos/GFP+ embryos	13/26	3/25
Nb GATA6+ embryos/GFP+ embryos	25/25	19/25
**Total**	**143**	**158**
**Total injected embryos**	**301**	

	Rabbit host	
	E6	E9
Mouse ESCs		
**Nb GFP+ embryos/injected embryos**	**9/9**	**12/20**
Nb SOX2+ embryos/GFP+ embryos	6/6	
Nb SOX17+ embryos/GFP+ embryos	3/3	
Rhesus TL2i		
**Nb GFP+ embryos/injected embryos**		**0/16**
**Total**	**18**	**36**
**Total injected embryos**	**54**	

	Cynomolgus host
	E7
Rhesus TL2i	
**Nb GFP+ embryos/injected embryos**	**2/7**
Nb NANOG+ embryos/GFP+ embryos	0/2
hiPSCs_TL2i	
**Nb GFP+ embryos/injected embryos**	**2/7**
Nb NANOG+ embryos/GFP+ embryos	0/2
hiPSCs_t2iLGoY	
**Nb GFP+ embryos/injected embryos**	**2/15**
Nb NANOG+ embryos/GFP+ embryos	1/2
Mouse ESCs	
**Nb GFP+ embryos/injected embryos**	**3/7**
Nb NANOG+ embryos/GFP+ embryos	1/1
Nb SOX2+ embryos/GFP+ embryos	2/2
**Total injected embryos**	**36**

Total embryos injected in this study : 3961

Summary of culture media used in this study

	E-NHSM	NHSM-v	4i/L/b	TL2i	t2iLGöY	LCDM (EPS)	TL-CDK8/19i	5i/L/A
**Media**								
Neurobasal:DMEMF12 (1:1)	✓				✓	✓		✓
KO-DMEM		✓	✓	✓			✓	
N2B27	✓				✓	✓		✓
KOSR	0.5%	20%	20%	20%			20%	0.5%
**Transgene**								
STAT3-ERT2				✓			✓	
**Growth factors**								
LIF	10ng/ml	10ng/ml	10ng/ml	10,000U/ml	10ng/ml	10ng/ml	10,000U/ml	10ng/ml
Activin A	20ng/ml							20ng/ml
bFGF		8ng/ml	4ng/ml					
TGFb1		1ng/ml						
**Small molecules**								
BIRB796	2µM							
CGP77675	1µM							
CHIR99021	1.5µM	3µM	3µM	3µM		1µM		
PD0325901	1µM	1µM	0.5µM	1µM	1µM			1µM
SP600125	5µM	10µM	10µM					
Gö6983	2µM				1µM			
SB202190		5µM						
SB203580		10µM	10µM					
(S)-(+)-Dimethindene maleate						2µM		
Minocycline hydrochloride						2µM		
IWR1	5µM					0.75µM		
XAV939					2µM			
CDK8/19i							1.1µM	
IM-12								1µM
SB590885								0.5µM
WH-4-023								1µM

List of antibodies

Target	Supplier	Reference	Host species	Dilution for cells	Dilution for pre-implantation embryo	Dilution for cryosection	Dilution for immunoblotting
GFP	Thermo Fischer	A10262	Chicken	1:300	1:300	1:300	NA
mCherry	Thermo Fischer	M11217	Rat	1:300	1:300	NA	NA
OCT4	Santa Cruz	sc-9081	Rabbit	1:300	1:300	1:300	NA
NANOG	R&D Systems	AF1997	Goat	1:100	1:100	1:100	NA
NANOG	R&D Systems	AF2729	Goat	NA	1:100	1:100	NA
SOX2	R&D Systems	AF2018	Goat	1:100	1:100	1:100	NA
SOX17	R&D Systems	AF1924	Goat	1:100	1:100	NA	NA
GATA6	R&D Systems	AF1700	Goat	1:100	1:100	NA	NA
SUSD2-PE	BioLegend	327406	Mouse	1:100	NA	NA	NA
KLF4	Tebu-Bio	09-0021	Mouse	1:100	NA	NA	NA
TFE3	Thermo Fischer	PA5-21615	Rabbit	1:250	NA	NA	NA
KLF17	Sigma-Aldrich	HPA024629	Rabbit	1:100	NA	NA	NA
HuN	Sigma-Aldrich	MAB1281	Mouse	NA	1:100	NA	NA
TuJ1	Sigma-Aldrich	T2200	Rabbit	NA	NA	1:100	NA
H3K27me3	Cell Signalling	9733	Rabbit	1:1000	NA	NA	1/1000
TFAP2C	R&D Systems	AF5059	Goat	1:100	NA	NA	1/1000
TFCP2L1	R&D Systems	AF5726	Goat	1:100	NA	NA	1/1000
CDK2	Cell Signalling	E8J9T	Rabbit	NA	NA	NA	1/1000
CDK4	Abcam	Ab199728	Rabbit	NA	NA	NA	1/1000
CYCLIN E1	Cell Signalling	20808	Rabbit	NA	NA	NA	1/1000
CYCLIN A2	Thermo Fischer	MA1-154	Mouse	NA	NA	NA	1/1000
CYCLIN D1	Cell Signalling	55506	Rabbit	NA	NA	NA	1/1000
CYCLIN D2	Cell Signalling	3741	Rabbit	NA	NA	NA	1/1000
CYCLIN D3	Cell Signalling	2936	Mouse	NA	NA	NA	1/1000
p27kip1	Cell Signalling	3688	Rabbit	NA	NA	NA	1/1000
ACTINB	Sigma-Aldrich	A3854	Mouse	NA	NA	NA	1/10000

References

CHEN, H., AKSOY, I., GONNOT, F., OSTEIL, P., AUBRY, M., HAMELA, C., ROGNARD, C., HOCHARD, A., VOISIN, S., FONTAINE, E., MURE, M., AFANASSIEFF, M., CLEROUX, E., GUIBERT, S., CHEN, J., VALLOT, C., ACLOQUE, H., GENTHON, C., DONNADIEU, C., DE VOS, J., SANLAVILLE, D., GUÉRIN, J.-F., WEBER, M., STANTON, L. W., ROUGEULLE, C., PAIN, B., BOURILLOT, P. & SAVATIER, P. 2015a. Reinforcement of STAT3 activity reprogrammes human embryonic stem cells to naïve-like pluripotency. *Nat Commun,* 6**,** 7095-7112.

CHEN, Y., NIU, Y., LI, Y., AI, Z., KANG, Y., SHI, H., XIANG, Z., YANG, Z., TAN, T., SI, W., LI, W., XIA, X., ZHOU, Q., JI, W. & LI, T. 2015b. Generation of Cynomolgus Monkey Chimeric Fetuses using Embryonic Stem Cells. *Cell Stem Cell,* 17**,** 116-124.

FANG, R., LIU, K., ZHAO, Y., LI, H., ZHU, D., DU, Y., XIANG, C., LI, X., LIU, H., MIAO, Z., ZHANG, X., SHI, Y., YANG, W., XU, J. & DENG, H. 2014. Generation of naive induced pluripotent stem cells from rhesus monkey fibroblasts. *Cell Stem Cell,* 15**,** 488-96.

GAFNI, O., WEINBERGER, L., MANSOUR, A. A., MANOR, Y. S., CHOMSKY, E., BEN-YOSEF, D., KALMA, Y., VIUKOV, S., MAZA, I., ZVIRAN, A., RAIS, Y., SHIPONY, Z., MUKAMEL, Z., KRUPALNIK, V., ZERBIB, M., GEULA, S., CASPI, I., SCHNEIR, D., SHWARTZ, T., GILAD, S., AMANN-ZALCENSTEIN, D., BENJAMIN, S., AMIT, I., TANAY, A., MASSARWA, R., NOVERSHTERN, N. & HANNA, J. H. 2013. Derivation of novel human ground state naive pluripotent stem cells. *Nature,* 504**,** 282-286.

GUO, G., VON MEYENN, F., ROSTOVSKAYA, M., CLARKE, J., DIETMANN, S., BAKER, D., SAHAKYAN, A., MYERS, S., BERTONE, P., REIK, W., PLATH, K. & SMITH, A. 2017. Epigenetic resetting of human pluripotency. *Development,* 144**,** 2748-2763.

LOVE, M. I., HUBER, W. & ANDERS, S. 2014. Moderated estimation of fold change and dispersion for RNA-seq data with DESeq2. *Genome Biol,* 15**,** 550.

NG, S. Y., JOHNSON, R. & STANTON, L. W. 2012. Human long non-coding RNAs promote pluripotency and neuronal differentiation by association with chromatin modifiers and transcription factors. *EMBO J,* 31**,** 522-33.

SAKAUE-SAWANO, A., YO, M., KOMATSU, N., HIRATSUKA, T., KOGURE, T., HOSHIDA, T., GOSHIMA, N., MATSUDA, M., MIYOSHI, H. & MIYAWAKI, A. 2017. Genetically Encoded Tools for Optical Dissection of the Mammalian Cell Cycle. *Mol Cell,* 68**,** 626-640 e5.

THEUNISSEN, T. W., POWELL, B. E., WANG, H., MITALIPOVA, M., FADDAH, D. A., REDDY, J., FAN, Z. P., MAETZEL, D., GANZ, K., SHI, L., LUNGJANGWA, T., IMSOONTHORNRUKSA, S., STELZER, Y., RANGARAJAN, S., D'ALESSIO, A., ZHANG, J., GAO, Q., DAWLATY, M. M., YOUNG, R. A., GRAY, N. S. & JAENISCH, R. 2014. Systematic Identification of Culture Conditions for Induction and Maintenance of Naive Human Pluripotency. *Cell Stem Cell,* 15**,** 471-487.

WIANNY, F., BERNAT, A., MARCY, G., HUISSOUD, C., MARKOSSIAN, S., LEVIEL, V., KENNEDY, H., SAVATIER, P. & DEHAY, C. 2008. Derivation and cloning of a novel Rhesus ES cell line stably expressing tau-GFP. *Stem Cells,* 26**,** 1444-1453.

YANG, J., RYAN, D. J., WANG, W., TSANG, J. C., LAN, G., MASAKI, H., GAO, X., ANTUNES, L., YU, Y., ZHU, Z., WANG, J., KOLODZIEJCZYK, A. A., CAMPOS, L. S., WANG, C., YANG, F., ZHONG, Z., FU, B., ECKERSLEY-MASLIN, M. A., WOODS, M., TANAKA, Y., CHEN, X., WILKINSON, A. C., BUSSELL, J., WHITE, J., RAMIREZ-SOLIS, R., REIK, W., GOTTGENS, B., TEICHMANN, S. A., TAM, P. P. L., NAKAUCHI, H., ZOU, X., LU, L. & LIU, P. 2017. Establishment of mouse expanded potential stem cells. *Nature,* 550**,** 393-397.
