# Supplementary material for: Apoptosis, G1 Phase Stall, and Premature Differentiation Account for Low Chimeric Competence of Human and Rhesus Monkey Naive Pluripotent Stem Cells
Source: Stem Cell Reports. 2020 Dec 30;16(1):56–74. doi: 10.1016/j.stemcr.2020.12.004 (PMC7815945; doi:10.1016/j.stemcr.2020.12.004)
Supplement: Document S2. Article plus Supplemental Information [file mmc8.pdf]

Stem Cell Reports
Article
OPEN ACCESS

# Apoptosis, G1 Phase Stall, and Premature Differentiation Account for Low Chimeric Competence of Human and Rhesus Monkey Naive Pluripotent Stem Cells

Irène Aksoy,[1,*] Cloé Rognard,[1] Anaïs Moulin,[1] Guillaume Marcy,[1] Etienne Masfaraud,[1] Florence Wianny,[1] Véronique Cortay,[1] Angèle Bellemin-Ménard,[1] Nathalie Doerflinger,[1] Manon Dirheimer,[1] Chloé Mayère,[1] Pierre-Yves Bourillot,[1] Cian Lynch,[4] Olivier Raineteau,[1] Thierry Joly,[2,3] Colette Dehay,[1] Manuel Serrano,[4] Marielle Afanassieff,[1] and Pierre Savatier[1,*]
[1]Univ Lyon, Université Lyon 1, INSERM, Stem Cell and Brain Research Institute U1208, 69500 Bron, France
[2]ISARA-Lyon, 69007 Lyon, France
[3]VetAgroSup, UPSP ICE, 69280 Marcy l'Etoile, France
[4]Cellular Plasticity and Disease Group, Institute for Research in Biomedicine (IRB Barcelona), Barcelona Institute of Science and Technology (BIST), Barcelona 08028, Spain
*Correspondence: irene.aksoy@inserm.fr (I.A.), pierre.savatier@inserm.fr (P.S.)
https://doi.org/10.1016/j.stemcr.2020.12.004

## SUMMARY

After reprogramming to naive pluripotency, human pluripotent stem cells (PSCs) still exhibit very low ability to make interspecies chimeras. Whether this is because they are inherently devoid of the attributes of chimeric competency or because naive PSCs cannot colonize embryos from distant species remains to be elucidated. Here, we have used different types of mouse, human, and rhesus monkey naive PSCs and analyzed their ability to colonize rabbit and cynomolgus monkey embryos. Mouse embryonic stem cells (ESCs) remained mitotically active and efficiently colonized host embryos. In contrast, primate naive PSCs colonized host embryos with much lower efficiency. Unlike mouse ESCs, they slowed DNA replication after dissociation and, after injection into host embryos, they stalled in the G1 phase and differentiated prematurely, regardless of host species. We conclude that human and non-human primate naive PSCs do not efficiently make chimeras because they are inherently unfit to remain mitotically active during colonization.

## INTRODUCTION

Human embryo-derived pluripotent stem cells (PSCs) and human induced PSCs (iPSCs) exhibit biological and functional characteristics of primed pluripotency: (1) dependence on fibroblast growth factor 2 (FGF2)/extracellular signal-regulated kinase and activin A/SMAD signaling for self-renewal; (2) inactivation of the second X chromosome in female lines; and (3) a global transcriptome more similar to that of post-implantation epiblast in the gastrulation embryo (Chen and Lai, 2015; Davidson et al., 2015; Nakamura et al., 2016; Nichols and Smith, 2009). Although PSC lines obtained from rhesus monkeys are less well characterized, they also exhibit the essential characteristics of primed pluripotency (Wianny et al., 2008). In this respect, human and non-human primate PSCs differ from their murine counterparts, which exhibit biological and functional characteristics of naive pluripotency (Chen and Lai, 2015; Davidson et al., 2015; Nichols and Smith, 2009). Notably, naive and primed PSCs differ in their capacity to generate chimeras following injection into pre-implantation embryos. Although mouse ESCs (mESCs) produce germline chimeras, rhesus monkey PSCs do not colonize the inner cell mass (ICM)/epiblast of the host embryo and undergo apoptosis (Tachibana et al., 2012).

Different culture media with capacity to produce primed-to-naive conversion of human, cynomolgus, and rhesus macaque PSCs have been reported. The media are variously termed naive human stem cell medium (NHSM) (Gafni et al., 2013), E-NHSM (https://hannalabweb.weizmann.ac.il), NHSM-v (Chen et al., 2015b), 3iL (Chan et al., 2013), Reset (Takashima et al., 2014), 5i/L/A and 6i/L/A (Theunissen et al., 2014, 2016), 4i/L/b (Fang et al., 2014), TL2i (Chen et al., 2015a), 2iLD, 4i, and FAC (Wu et al., 2017), t2iLGöY (Guo et al., 2017), and LCDM (Yang et al., 2017b). PSCs under these culture conditions display characteristic features of naive-state pluripotency of mESCs with a reconfigured transcriptome and epigenome (Chen et al., 2015a; Huang et al., 2014; Nakamura et al., 2016), loss of FGF2 dependency (Chen et al., 2015a; Takashima et al., 2014), gain of STAT3 dependency (Chan et al., 2013; Chen et al., 2015a; Gafni et al., 2013; Takashima et al., 2014), reactivation of the second X chromosome (Fang et al., 2014; Takashima et al., 2014; Theunissen et al., 2014), and elevation of oxidative phosphorylation (Takashima et al., 2014; Ware et al., 2009).

Colonization of mouse embryos by human naive PSCs and their subsequent participation in germ layer differentiation has been reported (Fang et al., 2014; Gafni et al., 2013). However, these early results were not confirmed by subsequent reports: low rates of chimerism (<0.001%) in


This is an open access article under the CC BY license (http://creativecommons.org/licenses/by/4.0/).

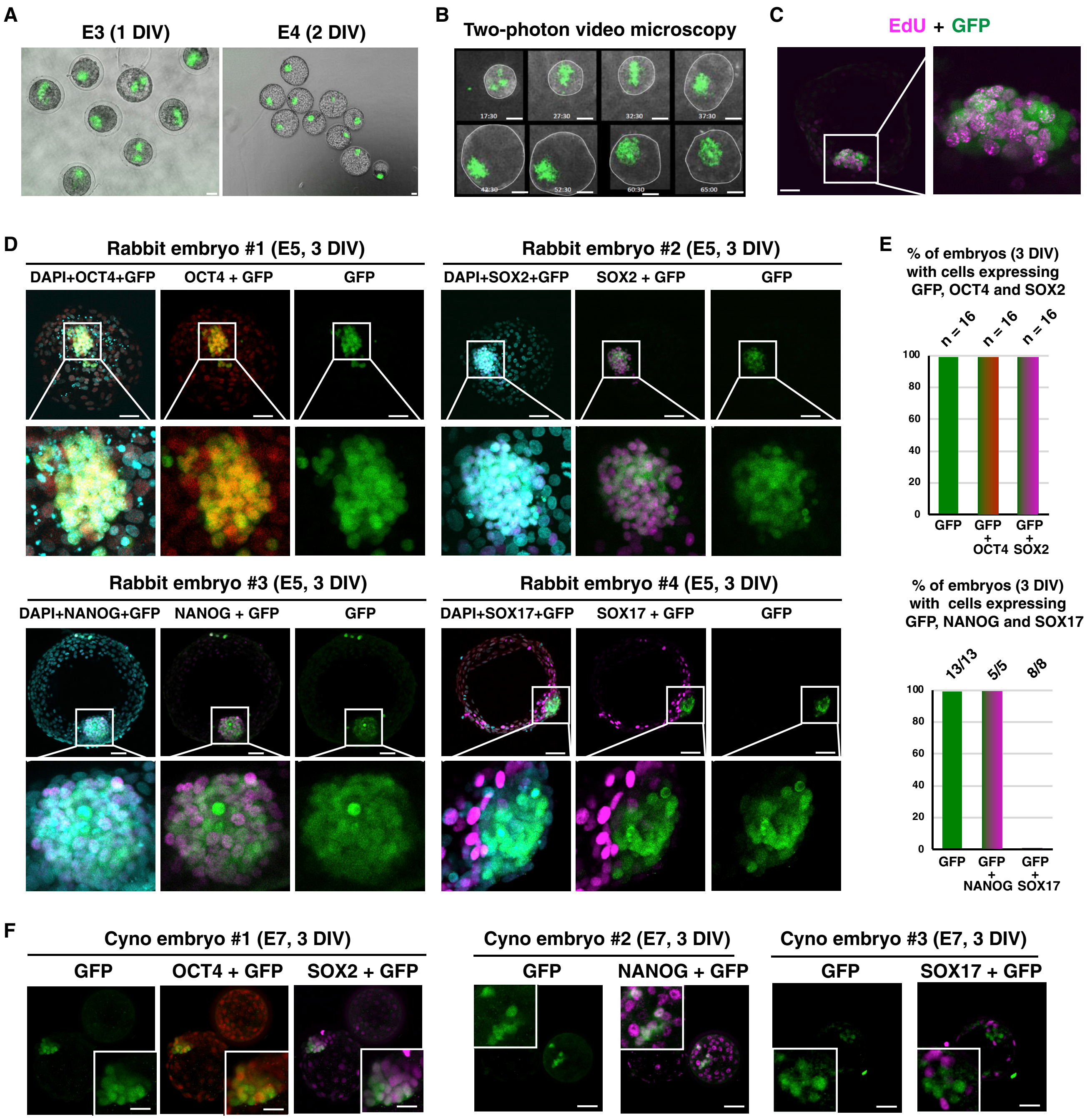


**Figure 1. Colonization of Rabbit and Cynomolgus Embryos by mESCs**

(A) Epifluorescence images of the early- (E3, 1 DIV) and mid-blastocyst-stage rabbit embryos (E4, 2 DIV) resulting from microinjection of 10 mESC-GFP cells. Scale bars, 50 μm.

(B) Two-photon microscope images of the late blastocyst-stage rabbit embryos (E2–E5, 0–3 DIV) resulting from microinjection of 10 mESC-GFP cells. Scale bars, 50 μm.

(C) Immunostaining of GFP and EdU in a late blastocyst-stage rabbit embryo (E5, 3 DIV) after microinjection of 10 mESC-GFP cells into the morula-stage (E2) embryo (confocal imaging). Scale bar, 50 μm.

(D) Immunostaining of GFP, OCT4, SOX2, NANOG, and SOX17 of late blastocyst-stage rabbit embryos (E5, 3 DIV) after microinjection of 10 mESC-GFP cells into morula-stage (E2) embryos (confocal imaging; n = 135). Scale bars, 50 μm.

*(legend continued on next page)*

<1% of the fetuses were reported after injection of human naive PSCs in mouse blastocysts (Masaki et al., 2015; Theunissen et al., 2016). Similarly, very low rates of chimerism were also observed in pig fetuses after injecting human naive iPSCs in pig embryos (Wu et al., 2017). Whether human PSCs have failed to produce chimeras because naive PSCs in general cannot colonize embryos from distant species or because human PSCs are inherently devoid of the attributes of chimeric competence is not known.

To explore this issue, we investigated the differential ability of mESCs, and rhesus monkey and human naive PSCs, to colonize both closely and distantly related host embryos. To circumvent the ban on introducing human embryo-derived PSCs into animal embryos, we used embryo-derived rhesus monkey PSCs and human iPSCs. As a reference, we explored the ability of mESCs, the gold standard of naive pluripotency, to colonize evolutionary distant host embryos. As host species, we selected rabbit and cynomolgus monkey embryos. Primates and glires (rodents + lagomorphs) diverged between 85 and 97 million years ago, while rodents and lagomorphs diverged between 77 and 88 million years ago (www.timetree.org). The divergence time makes rabbit embryos an almost equidistant environment for comparing the colonizing capabilities of rodent and primate PSCs. In addition, rabbits have several advantages in testing the colonization capacity of human PSCs. The early rabbit embryos share common features with primate embryos in their developmental characteristics (Madeja et al., 2019); unlike the three-dimensional egg-cylinder shape of rodent embryos during gastrulation, primate and rabbit embryos develop into a flattened disc at the surface of the conceptus; primate and rabbit embryos are also markedly similar with respect to the timing of zygotic genome activation and the timing and regulation of X chromosome inactivation. In rodents and primates, gastrulation occurs in the implanted embryo buried within the uterine wall. However, in rabbits, gastrulation begins shortly before implantation, allowing easier access to a wider developmental window. Furthermore, the rabbit PSC lines derived from pre-implantation embryos require FGF2 and transforming growth factor β for inhibition of differentiation and exhibit the cardinal features of primed pluripotency (Osteil et al., 2013, 2016). Thus, rabbit pre-implantation embryos appear to be more similar to primate embryos than mouse embryos with respect to the mechanisms regulating pluripotency. Therefore, they may be a better host for examining the colonization competence of human PSCs. As a closely related host for human and rhesus monkey PSCs, we used cynomolgus macaque embryos. The common ancestor of macaques and humans dates back 29 million years, while rhesus and cynomolgus macaques diverged 3.7 million year ago (http://www.timetree.org). We explored the differential ability of mESCs, rhesus monkey PSCs, and human naive iPSCs to colonize rabbit and cynomolgus embryos, and drew conclusions on the inherent abilities of rodent and primate naive PSCs to colonize distantly related hosts.

## RESULTS

### Colonization of Rabbit Embryos by mESCs

Fresh rabbit embryos observed by epifluorescence microscopy typically show a high level of autofluorescence (Figure S1A), which could be confounding for the detection of GFP-expressing cells in chimeras. To overcome this limitation, we systematically used an anti-GFP antibody. No immunostaining was observed when applied to rabbit embryos (1) before any injection of cells, and (2) after injection of ten rhesus wild-type PSCs (LyonES [Wianny et al., 2008]) that do not express any fluorescent reporters. Immunolabeling was observed in both rhesus PSCs expressing GFP as a fusion between tau protein and GFP (LyonES-tauGFP) (Figure S1B) and rabbit embryos colonized with naive-like rabbit iPSCs expressing GFP (Figure S1C) (Osteil et al., 2016). Thus, immunolabeling of GFP allows an effective detection of GFP expression in our system.

Mouse ESCs grown in serum/leukemia inhibitory factor (LIF) and, in particular, in 2i/LIF epitomize naive pluripotency and, for this reason, we tested their ability to colonize rabbit embryos. For this purpose, ten mESC-GFP cells cultured in serum/LIF condition were injected into rabbit embryos at the morula stage (embryonic day 2 [E2]). The morulas were subsequently cultured for 1–3 days *in vitro* (DIV) and developed into early (E3), mid (E4), and late (E5) blastocysts (1, 2, and 3 DIV, respectively). The vast majority of rabbit blastocysts had incorporated groups of $GFP^+$ cells (97.5%, n = 81) (Figure 1A; Table S1). mESCs and their progeny divided very actively, as shown by both the expansion of the $GFP^+$ cell pool during *in vitro* culture of chimeric embryos and a high incorporation of 5-ethylnyl-2′-deoxyuridine (EdU) in most $GFP^+$ cells (Figures 1B and 1C; Video S1). It should be noted that, under the conditions used (very short incorporation time), the EdU labeling of host embryo cells is markedly low. $GFP^+$ cells and their progeny expressed the transcription factors and pluripotency markers OCT4, NANOG, and SRY-box transcription factor

(E) Histogram of percentage of rabbit embryos with $GFP^+/OCT4^+$, $GFP^+/SOX2^+$, $GFP^+/NANOG^+$, and $GFP^+/SOX17^+$ cells at 3 DIV (n = 74).
(F) Immunostaining of GFP, OCT4, SOX2, and SOX17 of late blastocyst-stage (E7) cynomolgus embryos after microinjection of 10 mESC-GFP cells into morula-stage (E4) embryos (confocal imaging; n = 7). Scale bars, 50 μm.

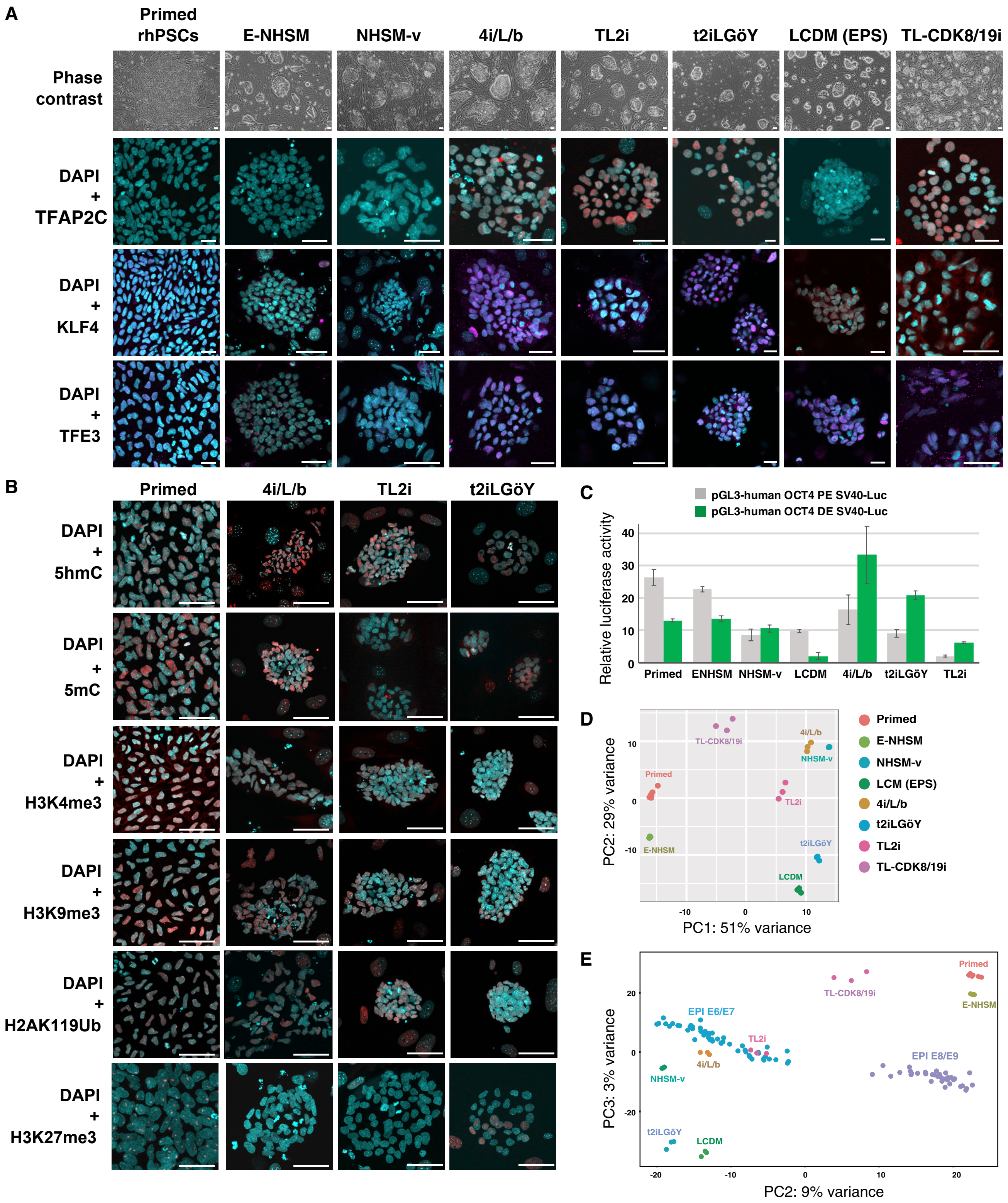


(legend on next page)

(SOX)2, as revealed by immunostaining at E5 (3 DIV) (Figures 1D and 1E). In contrast, GFP$^+$ cells did not express SOX17, a primitive endoderm marker, indicating that they did not differentiate to the primitive endoderm lineage. We performed a similar experiment using cynomolgus monkey embryos as hosts. For this purpose, 10 mESC-GFP cells were injected into cynomolgus embryos at the morula stage (E4). The morulas were subsequently cultured for 3 DIV and developed into mid/late (E7) blastocysts before immunostaining with GFP, OCT4, NANOG, SOX2, and SOX17 antibodies. Among the 5 embryos analyzed, 3 contained 8–16 GFP$^+$ cells in the ICM. The first embryo was immunostained with GFP, OCT4, and SOX2 antibodies and contained 16 GFP$^+$/OCT4$^+$/SOX2$^+$ cells. The second one was immunostained with GFP and NANOG antibodies and contained eight GFP$^+$/NANOG$^+$ cells. The third one was immunostained with GFP and SOX17 antibodies and contained eight GFP$^+$/SOX17$^-$ cells (Figure 1F; Table S1). In another experiment, ten mESC-GFP cells propagated in N2B27 supplemented with LIF, PD0325901, and CHIR99021 (i.e., 2i/LIF condition) were injected into rabbit morulas and subsequently cultured for 3 DIV before immunostaining (Figure S2A and Table S1). Of the 19 E5 blastocysts obtained, 14 had incorporated groups of GFP$^+$ cells. Immunostaining showed that 100% of chimeric embryos harbored GFP$^+$ cells expressing SOX2 (n = 7/7), whereas none of them expressed SOX17 (n = 0/7). Overall, these results show that mESCs, whether in serum/LIF or 2i/LIF conditions, continue to express pluripotency markers and expand after injection into evolutionary distant embryos, whether rabbit or cynomolgus monkey embryos.

We subsequently studied the ability of mESCs to colonize the embryonic disk of rabbit pre-gastrula embryos (E6) and the three germ layers after gastrulation (E9). For this purpose, the E2 embryos injected with 10 mESC-GFP cells (grown in serum/LIF) were transferred into the oviducts of surrogate rabbits. Nine embryos were recovered after 4 days (i.e., E6). All contained GFP$^+$ cells expressing OCT4 and SOX2 (Figure S2B and Table S1). Three embryos were immunostained with a SOX17 antibody and did not show SOX17$^+$ cells. On the other hand, the GFP$^+$ cells formed a coherent group of cells in the embryonic disk, suggesting that mouse cells did not mix with rabbit cells. To assess whether mESCs participated in the development of rabbit embryos after gastrulation, 20 fetuses were recovered 7 days after transfer to surrogate mothers (i.e., E9) (Figure S2C and Table S1). Fetuses contained GFP$^+$/SOX2$^+$, GFP$^+$/TUJ1$^+$, and GFP$^+$/NANOG$^-$ cells in the neurectoderm (n = 12/12). None of the fetuses had GFP$^+$ cells in embryonic tissues derived from the mesoderm and endoderm or in extra-embryonic tissues. These results strongly suggest that mESCs injected into rabbit morulas contribute to the expansion of the late epiblasts until the onset of gastrulation. After gastrulation, mESCs were able to contribute to the neuroectoderm, but not to other embryonic and extra-embryonic lineages.

## Reprogramming Rhesus PSCs to Naive Pluripotency

The LyonES rhesus line expressing a tauGFP transgene under the control of the ubiquitous CAG promoter has been described previously (Wianny et al., 2008). LyonES-tauGFP cells were infected with the pGAE-STAT3-ER$^{T2}$ lentiviral vector (Chen et al., 2015a), and a clonal cell line stably expressing STAT3-ER$^{T2}$ was isolated as LyonES-tGFP-(S3). Immunostaining with anti-STAT3 antibody showed increased labeling and nuclear translocation following the treatment of LyonES-tGFP-(S3) cells with tamoxifen (Figure S3A). LyonES-tGFP-(S3) cells were cultured in the presence of tamoxifen to activate STAT3-ER$^{T2}$ and with 1,000 U/mL of LIF, MEK, and GSK3β inhibitors. Consistent with previous observations in human PSCs (Chen et al., 2015a), the LyonES-tGFP-(S3) cells formed small dome-shaped colonies, which were termed rhesus TL2i (rhTL2i) cells (Figure 2A). These colonies demonstrated identical morphology with that described previously for human TL2i cells (Chen et al., 2015a). We developed a variant of the TL2i protocol, in which MEKi and GSK3βi were replaced by CNIO-47799, a chemical

**Figure 2. Characterization of Rhesus PSCs after Reprogramming to Naive Pluripotency**

(A) Phase contrast and immunostaining of naive pluripotency markers, TFAP2C, KLF4, and TFE3 in LyonES-tGFP-(S3) cells, before (primed rhesus PSCs) and after reprogramming to the naive state (E-NHSM, NHSM-v, 4i/L/b, TL2i, t2iLGöY, LCDM (EPS), and TL-CDK8/19i) (confocal imaging). Scale bars, 50 μm.

(B) Immunostaining of 5′-methylcytosine (5mC), 5′-hydroxymethylcytosine (5hmC), H3K4me3, H3K9me3, H2AK119Ub, and H3K27me3 in LyonES-tGFP-(S3) cells, before (primed) and after reprogramming to the naive state (4i/L/b, TL2i, and t2iLGöY) (confocal imaging). Scale bars, 50 μm.

(C) Activity of the proximal and distal enhancers (PE and DE, respectively) of *OCT4* measured in a luciferase assay after transient transfection in primed PSCs, E-NHSM, NHSM-v, 4i/L/b, TL2i, t2iLGöY, and LCDM cells.

(D) Principal-component analysis (PCA) for primed PSCs, E-NHSM, NHSM-v, 4i/L/b, TL2i, t2iLGöY, LCDM, and TL-CDK8/19i populations based on RNA sequencing (RNA-seq) data.

(E) PCA for primed PSCs, E-NHSM, NHSM-v, 4i/L/b, TL2i, t2iLGöY, LCDM, and TL-CDK8/19i populations based on bulk RNA-seq and single-cell RNA-seq data of cynomolgus epiblast from E6 to E9 embryos (Nakamura et al., 2016).

inhibitor of cyclin-dependent kinases (CDK)8 and CDK19 (CDK8/19i) for reprogramming to the naive state (Lynch et al., 2020). This protocol is referred to as TL-CDK8/19i and the reprogrammed cells as rhesus TL-CDK8/19i (rhTL-CDK8/19i). LyonES-tGFP-(S3) cells were submitted to six other reprogramming protocols previously developed to revert human or monkey PSCs to naive pluripotency, including NHSM-v and E-NHSM (Chen et al., 2015b; Gafni et al., 2013), 4i/L/b (Fang et al., 2014), t2iL-GöY (Guo et al., 2016; Takashima et al., 2014), 5iL/A or 6iL/A (Theunissen et al., 2014), and LCDM (extended pluripotent stem [EPS]) (Yang et al., 2017b). It should be noted that the six protocols were applied to LyonES-tGFP-(S3) cells in the absence of tamoxifen, thus maintaining STAT3-ER$^{T2}$ in an inactive state. LyonES-tGFP-(S3) cells differentiated or underwent apoptosis when cultured in LIF/MEKi/GSK3βi (not shown). Of the eight protocols tested, seven (i.e., NHSM-v, E-NHSM, 4i/L/b, t2iLGöY, LCDM [EPS], TL2i, and TL-CDK8/19i) produced rhesus PSCs forming dome-shaped colonies (Figure 2A). Continued instability was observed with protocol 5iL/A, which was subsequently omitted from the study. Unlike conventional rhesus PSCs, which were mechanically passaged in clumps, all reprogrammed cells were routinely dissociated into single cells using trypsin or trypLE. The colonies exhibited variable expression of naive transcription factors and cell surface molecules (Figures 2A, S3B and, S3G). Notably, the transcription factors AP-2 gamma (TFAP2C), KLF4, and transcription factor binding to IGHM enhancer 3 (TFE3) displayed stronger expression after reprogramming using the 4i/L/b, t2iLGöY, TL2i, and TL-CDK8/19i protocols. LCDM (EPS) cells expressed KLF4 and TFE3 but not TFAP2C. Cells reprogrammed with the other protocols did not express any of these naive markers (TFAP2C, KLF4, and TFE3). t2iLGöY, TL2i, and 4i/L/b cells featured lower levels of H3K4me3 marks (Figures 2B and S3C–D). They also displayed diffuse H3K27me3 immunostaining compared with punctate staining in primed, NHSM-v, and E-NHSM cells (Figures 2B, S3C, and S3E). Immunostaining of 5′-methylcytosine and 5′-hydroxymethylcytosine also revealed global genome hypomethylation in t2iLGöY, LCDM (EPS), and TL2i cells (Figures 2B, S3C, and S3D). Primed, E-NHSM, and NHSM-v cells exhibited punctate immunostaining for H2AK119Ub, indicating the presence of an inactive X chromosome, whereas t2iLGöY, LCDM (EPS), TL2i, and 4i/L/b cells displayed diffuse immunostaining, suggesting X chromosome reactivation (Figures 2B, S3C, and S3E). Altogether, these results strongly suggest that t2iLGöY, LCDM (EPS), TL2i, and 4i/L/b cells underwent epigenome reconfiguration, in line with the results of naive marker expression. Finally, consistent with their naive status, t2iLGöY, TL2i, and 4i/L/b cells exhibited preferential usage of the distal enhancer of Oct4 in a luciferase assay (Figure 2C).

To further characterize reprogramming, gene expression was analyzed by bulk RNA sequencing to assess transcriptome reconfiguration. Principal-component analysis and hierarchical clustering showed that primed and E-NHSM cells clustered on one side, whereas NHSM-v, 4i/L/b, t2iL-GöY, TL2i, and LCDM (EPS) cells clustered on the other side (Figure 2D; Figure S3F and Table S2). TL-CDK8/19i cells demonstrated an intermediate position, suggesting that transcriptome reconfiguration was less pronounced. A comparative analysis was performed between the primed and naive rhesus PSC lines and single-cell RNA sequencing data of the cynomolgus epiblast (Nakamura et al., 2016). The NHSM-v, 4i/L/b, t2iLGöY, TL2i, and LCDM (EPS) cells also clustered closer to the epiblast cells of the E6/E7 cynomolgus embryo (Nakamura et al., 2016) than the E-NHSM and primed cells (Figure 2E). These results show that, consistent with the immunostaining data, reprogramming of rhesus PSCs is characterized by a transcriptome shift reflecting the acquisition of characteristic features of the preimplantation epiblast as reported previously with human naive PSCs (Nakamura et al., 2016).

### Premature Differentiation of Rhesus Naive PSCs after Injection into Rabbit Embryos

The ability of reprogrammed rhesus PSCs to colonize rabbit ICM/epiblast was tested. For this purpose, ten LyonES-tGFP-(S3) cells of each type (primed, NHSM-v, E-NHSM, LCDM [EPS], 4i/L/b, t2iLGöY, TL2i, and TL-CDK8/19i) were injected into rabbit morulas (E2). The embryos were subsequently cultured for 1–3 DIV in gradual medium and developed into early (E3), mid (E4), and late blastocysts (E5). There was no difference observed in the rate of development between control (uninjected) and injected embryos (not shown). In the first set of experiments, the fate of injected cells was monitored by two-photon microscopy for 48–72 h. Contrary to our observations with mESCs, rhesus PSCs did not actively proliferate in host embryos, and most of them did not incorporate into ICM/epiblast (Videos S2, S3, S4, and S5). GFP$^+$ embryos and cell counts confirmed this finding: the number of embryos with GFP$^+$ cells decreased with time in culture, ranging from 0% (E-NHSM) to 62% (t2iLGöY) at 3 DIV compared with 22%–96% at 1 DIV (Figure 3A; Table S1). In addition, the percentage of embryos with $\geq$4 GFP$^+$ cells diminished in all experimental conditions tested, ranging from 0% (E-NHSM) to 24% (TL2i) at 3 DIV versus 0% (E-NHSM) to 79% (t2iLGöY) at 1 DIV (Figure 3B). These results indicate that rhesus PSCs failed to actively divide and/or were gradually eliminated during embryonic development, regardless of the reprogramming strategy used for conversion to the naive state. Despite the failure of the rhesus PSCs to

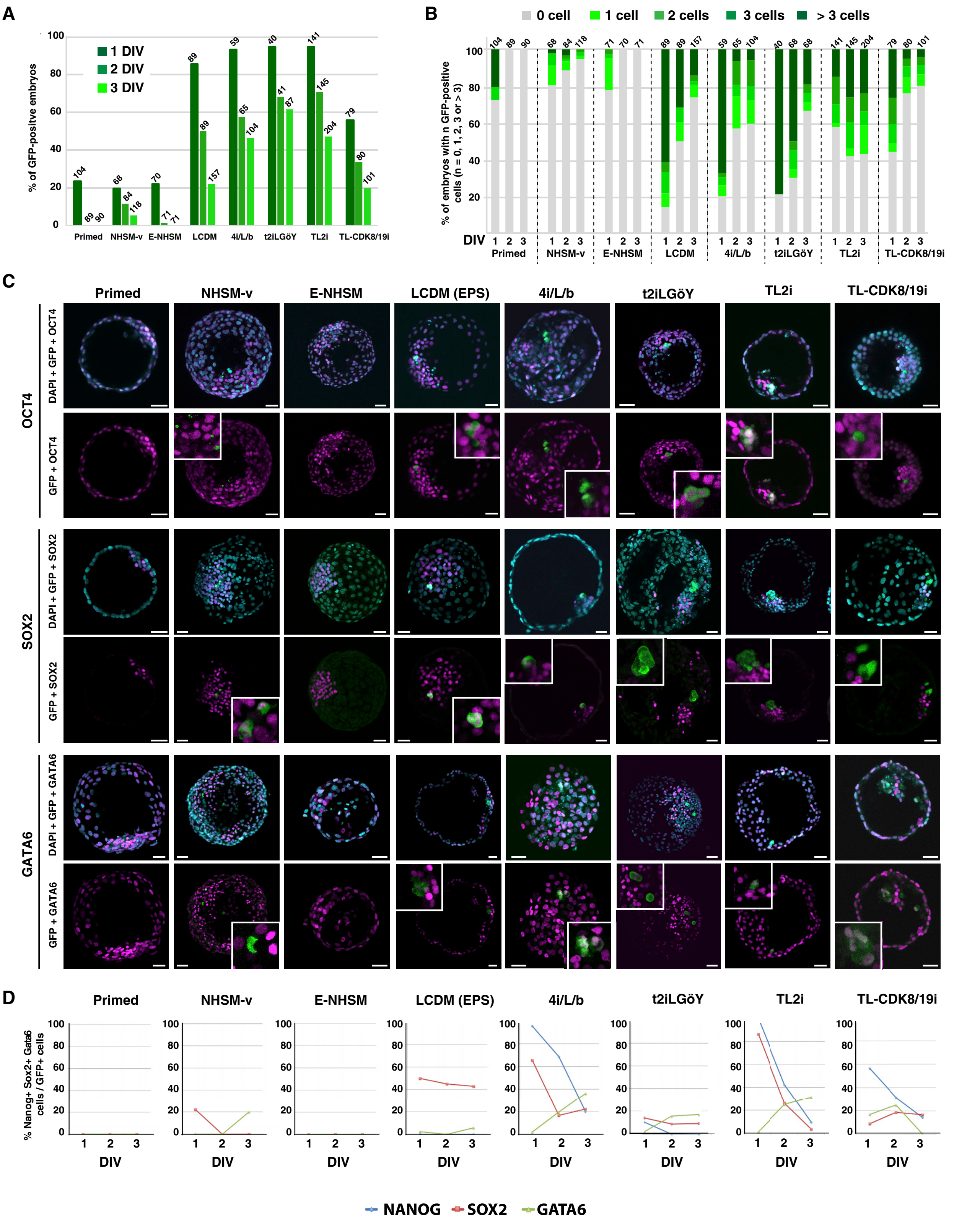


*(legend on next page)*

thrive in a rabbit embryo environment, variations in the survival rate of GFP+ cells at 3 DIV were observed between reprogramming strategies. There were no GFP+ cells observed in embryos injected with either primed rhesus PSCs (0/90) or E-NHSM cells (0/71). In contrast, GFP+ cells were observed at varying frequencies using other reprogramming protocols. At 3 DIV, the highest rates were observed with TL2i (57%, n = 490), 4i/L/b (41%, n = 228), t2iLGöY (37%, n = 168), LCDM (26%, n = 335), and TL-CDK8/19i (20%, n = 260). TL2i was the only reprogramming method that returned more GFP+ cells at 2 DIV (58%, n = 145) and 3 DIV (57%, n = 204) compared with 1 DIV (44%, n = 141). These results suggested that TL2i cells have a slightly increased proliferative capacity compared with cells reprogrammed using other methods (Figure 3B).

We subsequently characterized the surviving rhesus PSCs. The chimeric embryos were immunostained with GFP, OCT4, SOX2, NANOG, and GATA6 antibodies at E3 (1 DIV, 335 embryos with GFP+ cells, n = 650), E4 (2 DIV, 202 embryos with GFP+ cells, n = 664), and E5 (3 DIV, 217 embryos with GFP+ cells, n = 932). GFP+/OCT4+ and GFP+/GATA6+ co-immunostaining was frequently observed in chimeric embryos at 3 DIV. In contrast, GFP+/SOX2+ cells were rarely observed (Figure 3C; Table S1). GFP+/SOX2+, GFP+/NANOG+, and GFP+/GATA6+ cells were counted in 754 positive embryos. A strong decrease in the rate of GFP+/SOX2+ and GFP+/NANOG+ cells and an increase in the rate of GFP+/GATA6+ cells were observed between 1 and 3 DIV with all reprogramming protocols (Figure 3D). These results strongly suggest that the vast majority of rhesus PSCs abolished the expression of pluripotency markers and committed prematurely to differentiation after injection into rabbit embryos. A similar result was observed when rabbit embryos injected with TL2i rhesus PSCs were cultured for 3 days in gradual medium supplemented with 250 nM tamoxifen (Figure S4A), indicating that the maintenance of high STAT3 activity is not sufficient to overcome the spontaneous differentiation that occurs during embryo colonization.

In previous experiments, we compared the colonization capabilities of mESCs and monkey PSCs using the same experimental paradigm, which included single-cell dissociation and embryo culture in 20% $O_2$ concentration. Rabbit embryos are routinely cultured at atmospheric $O_2$ concentration (Tapponnier et al., 2017). Thus, we compared the GFP+ cell colonization rates after culturing host embryos under normoxic (20% $O_2$) and hypoxic (5% $O_2$) conditions. Following injection of 10 rhesus TL2i cells, host embryos were cultured under conditions of 5% and 20% $O_2$ concentration (number of embryos treated: 112 and 109, respectively). The rate of embryos containing GFP+ cells was lower at 1, 2, and 3 DIV after culture at 5% $O_2$ concentration (Figure S4B). We also evaluated whether microinjection of cell clumps into rabbit embryos would improve the colonization capabilities compared with single-cell suspensions. The experiment was performed using rhesus TL2i cells. At both 1 and 2 DIV, the rate of GFP+ embryos obtained after injection of small aggregates of 10 cells was lower than that obtained after injection of the same number of isolated cells (1 DIV: 37% versus 53%, respectively; n = 76; 2 DIV: 19% versus 43%, respectively; n = 76; Figure S4C). In contrast, at 3 DIV, injection of aggregates of 10 cells yielded slightly better results than those recorded after injection of the same number of isolated cells (18.9% versus 6.25% of GFP+ embryos, respectively; n = 98). However, we did not observe an increase in the number of GFP+ cells in the positive embryos. Finally, we evaluated the efficacy of colonization after microinjection of ten cells into embryos at the morula stage (E2) compared with the blastocyst stage (E4) (Figure S4D). At 3 DIV, we observed lower levels of GFP+ embryos for TL2i cells (39% versus 6%) and 4i/L/b cells (29% versus 0%) after microinjection into blastocyst-stage embryos. On the basis of these additional experiments, we concluded that injection of embryos at the blastocyst stage, injection of cell clumps, and reduction of $O_2$ concentration during embryo culture had no measurable effects on the rate of embryo colonization by rhesus PSCs.

Previous studies have shown that inhibiting apoptosis either by the addition of the ROCK inhibitor Y27632 (Kang et al., 2018), or by overexpression of the anti-apoptotic genes *BCL2* (Masaki et al., 2016) or BMI1 (Huang et al., 2018), enables primed PSCs to colonize host embryos. Therefore, in a final series of microinjections, we asked whether inhibition of apoptosis could improve the engraftment of naive PSCs into the host embryo. For

**Figure 3. Colonization of Rabbit Embryos by Rhesus Naive PSCs**

(A) Percentage of rabbit embryos with GFP+ cells, 1–3 days (1–3 DIV) after injection of rhesus LyonES-tGFP-(S3) cells into morula-stage (E2) embryos, before (primed) and after reprogramming to the naive state (E-NHSM, NHSM-v, 4i/L/b, TL2i, t2iLGöY, LCDM (EPS), and TL-CDK8/19i) (n = 650 at 1 DIV; n = 664 at 2 DIV; n = 932 at 3 DIV).

(B) Percentage of rabbit embryos with 0, 1, 2, 3, or >3 GFP+ cells 1–3 days (1–3 DIV) after injection of rhesus LyonES-tGFP-(S3) cells into morula-stage (E2) embryos (n = 650 at 1 DIV; n = 664 at 2 DIV; n = 932 at 3 DIV).

(C) Immunostaining of GFP, OCT4, SOX2, and GATA6 in late blastocyst-stage (E5) rabbit embryos after microinjection of rhesus PSCs (confocal imaging). Scale bars, 50 μm.

(D) Percentage of GFP+/NANOG+, GFP+/SOX2+, and GFP+/GATA6+ cells in rabbit embryos 1–3 days after injection of rhesus PSCs (n = 232).

this purpose, NHSM-v and t2iLGöY PSCs were treated with the ROCK inhibitor during dissociation and microinjection into the host embryos. The rates of embryos containing GFP$^+$ cells were similar at all three time points analyzed (1, 2, and 3 DIV; Figure S4E). Thus, addition of ROCK inhibitor did not improve the rate of embryo colonization by rhesus naive PSCs.

### Colonization of Rabbit Pre-implantation Embryos by Human iPSCs

We subsequently investigated whether human naive PSCs exhibited a similar behavior to that of rhesus naive PSCs after injection into rabbit embryos. We tested the colonization capabilities of two human iPSC lines, cR-NCRM2 (Guo et al., 2017) and IR1.7 (Ng et al., 2012), after reprogramming with t2iLGöY (Guo et al., 2017) and TL2i protocols (Chen et al., 2015a), respectively. The cR-NCRM2-t2iLGöY cells express the naive pluripotency markers TFAP2C, KLF4, KLF17, transcription factor CP2-like 1 (TFCP2L1), and sushi domain containing 2 (SUSD2) (Bredenkamp et al., 2019) (Figure S4F). After injection into rabbit morulas, immunofluorescence was used for the detection of human nuclear antigen (HuN) to trace human cells in rabbit host embryos. On days 1, 2, and 3, 93% (n = 29), 65% (n = 31), and 33% (n = 130) of the embryos contained HuN$^+$ cells in the ICM/epiblast, respectively (Figure 4C; Table S1). At 3 DIV, double immunofluorescence analysis revealed that 45% (n = 12), 17% (n = 13), and 32% (n = 6) of the embryos contained HuN$^+$/SOX2$^+$, HuN$^+$/NANOG$^+$, and HuN$^+$/GATA6$^+$ cells, respectively (Figures 4A and 4D). These rates are similar to those obtained previously using t2iLGöY rhesus PSCs (Figure 3B), suggesting extremely similar colonization capabilities for rhesus and human t2iLGöY cells. Chimeric embryos also contained HuN$^+$/SOX17$^+$ (50%, n = 30) and HuN$^+$/T-BRA$^+$ cells (36%, n = 30), but none of the embryos examined contained HuN$^+$/NESTIN$^+$ cells (n = 30), suggesting preferential differentiation toward mesodermal and endodermal lineages (Figures 4A and 4D). Similar results were obtained using naive IR1.7-TL2i cells. These cells have been described previously (Chen et al., 2015a) (Figure S4F). On days 1, 2, and 3, 85% (n = 39), 50% (n = 36), and 28% (n = 39) of embryos, respectively, contained HuN$^+$ cells in the ICM/epiblast (Figure 4C and Table S1). At 3 DIV, chimeric embryos contained HuN$^+$/SOX2$^+$ (10%, n = 76), HuN$^+$/GATA6$^+$ (36%, n = 70), and HuN$^+$/SOX17$^+$ (86%, n = 30) cells (Figures 4B and 4D). None of the embryos examined contained HuN$^+$/NANOG$^+$ (n = 26), HuN$^+$/T-BRA$^+$ (n = 27), and HuN$^+$/NESTIN$^+$ (n = 30) cells. Considering all of the data obtained using both cR-NCRM2-t2iLGöY and IR1.7-TL2i cells, donor cells that colonized rabbit embryos expressed SOX2 (21%, n = 30), NANOG (8%, n = 5), GATA6 (32%, n = 33), SOX17 (58%, n = 35), and T-BRA (19%, n = 11) (Figure 4E). These results illustrated both a low rate of colonization by SOX2$^+$/NANOG$^+$/OCT4$^+$ human naive PSCs at 3 DIV and premature differentiation of the donor cells to endodermal and mesodermal fates, thus confirming and extending the results obtained using rhesus naive PSCs.

We then asked whether the inhibition of apoptosis via overexpression of the anti-apoptotic gene *BCL2* (Masaki et al., 2016) could improve the engraftment of human naive iPSCs and prevent their premature differentiation. Both cR-NCRM2-t2iLGöY and IR1.7-TL2i cells were transfected with doxycycline (Dox)-inducible human BCL2. Stable transfectants expressing BCL2 in response to Dox were selected in neomycin (hereafter called cR-NCRM2-t2iLGöY-BCL2*i* and IR1.7-TL2i-BCL2*i* cells; Figure S4G) and subsequently injected into rabbit morulas before culture for 3 DIV in Dox- or vehicle (control)-supplemented gradual medium. In Dox-supplemented medium, 100% (n = 82) and 81% (n = 76) of the embryos injected with cR-NCRM2-t2iLGöY-BCL2*i* and IR1.7-TL2i-BCL2*i* cells, respectively, exhibited the presence of HuN$^+$ cells in the ICM/epiblast stage, whereas these rates were only 30% (n = 66, $p < 0.001$, $\chi^2$ test) and 34% (n = 77, $p < 0.001$, $\chi^2$ test), respectively, for control cells (Figure 4F and Table S1). Moreover, at 3 DIV, the Dox-induced embryos contained more HuN$^+$ cells than the control embryos (−Dox: 3.3 ± 1.55 cells/embryo; +Dox: 18.7 ± 5.5 cells/embryo; $p < 0.001$, Student's t test; Figures 4H and 4I). These results strongly suggest that BCL2 overexpression prevents donor cell death after embryo injection. It should be noted that the percentage of EdU$^+$ cells was not altered (−Dox: 16% [n = 20]; +Dox: 13% [n = 20]; $p < 0.69$, $\chi^2$ test; Figures 4G and S4H), indicating that BCL2 overexpression had no measurable effect on the mitotic index of the donor cells. The identity of the surviving HuN$^+$ cells was subsequently analyzed via double immunofluorescence at 3 DIV. In the +Dox condition, most embryos contained HuN$^+$/GATA6$^+$ cells, suggesting that BCL2 overexpression does not alter the natural propensity of donor cells to differentiate after injection into host embryos (Figures 4G–4I). To eliminate the confounding effect of the higher HuN$^+$ cell count observed in Dox-treated embryos, we counted the number of HuN$^+$ cells expressing SOX2, NANOG, and GATA6 in both induced (+Dox; n = 144) and uninduced embryos (−Dox; n = 143). The proportions of HuN$^+$/GATA6$^+$ cells were strongly diminished after BCL2 induction (2% [n = 27] versus 31% [n = 20] for cR-NCRM2-t2iLGöY-BCL2*i* cells, $p < 0.001$, $\chi^2$ test; 17% [n = 25] versus 57% [n = 25] for IR1.7-TL2i-BCL2*i* cells, $p < 0.001$, $\chi^2$ test). However, the sharp decrease in the proportion of HuN$^+$/GATA6$^+$ cells was not associated with mirror increases in the proportions of HuN$^+$/NANOG$^+$ (19.5% [n = 27] versus 20% [n = 26] for cR-NCRM2-t2iLGöY-BCL2*i* cells; 0% [n = 52] for IR1.7-TL2i-BCL2*i* cells in

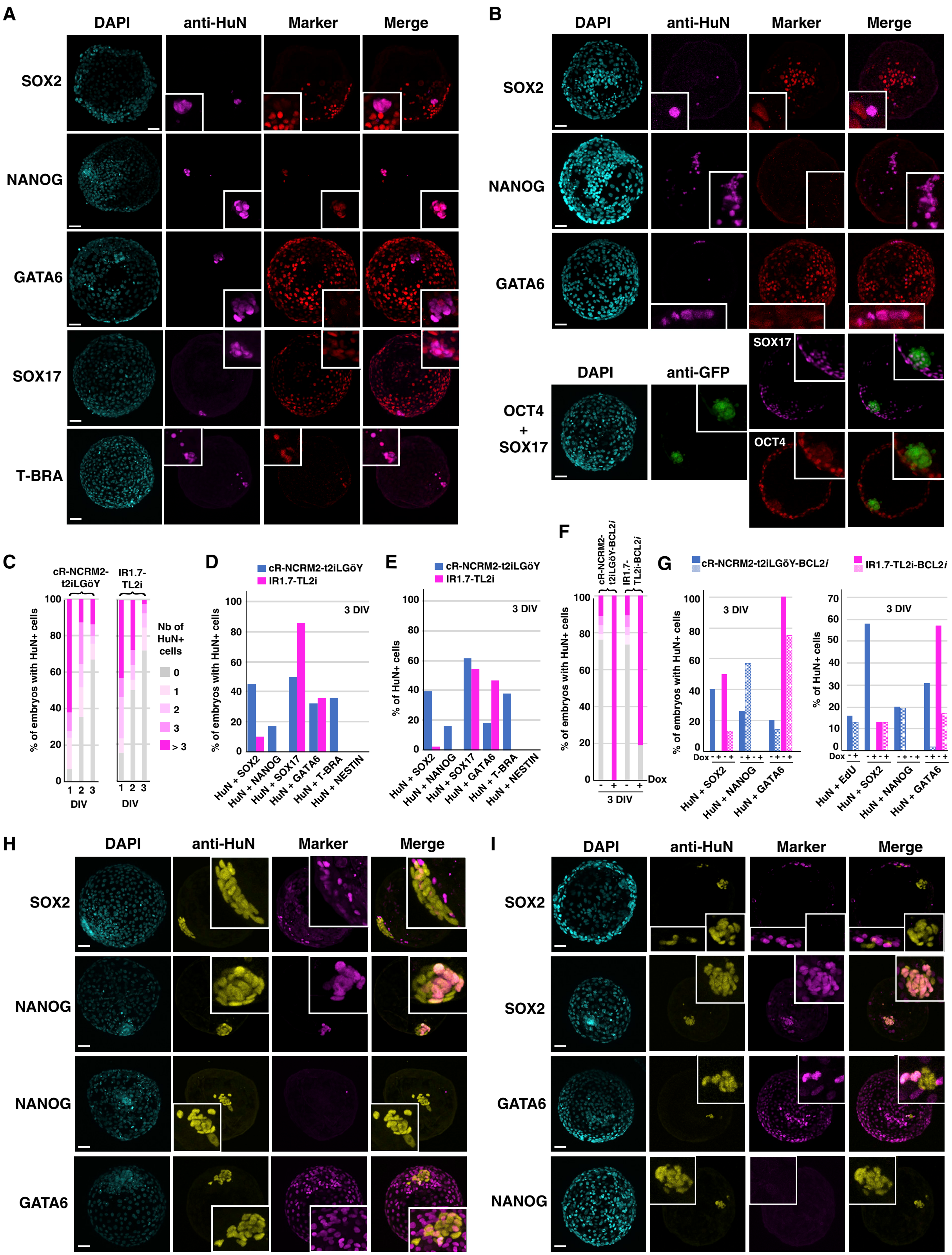


*(legend on next page)*

both + Dox and −Dox conditions) and HuN$^+$/SOX2$^+$ cells (13% [n = 25] in both + Dox and −Dox conditions). On the contrary, it was strongly diminished in cR-NCRM2-t2iLGöY-BCL2*i* cells (0% [n = 28] versus 58% [n = 20], $p < 0.001$, $\chi^2$ test). Thus, BCL2 overexpression significantly increased the efficiency of embryo colonization by human naive PSCs. However, in most of the examined embryos, the vast majority of the donor cells failed to maintain the expression of pluripotency markers, and many of them remained unspecified or expressed GATA6.

### Colonization of Cynomolgus Monkey Pre-implantation Embryos by Rhesus PSCs and Human iPSCs

We questioned whether the low rate of proliferation and ICM/epiblast colonization of human TL2i and t2iLGöY cells after injection into rabbit morulas were due to the evolutionary distance between the two species. To answer this question, we injected human cR-NCRM2-t2iLGöY and IR1.7-TL2i cells, and rhesus TL2i cells, into cynomolgus monkey embryos. Ten cells of each were injected into embryos at the morula stage (E4). The embryos were subsequently cultured for 3 DIV in gradual medium and developed into mid/late (E7) blastocysts before being subjected to triple immunostaining for GFP, OCT4, and NANOG. Of the 29 cynomolgus embryos, 13% (n = 15) had incorporated human cR-NCRM2-t2iLGöY cells, for a total of 5 GFP$^+$ cells (2 and 3 GFP$^+$ cells/embryo), of which only 1 was both OCT4$^+$ and NANOG$^+$ (Figure 5A; Table S1); 29% (n = 7) of the cynomolgus embryos had incorporated human IR1.7-TL2i cells, for a total of 14 GFP$^+$ cells (10 and 4 GFP$^+$ cells/embryo), of which 2 were either OCT4$^+$ or NANOG$^+$ (Figure 5B; Table S1); and 29% (n = 7) of cynomolgus embryos had incorporated rhesus TL2i cells, for a total of 3 GFP$^+$ cells (1 and 2 GFP$^+$ cells/embryo), none of which were either OCT4$^+$ or NANOG$^+$ (Figure 5C; Table S1). These results indicate that human iPSCs (IR1.7-TL2i and cR-NCRM2-t2iLGöY) and rhesus monkey PSCs (LyonES-tGFP-(S3)-TL2i) show similar behaviors after injection into closely and distantly related embryos (cynomolgus and rabbit embryos, respectively).

### Contrasting Cell-Cycle Parameters of mESCs, and Rhesus and Human Naive PSCs

To understand why rhesus and human PSCs stop multiplying when injected into host embryos, we examined both the DNA replication and cell-cycle distribution. We started by examining the incorporation of EdU as a measure of the fraction of cells that replicate in the cell population (% EdU$^+$). Measurements were performed in adherent cells (“Adh”), after single-cell dissociation with trypsin (“0h”), and after re-incubation for 1 h (“1h”) and 2 h (“2h,” human cells only) at 37°C in order to mimic the conditions encountered by the cells during injection into host embryos. mESCs incorporated EdU very rapidly (Figure 6A), consistent with previous results describing the high rate of 5-bromo-2-deoxyuridine incorporation in mESCs (Savatier et al., 1996). Single-cell dissociation did not alter the percentage of EdU$^+$ mESCs (77% and 78% of EdU$^+$ cells in Adh and 0h cells, respectively). It slightly decreased in 1h cells (48% of EdU$^+$ cells); however, the rate of EdU incorporation remained unchanged. These observations suggest that DNA replication is not altered by the experimental setting in the vast majority of mESCs. Similar conclusions were made with mESCs cultured in 2i/LIF medium. However, it should be noted

**Figure 4. Colonization of Rabbit Embryos by Human Naive PSCs**

(A) Immunostaining of HuN, SOX2, NANOG, GATA6, SOX17, and T-BRA in late blastocyst-stage rabbit embryos (E5, 3 DIV) after microinjection of cR-NCRM2-t2iLGöY cells into morula-stage (E2) embryos (confocal imaging). Scale bars, 50 μm.

(B) Immunostaining of HuN, SOX2, NANOG, GATA6, OCT4, and SOX17 in late blastocyst-stage rabbit embryos (E5, 3 DIV) after microinjection of IR7.1-TL2i cells into morula-stage (E2) embryos (confocal imaging). Scale bars, 50 μm.

(C) Percentage of rabbit embryos with 0, 1, 2, 3, or >3 HuN$^+$ cells at 1–3 days (1–3 DIV) after injection of cR-NCRM2-t2iLGöY (n = 191) and IR7.1-TL2i cells (n = 114).

(D) Percentage of rabbit embryos with HuN$^+$/SOX2$^+$, HuN$^+$/NANOG$^+$, HuN$^+$/GATA6$^+$, HuN$^+$/SOX17$^+$, HuN$^+$/T-BRA$^+$, and HuN$^+$/NESTIN$^+$ cells at 3 DIV after injection of cR-NCRM2-t2iLGöY and IR7.1-TL2i cells.

(E) Percentage of HuN$^+$/SOX2$^+$, HuN$^+$/NANOG$^+$, HuN$^+$/OCT4$^+$, HuN$^+$/GATA6$^+$, HuN$^+$/SOX17$^+$, HuN$^+$/T-BRA$^+$, and HuN$^+$/NESTIN$^+$ cells at 3 DIV after injection of cR-NCRM2-t2iLGöY and IR7.1-TL2i cells.

(F) Percentage of rabbit embryos with 0, 1, 2, 3, or >3 HuN$^+$ cells at 3 days (3 DIV) after injection of cR-NCRM2-t2iLGöY-BCL2*i* (+/− Dox) and IR7.1-TL2i-BCL2*i* cells (+/− Dox).

(G) Left panel: percentage of rabbit embryos with HuN$^+$/SOX2$^+$, HuN$^+$/NANOG$^+$, and HuN$^+$/GATA6$^+$ cells at 3 DIV after injection of cR-NCRM2-t2iLGöY-BCL2*i* and IR7.1-TL2i-BCL2*i* cells (+/− Dox); right panel: percentage of HuN$^+$/EdU$^+$, HuN$^+$/SOX2$^+$, HuN$^+$/NANOG$^+$, and HuN$^+$/GATA6$^+$ cells at 3 DIV after injection of cR-NCRM2-t2iLGöY-BCL2*i* and IR7.1-TL2i-BCL2*i* cells (+/− Dox).

(H) Immunostaining of HuN, SOX2, NANOG, and GATA6 in late blastocyst-stage rabbit embryos (E5, 3 DIV) after microinjection of cR-NCRM2-t2iLGöY-BCL2*i* cells into morula-stage (E2) embryos (confocal imaging). Scale bars, 50 μm.

(I) Immunostaining of HuN, SOX2, NANOG, and GATA6 in late blastocyst-stage rabbit embryos (E5, 3 DIV) after microinjection of IR7.1-TL2i-BCL2*i* cells into morula-stage (E2) embryos (confocal imaging). Scale bars, 50 μm.

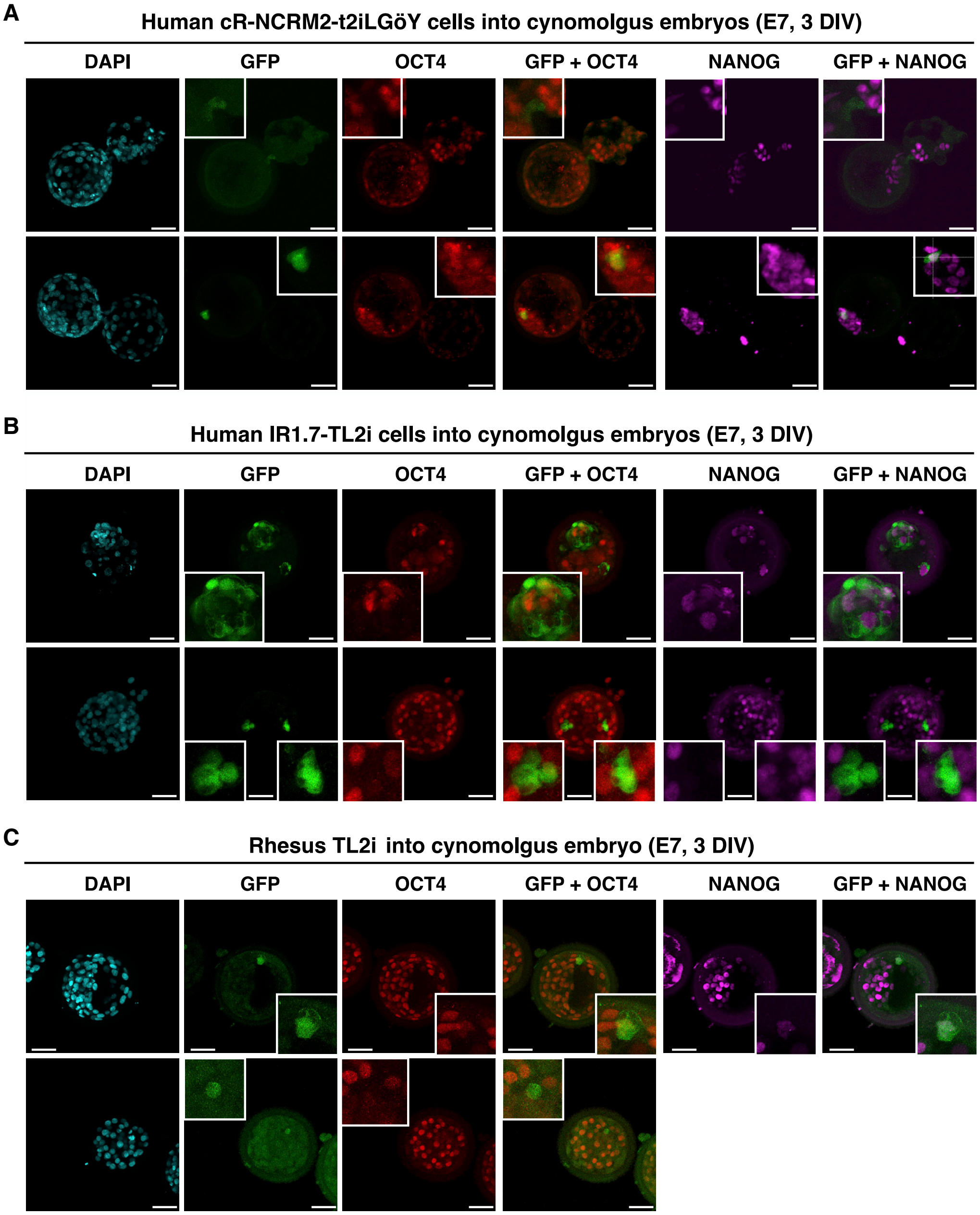


**Figure 5. Colonization of Cynomolgus Monkey Embryos by Human iPSCs and Rhesus PSCs**
(A) Immunolabeling of GFP, OCT4, and NANOG in late blastocyst-stage cynomolgus embryos (E7) after microinjection of human cR-NCRM2-t2iLGöY cells.
(B) Immunolabeling of GFP, OCT4, and NANOG in late blastocyst-stage cynomolgus embryos (E7) after microinjection of human IR7.1-TL2i cells.
(C) Immunolabeling of GFP, OCT4, and NANOG in late blastocyst-stage cynomolgus embryos (E7) after microinjection of rhesus TL2i cells.
(A–C) Confocal imaging. Scale bars, 50 μm.

that the rate of EdU incorporation was lower under 2i/LIF culture conditions compared with that under serum/LIF conditions. This finding is consistent with those of a previous report showing that mESCs cultured in 2i/LIF exhibit a slower cell cycle compared with their serum/LIF counterparts (Ter Huurne et al., 2017).

The pattern of EdU incorporation in primed LyonES-tGFP-(S3) cells was markedly different. The percentage of

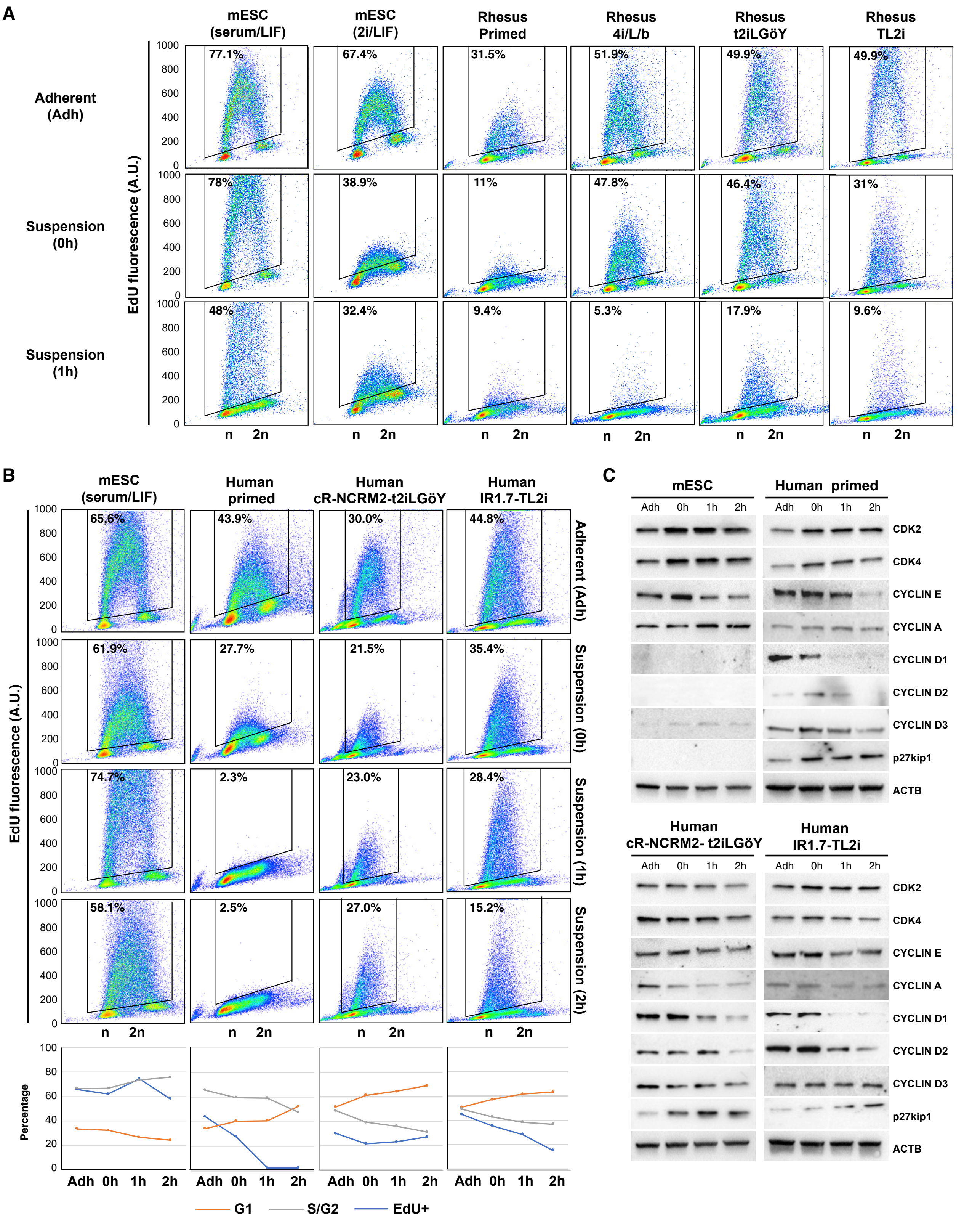


(legend on next page)

EdU+ cells and the incorporation rate were significantly reduced in Adh cells compared with those measured in mESCs (Figure 6A). They were further reduced in 0h and 1h cells. In 1h cells, DNA replication was almost completely abolished. This reveals the inherent failure of primed LyonES-tGFP-(S3) cells to sustain cell-cycle progression following single-cell dissociation. Interestingly, reprogramming primed LyonES-tGFP-(S3) cells with 4i/L/b, TL2i, and t2iLGöY protocols significantly increased the percentage of EdU+ cells, consistent with a previous report indicating that resetting naive-like features accelerated the cell cycle of human PSCs (Chen et al., 2015a). However, unlike mESCs, rhesus naive 4i/L/b, TL2i, and t2iLGöY cells slowed DNA replication after dissociation, demonstrated by a significant reduction of EdU incorporation in both 0h and 1h cells. In 1h cells, only 6%, 10%, and 18% of the 4i/L/b, TL2i, and t2iLGöY cells, respectively, incorporated EdU (compared with 48% of mESCs under the same experimental conditions). Furthermore, the incorporation rate was considerably reduced as compared with Adh 4i/L/b, TL2i, and t2iLGöY cells and 1h control mESCs. Overall, these results indicate that rhesus naive PSCs, unlike mESCs, fail to maintain active DNA replication in this experimental setting. In addition, the cell-cycle distribution of the 1h samples showed a slight increase in the proportion of cells in the G1 phase at the expense of cells in the G2 phase in rhesus primed, 4i/L/b, TL2i, and t2iLGöY cells, suggesting that some cells have already undergone growth arrest in G1 phase. This alteration was not observed in mESCs (Figure S5A). Taken together, these results indicate that the vast majority of naive rhesus cells are markedly slowed in their mitotic cycle at the time they are injected into host embryos, or shortly after.

Similar results were obtained using human IR1.7-TL2i and cR-NCRM2-t2iLGöY cells in the Adh, 0h, and 1h conditions. A 2h condition (i.e., culture in suspension for 2 h) was added to the experimental scheme. The percentage of EdU+ cells was reduced under the 0h, 1h, and 2h cells compared with the findings in Adh cells (Figure 6B). In 2h cells, only 15.2% of the IR1.7-TL2i cells incorporated EdU (compared with 43.8% in Adh IR1.7-TL2i cells, 2.5% in primed cells, and 58.1% in 2h mESCs). The impact of suspension culture was less pronounced in human cR-NCRM2-t2iLGöY cells. However, the rate of EdU incorporation was significantly lower in cR-NCRM2-t2iLGöY cells than in IR1.7-TL2i cells. Interestingly, human cR-NCRM2-t2iLGöY cells exhibited a unique pattern of EdU incorporation, in which the percentage of EdU+ cells was higher at 2h (27%) than at 0h (21.5%). This observation suggests that some cells in the human cR-NCRM2-t2iLGöY population may have the capacity to recover from the deleterious effects of single-cell dissociation and gradually re-engage in the mitotic cycle. This phenomenon was not observed in any other cell lines. We also analyzed the cell-cycle distribution (Figures 6B and S5B). In all human cells (primed, IR1.7-TL2i and cR-NCRM2-t2iLGöY), the cell-cycle distribution of the 2h samples exhibited an increased proportion of cells in G1 phase at the expense of cells in S and G2 phases, suggesting that some cells have already undergone growth arrest in G1 phase. This alteration was not observed in mESCs, further indicating that only mESCs retain the capacity to actively divide in non-adherent culture conditions.

To understand why the mitotic cycle is slowed in human naive PSCs after single-cell dissociation, we studied the expression of cell-cycle regulators, including CDK2, CDK4, CYCLIN D1, D2, D3, E, and A, and the CDK inhibitor $p27^{Kip1}$ (Figures 6C and S5C). No differences of CDK2 and CDK4 levels were observed among Adh, 0h, 1h, and 2h samples in all cell types analyzed. In mESCs, the levels of CYCLIN E and A were increased. CYCLIN D1, CYCLIN D2, and $p27^{Kip1}$ were not expressed as reported previously (Savatier et al., 1996) (Stead et al., 2002). In human primed PSCs, the expression of CYCLIN D1, D2, and D3 was decreased and that of $p27^{Kip1}$ was increased after dissociation and suspension culture. In cR-NCRM2-t2iLGöY cells, the expression of CYCLIN D1, D2, D3, and A was decreased, and that of $p27^{Kip1}$ was increased. In IR1.7-TL2i cells, the expression of CYCLIN D2 and A was decreased, and that of $p27^{Kip1}$ was increased. Therefore, single-cell dissociation followed by suspension culture triggers opposite responses in mESCs and human naive PSCs. In mESCs, it activates the expression of positive regulators of the mitotic cycle, whereas in human naive PSCs it reduces their levels, and concomitantly increases that of a negative regulator. These results are consistent with the results of EdU incorporation and

**Figure 6. Cell-Cycle Parameters of Mouse ESCs, Rhesus PSCs, and Human iPSCs, before and after Reprogramming to Naive States**

(A) Flow cytometry analysis of EdU incorporation and propidium iodide (PI) staining of mESCs (serum/LIF and 2i/LIF) and rhesus PSCs (primed, 4i/L/b, TL2i, and t2iLGöY) in Adh, 0h, and 1h conditions. Numbers indicate percentages of EdU+ cells.

(B) Flow cytometry analysis of EdU incorporation and PI staining of mESCs (serum/LIF) and human iPSCs (primed, cR-NCRM2-t2iLGöY and IR7.1-TL2i) in Adh, 0h, 1h, and 2h conditions. Numbers indicate percentages of EdU+ cells. Histograms show the cell-cycle distribution (% EdU+, G1, and S/G2 cells).

(C) Western blot analysis of cell-cycle regulators in mESCs (serum/LIF) and human iPSCs (primed, cR-NCRM2-t2iLGöY and IR1.7-TL2i) in Adh, 0h, 1h, and 2h conditions (results of three experiments).

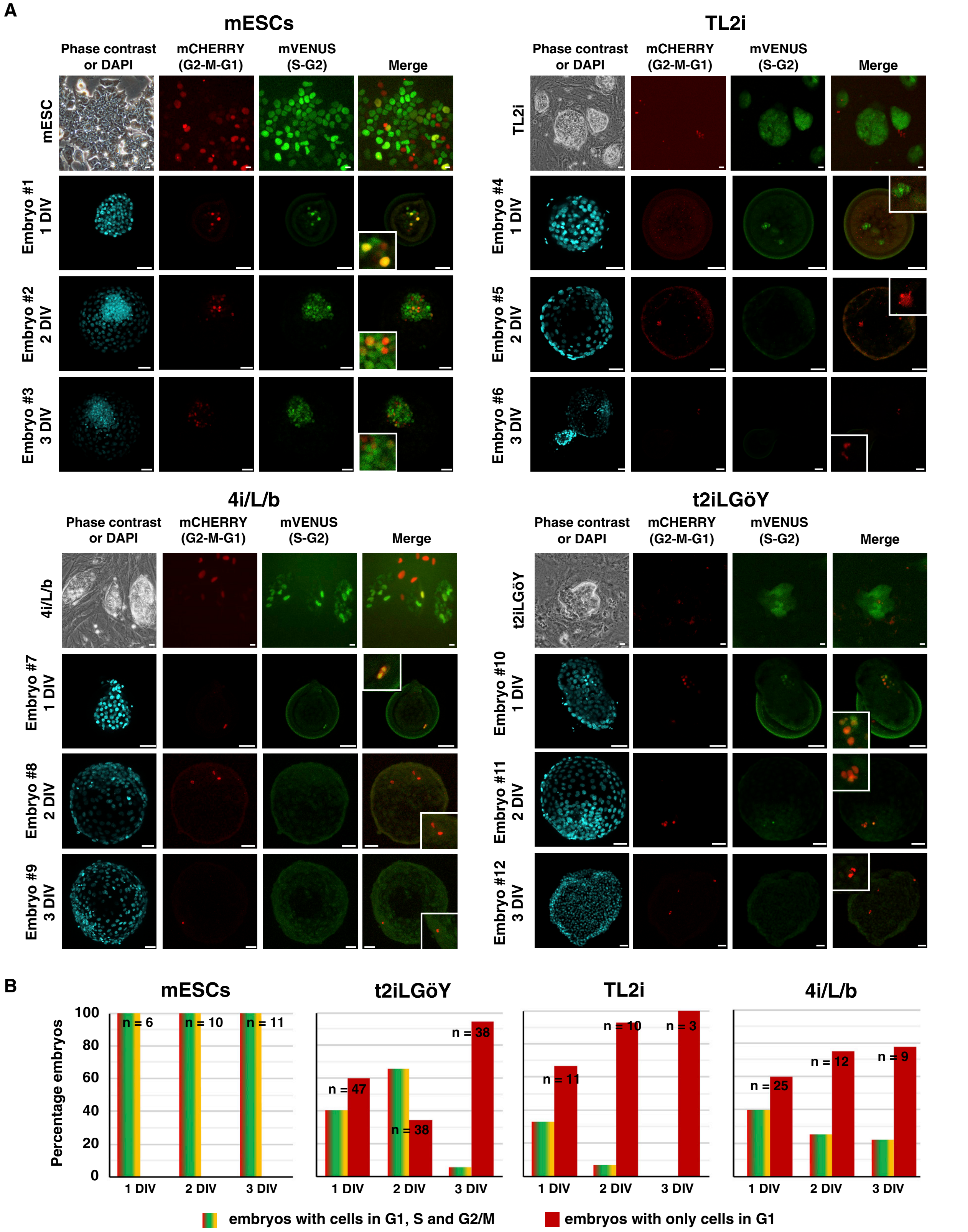


*(legend on next page)*

cytometry analyses, revealing cell-cycle delay, and possibly growth arrest in G1 phase.

### Rhesus PSCs Stall in the G1 Phase after Transfer into Rabbit Embryos

We observed that mESCs continue to actively replicate their DNA after injection into rabbit embryos (Figure 1C). Therefore, we investigated whether naive rhesus cells that survive injection and successfully colonize the ICM/epiblast retain a high proliferative capacity. For this purpose, ten rhesus TL2i cells were injected into rabbit embryos at the morula stage. The embryos were subsequently cultured for 1 and 2 DIV before pulse labeling with EdU. At 1 DIV, seven embryos had GFP$^+$ cells in the ICM/epiblast (37%, n = 19) for a total of 29 GFP$^+$ cells. Triple immunostaining for GFP, SOX2, and EdU showed that 24 (82%) of these 29 GFP$^+$ cells expressed SOX2, of which only four had incorporated EdU (Figure S6). At 2 DIV, nine embryos had GFP$^+$ cells in the ICM/epiblast (60%, n = 15) for a total of 61 GFP$^+$ cells. Of these 61 GFP$^+$ cells, 44 (72%) expressed SOX2, and 1 incorporated EdU (2%). These results reveal that the rhesus naive cells that survived and colonized the rabbit embryos retarded their DNA replication and potentially underwent growth arrest.

To obtain further insight into the cell cycle of the mESCs and rhesus 4i/L/b, TL2i, and t2iLGöY naive PSCs after injection into rabbit embryos, we generated cell lines expressing the FUCCI(CA) reporter system (Sakaue-Sawano et al., 2017). The mESCs expressing *PB-Puro$^R$- CAG-mVenus:hGeminin-IRES-mCherry:hCdt1*, hereinafter referred to as mESC-FUCCI(CA), exhibited a cell-cycle distribution typical of mESCs (18%, 69%, and 11% of cells in G1, S, and G2 phases, respectively; Figures S7A and S7B). On the other hand, rhesus PSC-FUCCI(CA) cells exhibited an extended G1 phase relative to other cell-cycle phases (43%, 36%, and 13% of the cells in G1, S, and G2 phases, respectively) (Figures S7C and S7D). After injection into rabbit embryos, mESC-FUCCI(CA) cells proliferated actively during the 3 DIV, as shown by the expansion of the mVENUS$^+$ cell population identified through immunostaining with an anti-mVENUS antibody (green) in 100% of the embryos (Figures 7A and 7B). Even after 3 DIV, mCHERRY$^+$ cells (red), identified using an anti-mCHERRY antibody, and mVENUS$^+$/mCHERRY$^+$ cells (yellow), were proportionally less numerous, indicating that only rare mESCs were in G1 and G2 phases, respectively. In addition, at all time points analyzed, there were no embryos containing only mCHERRY$^+$ (red) cells (n = 27) (Figure 7B). These results indicate that mESCs continue to proliferate actively for at least 3 days after injection into host embryos, in line with the EdU incorporation data.

On the other hand, the cell-cycle distribution of rhesus PSC-FUCCI(CA) cells after injection into rabbit embryos was clearly different, regardless of the method used (4i/L/b, TL2i, and t2iLGöY) for reprogramming to the naive state. At 1 DIV, the majority of embryos contained only mCHERRY$^+$ (red) cells (t2iLGöY, 60%, n = 47; TL2i, 68%, n = 11; 4i/L/b, 60%, n = 25). The remaining embryos contained both mVENUS$^+$ (green) and mVENUS$^+$/mCHERRY$^+$ (yellow) cells in addition to mCHERRY$^+$ (red) cells. In embryos injected with TL2i and 4i/L/b cells, the rate of embryos containing only mCHERRY$^+$ (red) cells increased over time in culture, reaching 78% (n = 9) and 100% (n = 3) with 4i/L/b and TL2i cells, respectively, at 3 DIV (Figure 7B). In embryos injected with t2iLGöY cells, the proportion of embryos containing only mCHERRY$^+$ (red) cells first decreased at 2 DIV (35%, n = 36) and subsequently increased at 3 DIV (95%, n = 35) (Figure 7B). These results indicate that most naive rhesus PSC-FUCCI(CA) cells accumulate in the G1 phase of the cell cycle after injection into rabbit embryos.

## DISCUSSION

In our study, we aimed to understand why human naive PSCs failed to colonize mouse and pig embryos (Masaki et al., 2015; Theunissen et al., 2016; Wu et al., 2017), despite exhibiting characteristic features of naive pluripotency as primarily defined in rodents. Our main conclusion is that human and NHP naive PSCs are inherently unfit to remain mitotically active during embryo colonization and therefore differentiate prematurely.

The first question we addressed was whether or not host embryos are permissive to colonization by naive PSCs. To answer this question, we injected mouse ESCs into rabbit and cynomolgus embryos and showed that they continue to express pluripotency markers, they remain mitotically active for at least 3 DIV, and they contributed to the expansion of the host epiblast. Some of them contributed to the formation of the neural tube in rabbit post-implantation embryos. We conclude that the ICM and epiblast of rabbit and cynomolgus embryos are permissive to colonization

**Figure 7. Cell-Cycle Distribution of Mouse and Rhesus PSCs after Injection into Rabbit Embryos**

(A) Phase contrast and epifluorescence imaging of mouse ESCs and rhesus TL2i, 4i/L/b, and t2iLGöY PSCs expressing the FUCCI(CA) cell-cycle reporter system. Immunostaining of mVenus and mCherry in rabbit embryos 1–3 days (1–3 DIV) after microinjection of FUCCI(CA) mouse and rhesus PSCs into morula-stage (E2) embryos (confocal imaging). Scale bars, 50 μm.

(B) Histogram of the percentage of rabbit embryos having cells in G1, S, and G2 phases of the cell cycle at 1, 2, and 3 DIV (n = 220).

by bona fide naive PSCs. These results may seem to contradict those of Wu et al. (2017), who injected mouse ESCs into pig embryos and observed no chimerism at days 21–28 of gestation. Differentiated cells derived from mouse ESCs are likely to disappear from the chimera through cell competition in later stages of development; however, this should not obscure the fact that the mouse ESCs are initially capable of colonizing pre-implantation embryos very efficiently.

In sharp contrast with mESCs, none of the naive rhesus and human PSCs tested in the same experimental paradigm is capable of massively colonizing the ICM and epiblast of the host, whether rabbit or cynomolgus monkey embryos. Some cells retaining the expression of pluripotency markers were observed at 3 DIV in rabbit embryos after injection of LCDM (EPS) (9% of total embryos), 4i/L/b (2% of total embryos), TL2i (5% of total embryos), TL-CDK8/19i (2% of total embryos), and t2iLGöY PSCs (3% of total embryos) (Table S1). In these 234 chimeric embryos, the total number of $GFP^+$ cells expressing pluripotency markers after 3 DIV was 3.26 ± 1.27, when 10 cells were injected at the morula stage. The other cells differentiated prematurely. In contrast, up to 40 $GFP^+/NANOG^+$ and 36 $GFP^+/SOX2^+$ cells/embryo were observed following injection of 10 mESCs into rabbit morulas after the same period of culture. Similarly, up to 23 $GFP^+/SOX2^+$ cells/embryo were observed after injection of mESC-2i/LIF cells. These results illustrate the striking difference observed between mouse ESCs and primate naive PSCs in the stability of pluripotency gene expression after injection into host embryos. As a result, none of the protocols tested confer an embryo colonization competence to primate naive PSCs similar to that of mESCs. It should be noted, however, that the different protocols tested for reprogramming human and NHP PSCs did not yield identical results. We were unable to generate undeniable chimeric blastocysts with the E-NHSM and NHSM-v protocols. This result is in contradiction with a previous publication reporting efficient colonization of cynomolgus monkey embryos by PSCs reprogrammed using the NHSM-v protocol (Chen et al., 2015b). This difference can be explained by the immunodetection of $GFP^+$ cells in the developing embryos, which eliminates the confounding effect of autofluorescence due to dying and necrotic cells. We did not observe significant differences in the rate of colonization by $GFP^+/NANOG^+$, $GFP^+/OCT4^+$, and $SOX2^+/GFP^+$ cells between the other five reprogramming protocols. We do not rule out the possibility that some of these protocols may be more effective than others in generating cells capable of colonization. However, the number of unquestionable chimeric blastocysts obtained with each of these protocols was insufficient to conclude about significant differences.

After injection into rabbit and macaque monkey morulas, most rhesus and human naive PSCs undergo cell death. We showed that the few cells that survive after 3 DIV stall in the G1 phase and commit to differentiation, demonstrated by the loss of pluripotency markers (OCT4, SOX2, and NANOG) and the activation of differentiation markers (GATA6, SOX17, and T-BRA). Cell death can be prevented by overexpressing BCL2, leading to an apparent increase in the rate of colonization by donor cells. However, the proportion of pluripotent cells remains globally unchanged, indicating that the prevention of cell death does not prevent the premature differentiation observed with *BCL2* transgene expression-negative cells. The behavior of primate naive PSCs is in sharp contrast with that of mESCs, which continue to actively proliferate after injection into host embryos. These results reveal the inability of human and rhesus monkey naive PSCs to remain mitotically active in an unfavorable environment and question their very nature. Despite requiring complex culture media, human and NHP naive PSCs self-renew in a more precarious equilibrium than mESCs. This suggests a much higher sensitivity to alterations of their local environment, possibly explaining the poor performance of human and rhesus monkey naive PSCs in colonizing the epiblast of host embryos. As a matter of fact, disruption of the human and rhesus PSC cell cycle is already demonstrated immediately after the colony dissociation (i.e., before embryo injection) where most naive PSCs cells undergo a drastic slowdown in DNA replication. Mitotic arrest is associated with downregulation of CYCLIN expression and an upregulation of $p27^{Kip1}$ expression. We speculate that only few cells retain the capacity to actively divide and re-enter the next division cycle when introduced into the embryo environment.

In the prospect of generating interspecies chimeras, an equally important issue is the influence of the host species on the rate of colonization. Rhesus and human PSCs (TL2i and t2iLGöY reprogramming protocols) did not show a marked difference in survival rate and stability of pluripotency gene expression after injection into rabbit and cynomolgus embryos. Similarly, mouse ESCs were able to colonize both rabbit and cynomolgus embryos with high efficiency. This suggests that the rabbit embryo is a valuable model system for exploring further improvements.

## EXPERIMENTAL PROCEDURES

### Cell Lines, Media Composition, and Culture

Primed-to-naive conversion was performed using previously described protocols, including E-NHSM (https://hannalabweb.weizmann.ac.il) (Gafni et al., 2013), NHSM-v (Chen et al., 2015b), 4i/L/b (Fang et al., 2014), 5iL/A (Theunissen et al., 2014), t2iLGöY (Guo et al., 2017), TL2i (Chen et al., 2015a), TL-CDK8/

19i (modified from Lynch et al., 2020), and LCDM (EPS) (Yang et al., 2017a). Detailed protocols are provided in the Supplemental Experimental Procedure.

## ETHICS

All procedures in macaque monkeys were approved by the French ethics committee CELYNE (approval no.: APAFIS#4858). All animal procedures in rabbits were approved by the French ethics committee CELYNE (APAFIS#6438). Chimera experiments involving human iPSCs were approved by the INSERM ethics committee.

### RNA Sequencing

RNA from cell lines was extracted and libraries were prepared using 200 ng of RNA with the NextFlex Rapid Directional mRNA-Seq kit (Bioo-Scientific). Samples were sequenced on a NextSeq500 sequencing machine (Illumina) as single reads of 75 bp. Detailed protocols are provided in Supplemental Experimental Procedure.

### Data and Code Availability

The RNA sequencing datasets generated during this study are available under GEO accession number GSE146178.

## SUPPLEMENTAL INFORMATION

Supplemental Information can be found online at https://doi.org/10.1016/j.stemcr.2020.12.004.

## AUTHOR CONTRIBUTIONS

I.A., C.R., A.M., E.M., V.C., A.B.-M., N.D., F.W., P.-Y.B., and M.A. performed cell and embryo cultures and cell characterizations. M.D. and T.J. performed animal surgery. G.M., C.M., and O.R. performed bioinformatic analyses. C.L., M.S., and C.D. shared unpublished expertise. I.A. and P.S. analyzed the data and wrote the manuscript.

## ACKNOWLEDGMENTS

We are grateful to all members of the animal facility team for their work and dedication, and to Charlotte Bréhier for technical help during the revision of the manuscript. We thank Dr. Austin Smith and Dr. Ge Guo for sharing the cR-NCRM2 cell line and the RIKEN BRC DNABank for providing the mCherry-hCdt1(1/100)Cy(−)/pcDNA3 and mVenus-hGeminin(1/110)/pcDNA3 plasmids. This work was supported by the Fondation pour la Recherche Médicale (DEQ20170336757 and ARF20140129246), the Fondation pour la recherche contre le cancer (RAC18005CCA), the Infrastructure Nationale en Biologie et Santé INGESTEM (ANR-11-INBS-0009), the IHU-B CESAME (ANR-10-IBHU-003), the LabEx "REVIVE" (ANR-10-LABX-73), the LabEx “DEVweCAN” (ANR-10-LABX-0061), the LabEx “CORTEX” (ANR-11-LABX-0042), and the University of Lyon within the program “Investissements d’Avenir” (ANR-11-IDEX-0007). Work in the laboratory of M.S. was funded by the IRB and by grants from the Spanish Ministry of Economy co-funded by the European Regional Development Fund (ERDF) (SAF2017-82613-R), the European Research Council (ERC-2014-AdG/669622), and “La Caixa” Foundation.

Received: March 27, 2020
Revised: December 1, 2020
Accepted: December 1, 2020
Published: December 30, 2020

## REFERENCES

Bredenkamp, N., Stirparo, G.G., Nichols, J., Smith, A., and Guo, G. (2019). The cell-surface marker sushi containing domain 2 facilitates establishment of human naive pluripotent stem cells. Stem Cell Reports *12*, 1212–1222.

Chan, Y.S., Göke, J., Ng, J.H., Lu, X., Gonzales, K.A., Tan, C.P., Tng, W.Q., Hong, Z.Z., Lim, Y.S., and Ng, H.H. (2013). Induction of a human pluripotent state with distinct regulatory circuitry that resembles pre-implantation epiblast. Cell Stem Cell *13*, 663–675.

Chen, H., Aksoy, I., Gonnot, F., Osteil, P., Aubry, M., Hamela, C., Rognard, C., Hochard, A., Voisin, S., Fontaine, E., et al. (2015a). Reinforcement of STAT3 activity reprogrammes human embryonic stem cells to naive-like pluripotency. Nat. Commun. *6*, 7095–7112.

Chen, Y., and Lai, D. (2015). Pluripotent states of human embryonic stem cells. Cell Reprogram. *17*, 1–6.

Chen, Y., Niu, Y., Li, Y., Ai, Z., Kang, Y., Shi, H., Xiang, Z., Yang, Z., Tan, T., Si, W., et al. (2015b). Generation of cynomolgus monkey chimeric fetuses using embryonic stem cells. Cell Stem Cell *17*, 116–124.

Davidson, K.C., Mason, E.A., and Pera, M.F. (2015). The pluripotent state in mouse and human. Development *142*, 3090–3099.

Fang, R., Liu, K., Zhao, Y., Li, H., Zhu, D., Du, Y., Xiang, C., Li, X., Liu, H., Miao, Z., et al. (2014). Generation of naive induced pluripotent stem cells from rhesus monkey fibroblasts. Cell Stem Cell *15*, 488–497.

Gafni, O., Weinberger, L., Mansour, A.A., Manor, Y.S., Chomsky, E., Ben-Yosef, D., Kalma, Y., Viukov, S., Maza, I., Zviran, A., et al. (2013). Derivation of novel human ground state naive pluripotent stem cells. Nature *504*, 282–286.

Guo, G., von Meyenn, F., Rostovskaya, M., Clarke, J., Dietmann, S., Baker, D., Sahakyan, A., Myers, S., Bertone, P., Reik, W., et al. (2017). Epigenetic resetting of human pluripotency. Development *144*, 2748–2763.

Guo, G., von Meyenn, F., Santos, F., Chen, Y., Reik, W., Bertone, P., Smith, A., and Nichols, J. (2016). Naive pluripotent stem cells derived directly from isolated cells of the human inner cell mass. Stem Cell Reports *6*, 437–446.

Huang, K., Maruyama, T., and Fan, G. (2014). The naive state of human pluripotent stem cells: a synthesis of stem cell and pre-implantation embryo transcriptome analyses. Cell Stem Cell *15*, 410–415.

Huang, K., Zhu, Y., Ma, Y., Zhao, B., Fan, N., Li, Y., Song, H., Chu, S., Ouyang, Z., Zhang, Q., et al. (2018). BMI1 enables interspecies chimerism with human pluripotent stem cells. Nat. Commun. *9*, 4649.

Kang, Y., Ai, Z., Duan, K., Si, C., Wang, Y., Zheng, Y., He, J., Yin, Y., Zhao, S., Niu, B., et al. (2018). Improving cell survival in injected embryos allows primed pluripotent stem cells to generate chimeric cynomolgus monkeys. Cell Rep. *25*, 2563–e9.

Lynch, C.J., Bernad, R., Martinez-Val, A., Shahbazi, M.N., Nobrega-Pereira, S., Calvo, I., Blanco-Aparicio, C., Tarantino, C., Garreta, E., Richart-Gines, L., et al. (2020). Global hyperactivation of enhancers stabilizes human and mouse naïve pluripotency through inhibition of CDK8/19 mediator kinases. Nat. Cell Biol. *22*, 1223–1238.

Madeja, Z.E., Pawlak, P., and Piliszek, A. (2019). Beyond the mouse: non-rodent animal models for study of early mammalian development and biomedical research. Int. J. Dev. Biol. *63*, 187–201.

Masaki, H., Kato-Itoh, M., Takahashi, Y., Umino, A., Sato, H., Ito, K., Yanagida, A., Nishimura, T., Yamaguchi, T., Hirabayashi, M., et al. (2016). Inhibition of apoptosis overcomes stage-related compatibility barriers to chimera formation in mouse embryos. Cell Stem Cell *19*, 587–592.

Masaki, H., Kato-Itoh, M., Umino, A., Sato, H., Hamanaka, S., Kobayashi, T., Yamaguchi, T., Nishimura, K., Ohtaka, M., Nakanishi, M., and Nakauchi, H. (2015). Interspecific in vitro assay for the chimera-forming ability of human pluripotent stem cells. Development *142*, 3222–3230.

Nakamura, T., Okamoto, I., Sasaki, K., Yabuta, Y., Iwatani, C., Tsuchiya, H., Seita, Y., Nakamura, S., Yamamoto, T., and Saitou, M. (2016). A developmental coordinate of pluripotency among mice, monkeys and humans. Nature *537*, 57–62.

Ng, S.Y., Johnson, R., and Stanton, L.W. (2012). Human long noncoding RNAs promote pluripotency and neuronal differentiation by association with chromatin modifiers and transcription factors. EMBO J. *31*, 522–533.

Nichols, J., and Smith, A. (2009). Naive and primed pluripotent states. Cell Stem Cell *4*, 487–492.

Osteil, P., Moulin, A., Santamaria, C., Joly, T., Jouneau, L., Aubry, M., Tapponnier, Y., Archilla, C., Schmaltz-Panneau, B., Lecardonnel, J., et al. (2016). A panel of embryonic stem cell lines reveals the variety and dynamic of pluripotent states in rabbits. Stem Cel. Reports *7*, 383–398.

Osteil, P., Tapponnier, Y., Markossian, S., Godet, M., Schmaltz-Panneau, B., Jouneau, L., Cabau, C., Joly, T., Blachère, T., Gócza, E., et al. (2013). Induced pluripotent stem cells derived from rabbits exhibit some characteristics of naïve pluripotency. Biol. Open *2*, 613–628.

Sakaue-Sawano, A., Yo, M., Komatsu, N., Hiratsuka, T., Kogure, T., Hoshida, T., Goshima, N., Matsuda, M., Miyoshi, H., and Miyawaki, A. (2017). Genetically encoded tools for optical dissection of the mammalian cell cycle. Mol. Cell *68*, 626–640 e625.

Savatier, P., Lapillonne, H., van Grunsven, L.A., Rudkin, B.B., and Samarut, J. (1996). Withdrawal of differentiation inhibitory activity/leukemia inhibitory factor up-regulates D-type cyclins and cyclin-dependent kinase inhibitors in mouse embryonic stem cells. Oncogene *12*, 309–322.

Stead, E., White, J., Faast, R., Conn, S., Goldstone, S., Rathjen, J., Dhingra, U., Rathjen, P., Walker, D., and Dalton, S. (2002). Pluripotent cell division cycles are driven by ectopic Cdk2, cyclin A/E and E2F activities. Oncogene *21*, 8320–8333.

Tachibana, M., Sparman, M., Ramsey, C., Ma, H., Lee, H.S., Penedo, M.C., and Mitalipov, S. (2012). Generation of chimeric rhesus monkeys. Cell *148*, 285–295.

Takashima, Y., Guo, G., Loos, R., Nichols, J., Ficz, G., Krueger, F., Oxley, D., Santos, F., Clarke, J., Mansfield, W., et al. (2014). Resetting transcription factor control circuitry toward ground-state pluripotency in human. Cell *158*, 1254–1269.

Tapponnier, Y., Afanassieff, M., Aksoy, I., Aubry, M., Moulin, A., Medjani, L., Bouchereau, W., Mayère, C., Osteil, P., Nurse-Francis, J., et al. (2017). Reprogramming of rabbit induced pluripotent stem cells toward epiblast and chimeric competency using Krüppel-like factors. Stem Cell Res *24*, 106–117.

Ter Huurne, M., Chappell, J., Dalton, S., and Stunnenberg, H.G. (2017). Distinct cell-cycle control in two different states of mouse pluripotency. Cell Stem Cell *21*, 449–e4.

Theunissen, T.W., Friedli, M., He, Y., Planet, E., O'Neil, R.C., Markoulaki, S., Pontis, J., Wang, H., Iouranova, A., Imbeault, M., et al. (2016). Molecular criteria for defining the naive human pluripotent state. Cell Stem Cell *19*, 502–515.

Theunissen, T.W., Powell, B.E., Wang, H., Mitalipova, M., Faddah, D.A., Reddy, J., Fan, Z.P., Maetzel, D., Ganz, K., Shi, L., et al. (2014). Systematic identification of culture conditions for induction and maintenance of naive human pluripotency. Cell Stem Cell *15*, 524–526.

Ware, C.B., Wang, L., Mecham, B.H., Shen, L., Nelson, A.M., Bar, M., Lamba, D.A., Dauphin, D.S., Buckingham, B., Askari, B., et al. (2009). Histone deacetylase inhibition elicits an evolutionarily conserved self-renewal program in embryonic stem cells. Cell Stem Cell *4*, 359–369.

Wianny, F., Bernat, A., Huissoud, C., Marcy, G., Markossian, S., Cortay, V., Giroud, P., Leviel, V., Kennedy, H., Savatier, P., and Dehay, C. (2008). Derivation and cloning of a novel rhesus embryonic stem cell line stably expressing tau-green fluorescent protein. Stem Cells *26*, 1444–1453.

Wu, J., Platero-Luengo, A., Sakurai, M., Sugawara, A., Gil, M.A., Yamauchi, T., Suzuki, K., Bogliotti, Y.S., Cuello, C., Morales Valencia, M., et al. (2017). Interspecies chimerism with mammalian pluripotent stem cells. Cell *168*, 473–e15.

Yang, J., Ryan, D.J., Wang, W., Tsang, J.C., Lan, G., Masaki, H., Gao, X., Antunes, L., Yu, Y., Zhu, Z., et al. (2017a). Establishment of mouse expanded potential stem cells. Nature *550*, 393–397.

Yang, Y., Liu, B., Xu, J., Wang, J., Wu, J., Shi, C., Xu, Y., Dong, J., Wang, C., Lai, W., et al. (2017b). Derivation of pluripotent stem cells with in vivo embryonic and extraembryonic potency. Cell *169*, 243–e25.

Stem Cell Reports, Volume *16*

## Supplemental Information

# Apoptosis, G1 Phase Stall, and Premature Differentiation Account for Low Chimeric Competence of Human and Rhesus Monkey Naive Pluripotent Stem Cells

**Irène Aksoy, Cloé Rognard, Anaïs Moulin, Guillaume Marcy, Etienne Masfaraud, Florence Wianny, Véronique Cortay, Angèle Bellemin-Ménard, Nathalie Doerflinger, Manon Dirheimer, Chloé Mayère, Pierre-Yves Bourillot, Cian Lynch, Olivier Raineteau, Thierry Joly, Colette Dehay, Manuel Serrano, Marielle Afanassieff, and Pierre Savatier**

Stem Cell Reports, Volume *16*

## Supplemental Information

# Apoptosis, G1 Phase Stall, and Premature Differentiation Account for Low Chimeric Competence of Human and Rhesus Monkey Naive Pluripotent Stem Cells

**Irène Aksoy, Cloé Rognard, Anaïs Moulin, Guillaume Marcy, Etienne Masfaraud, Florence Wianny, Véronique Cortay, Angèle Bellemin-Ménard, Nathalie Doerflinger, Manon Dirheimer, Chloé Mayère, Pierre-Yves Bourillot, Cian Lynch, Olivier Raineteau, Thierry Joly, Colette Dehay, Manuel Serrano, Marielle Afanassieff, and Pierre Savatier**

**Inventory of Supplemental Information**

Figure S1

Figure S2

Figure S3

Figure S4

Figure S5

Figure S6

Figure S7

Table S1

Supplemental Experimental Procedures

Summary of media composition

List of antibodies

Supplemental References

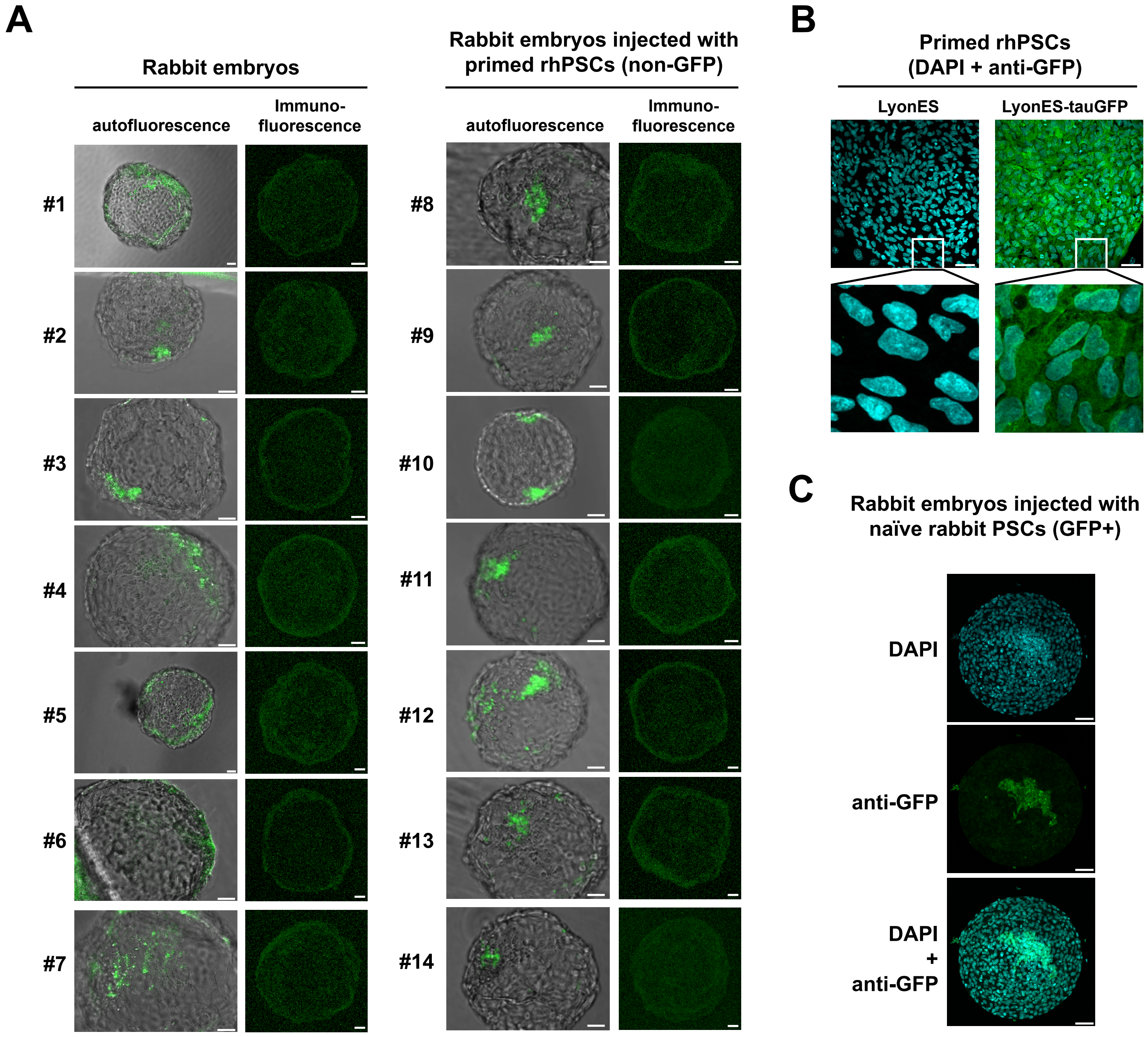


**Figure S1: Evaluation of autofluorescence in rabbit embryos. Related to Figure 1. (A)** Epifluoresence imaging and immunostaining of GFP in uninjected rabbit embryos (left panel) and in rabbit embryos injected with primed rhesus PSCs not expressing GFP (n = 40). **(B)** Immunostaining of GFP in primed LyonES-tGFP-(S3) rhesus PSCs. **(C)** Immunostaining of GFP in a late blastocyst-stage (E5) rabbit embryo after microinjection of rabbit naïve PSCs into morula-stage (E2) embryos (confocal imaging; scale bars: 50 $\mu$m).

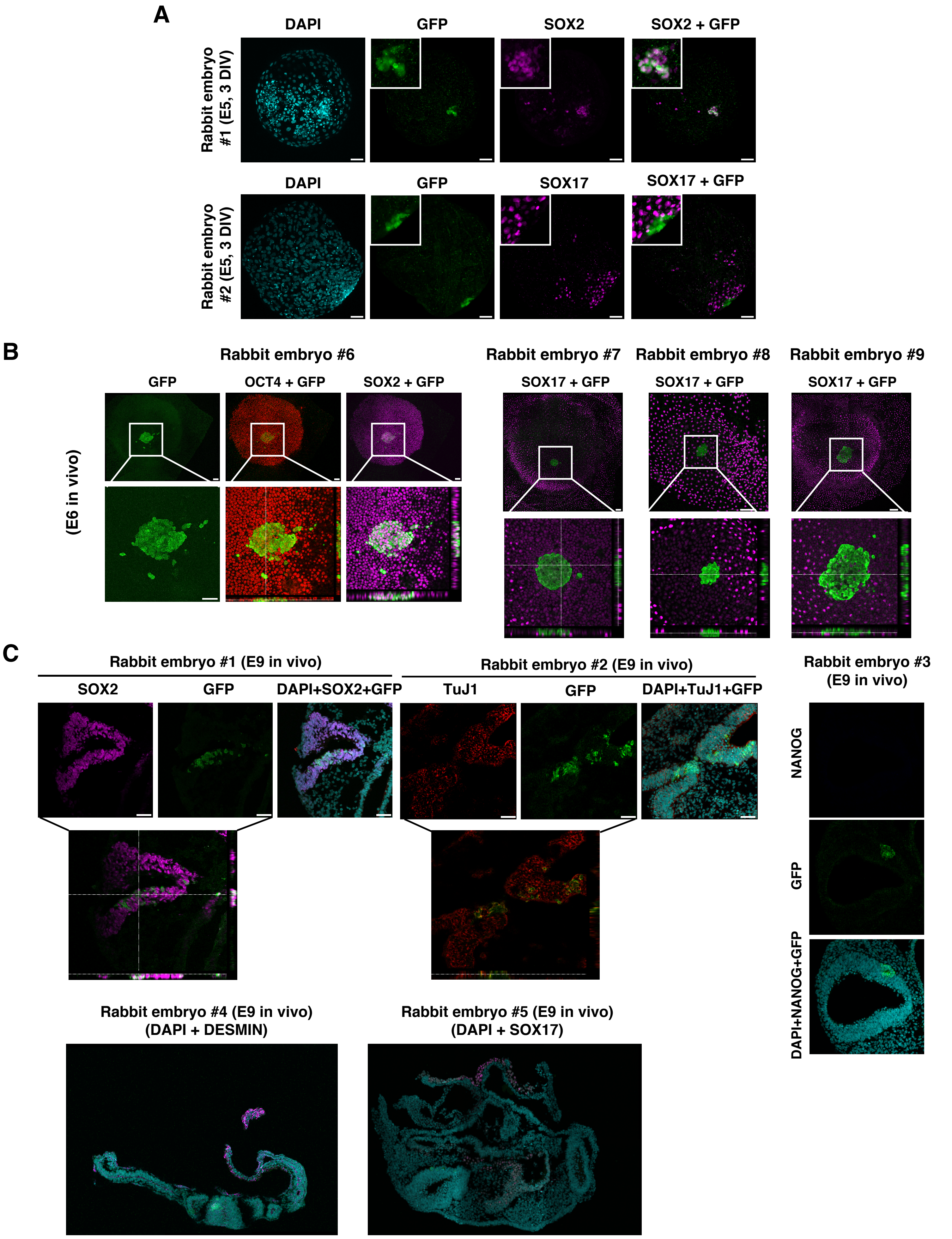


**Figure S2: Colonization of rabbit embryos by mouse ESCs. Related to Figure 1.** **(A)** Immunostaining of GFP, SOX2 and SOX17 in rabbit embryos at the late blastocyst-stage (E5) after microinjection of mESC-GFP-2i/LIF cells into morula-stage embryos (confocal imaging; scale bars: 50 $\mu$m) (n = 29). **(B)** Immunostaining of GFP, OCT4, SOX2 and SOX17 performed on rabbit embryos at the pre-gastrula stage (E6) after microinjection of mESC-GFP cells into morula-stage (E2) embryos and transfer to a surrogate mother. Bottom panels represent orthogonal sections. (confocal imaging; scale bars: 50 $\mu$m). **(C)** Immunostaining of GFP, SOX2, TuJ1, NANOG, DESMIN, and SOX17, in rabbit embryos at the post-implantation stage (E9) after microinjection of mESC-GFP cells into morula-stage embryos (confocal imaging; scale bars: 50 $\mu$m).

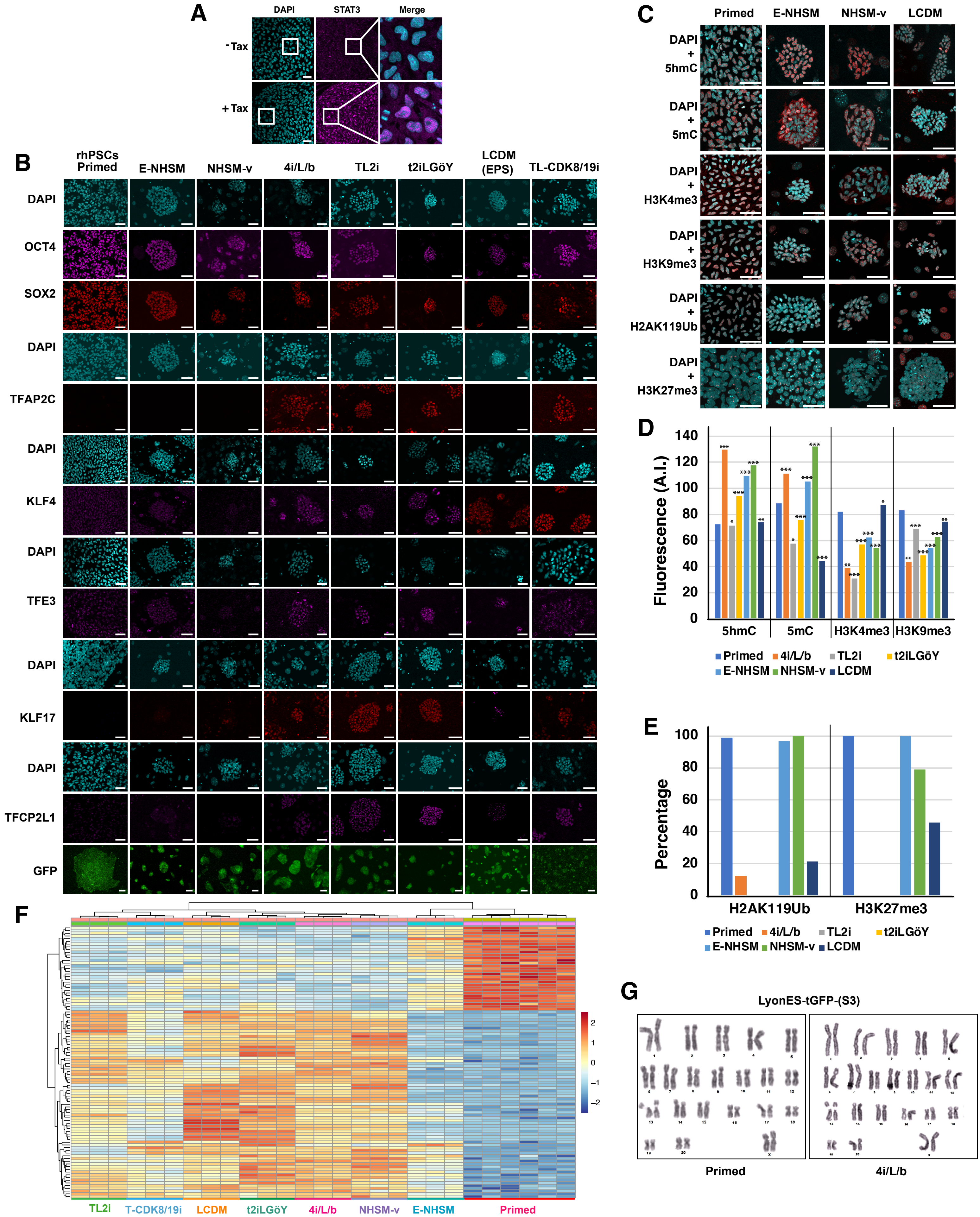


**Figure S3: Characterization of LyonES-tGFP-(S3) cells. Related to Figure 2.** **(A)** Immunolabeling of STAT3 in LyonES-tGFP-(S3) cells, before and after treatment with 250nM tamoxifen for 48 h (confocal imaging; scale bars: 50 $\mu$m). **(B) (C)** Immunolabeling of pluripotency markers and methylation marks in LyonES-tGFP-(S3) cells, before and after applying culture protocols for naïve conversion (confocal imaging; scale bars: 50 $\mu$m). **(D)** Histogram representing the quantification of 5hmC, 5mC, H3K4me3 and H3K9me3 methylation marks (Student's t test, p-value ≤ 0,05 : *; p-value ≤ 0,01 : **; p < 0,001: ***). **(E)** Histogram representing the percentage of H2AK119Ub and H3K27me3 marks. **(F)** Heatmap of transcriptome data (mean values/cell category most differentially expressed 100 genes, listed in Table S2) using Pearson correlation coefficient as a measure of Euclidian distance between rows and between columns. (G) G-banding karyotype of LyonES-tGFP-(S3) primed cells (left panel) and a batch of LyonES-tGFP-(S3) naïve 4i/L/b cells.

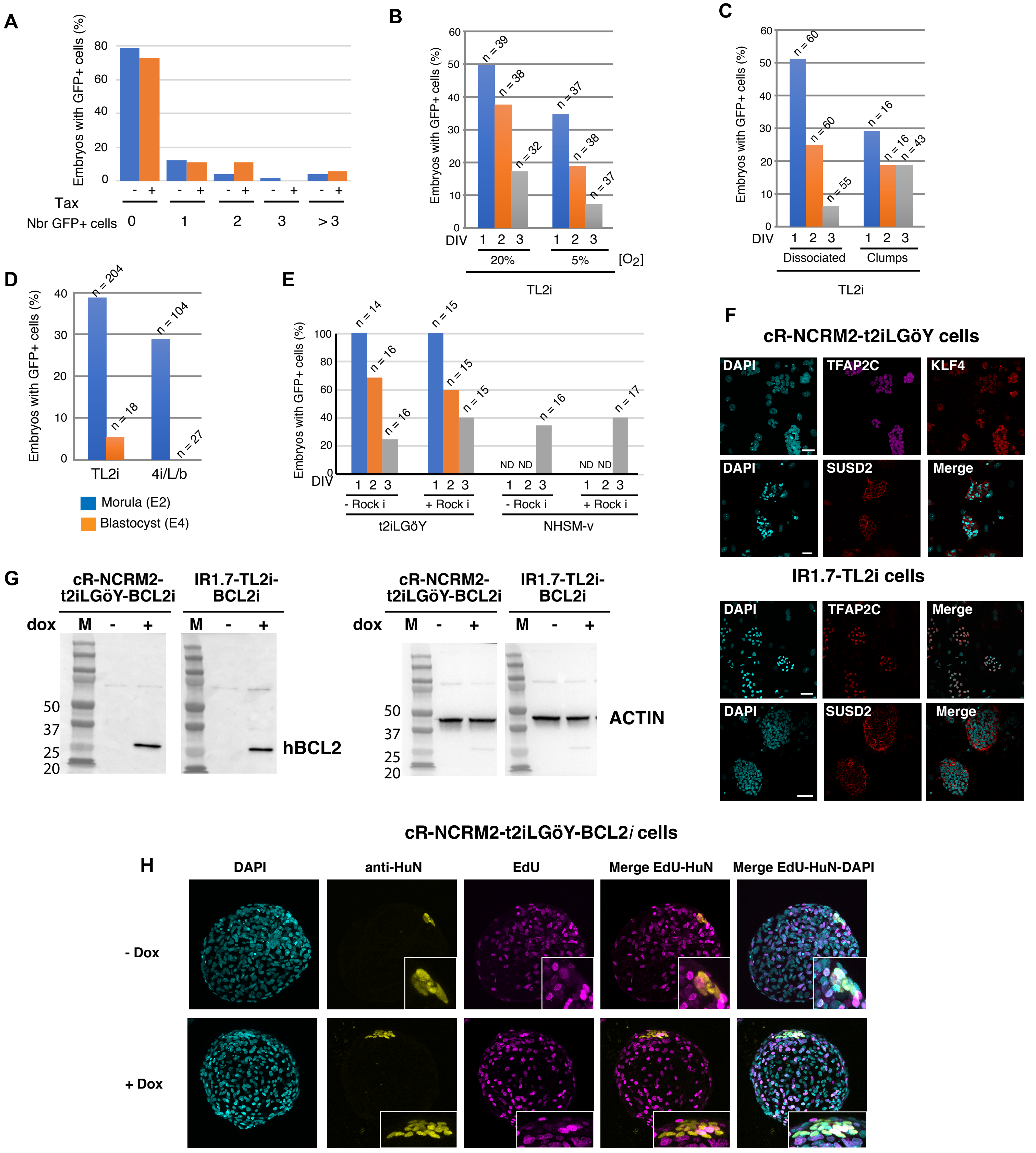


**Figure S4: Optimization of the conditions for microinjection and Colonization of rabbit embryos by human PSCs. Related to Figures 3 and 4. (A)** Histogram of percentage of rabbit embryos with GFP+ cells after microinjection of rhesus TL2i cells at the morula-stage (E2) and in vitro culture for 3 days in gradual medium in the presence (+) or in the absence (-) of 250nM tamoxifen. **(B)** Histogram of percentage of rabbit embryos with GFP+ cells after microinjection of rhesus TL2i cells at the morula-stage (E2) under normoxic (20%) and hypoxic (5%) conditions. Analyses were performed at 1, 2, and 3 DIV (n = 221). **(C)** Histogram of the percentage of rabbit embryos with GFP+ cells after microinjection of dissociated or clumps of rhesus TL2i cells into morula-stage (E2) embryos. Analyses were performed at 1, 2, and 3 DIV (n = 250). **(D)** Histogram of the percentage of rabbit embryos with GFP+ cells after microinjection of rhesus 4i/L/b and TL2i cells into morula-stage (E2) or blastocyst-stage (E4) embryos. Analyses were performed at 3 DIV (n = 353). **(E)** Histogram of the percentage of rabbit embryos with GFP+ cells after microinjection of rhesus t2iLGöY and E-NHSM cells into morula-stage (E2) embryos, in the presence or absence of ROCK inhibitor (n = 124). Analyses were performed at 3 DIV. **(F)** Immunostaining of TFAP2C and SUSD2 in NCRM2-t2iLGöY and IR1.7 TL2i cells (confocal imaging; scale bars: 50 $\mu$m). **(G)** Western blots of hBCL2 in cR-NCRM2- t2iLGöY and IR1.7-TL2i cells before (-) and after (+) treatment with 1 $\mu$g/mL doxycycline for 72 hours. **(H)** Immunostaining of HuN anf EdU in late blastocyst-stage rabbit embryos (E5, 3 DIV) after microinjection of human cR-NCRM2-t2iLGöY-BCL2*i* PSCs before (-Dox) and after (+Dox) treatment with 1 $\mu$g/mL doxycycline from 24 hours before injection to 72 hours after injection.

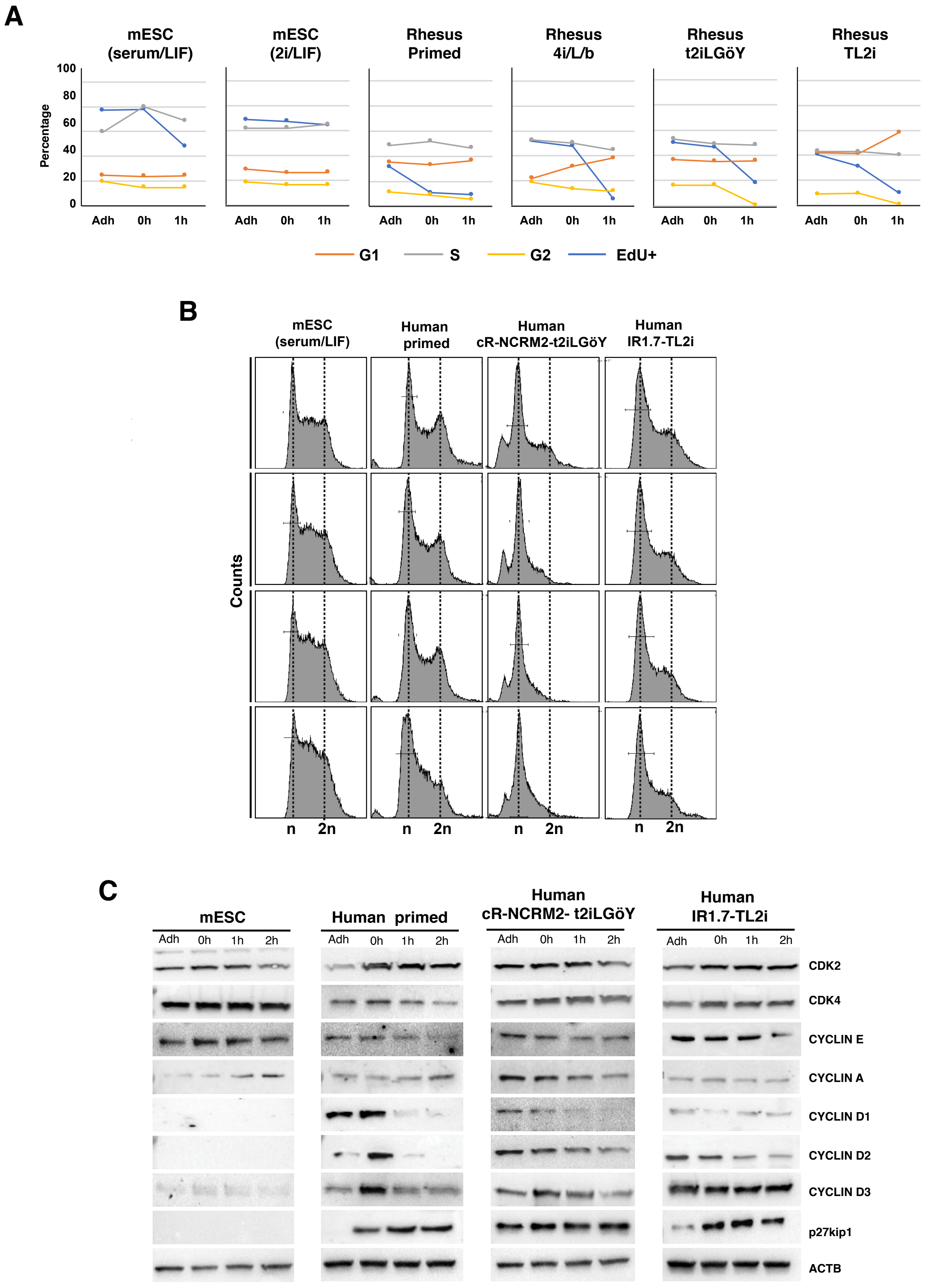


**Figure S5: Cell-cycle parameters of mouse ESCs, rhesus PSCs, and human iPSCs, before and after reprogramming to naïve states. Related to Figure 6. (A)** Histograms showing the cell cycle distribution of mESCs (serum/LIF and 2i/LIF) and rhesus PSCs (primed, 4i/L/b, t2iLGöY and TL2i) in Adh, 0h, 1h, and 2h conditions analyzed by flow cytometry after EdU incorporation and PI staining (% EdU+, G1, and S/G2 cells). **(B)** Cell cycle distribution of mESCs (serum/LIF) and human PSCs (primed, cR-NCRM2-t2iLGöY and IR7.1-TL2i) in Adh, 0h, 1h, and 2h conditions analysed by flow cytometry after PI staining. **(C)** Western blot analysis of cell-cycle regulators in mESCs (serum/LIF) and human iPSCs (primed, cR-NCRM2-t2iLGöY and IR7.1-TL2i) in Adh, 0h, 1h, and 2h conditions (results of 3 experiments).

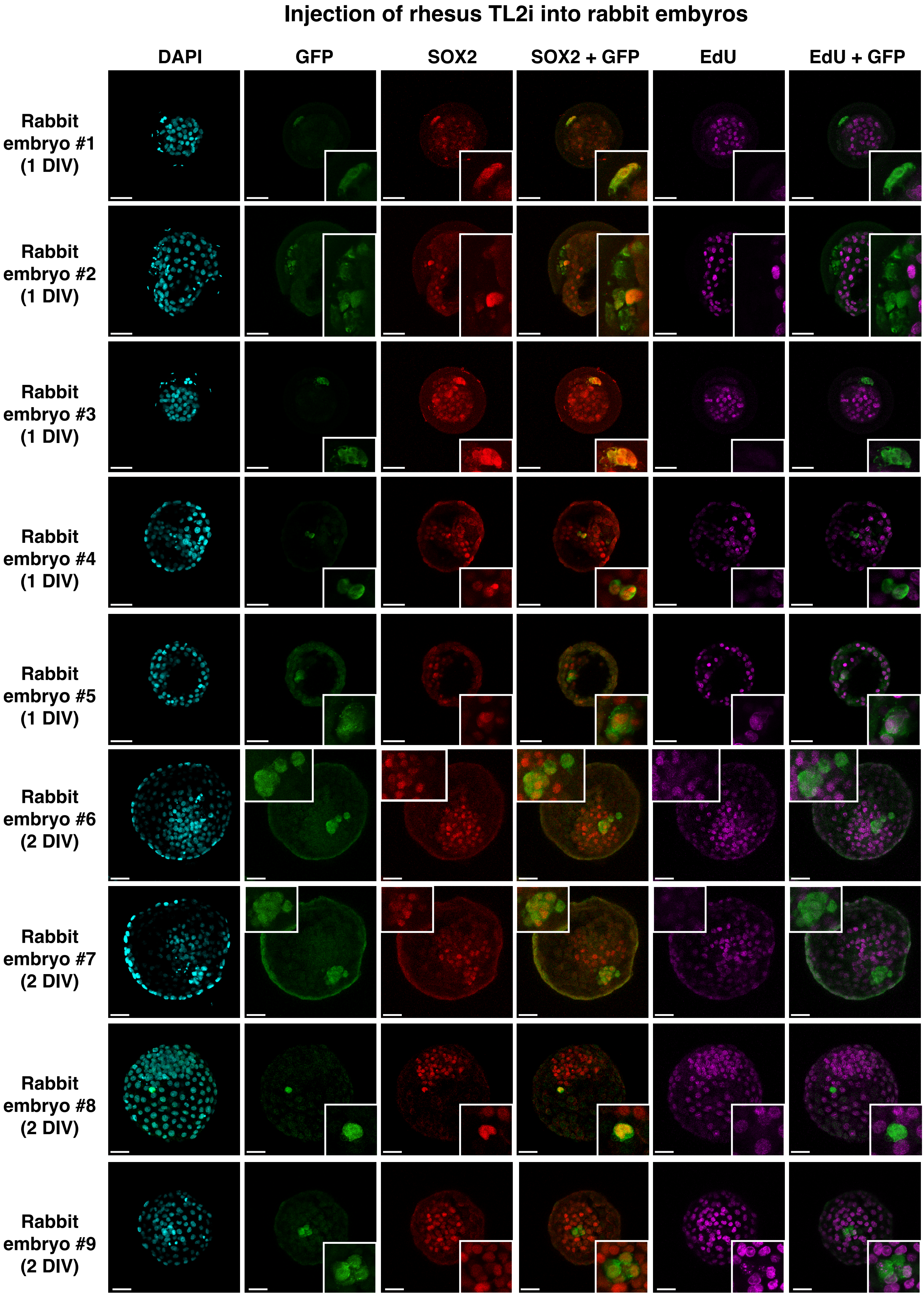


**Figure S6: DNA replication of Rhesus PSCs after microinjection into rabbit embryos. Related to Figure 7.** Immunostaining of GFP, Sox2, and EdU in late blastocyst-stage rabbit embryos (E5, 3 DIV) after microinjection of rhesus TL2i cells into morula-stage (E2) embryos (confocal imaging; scale bars: 50 $\mu$m).

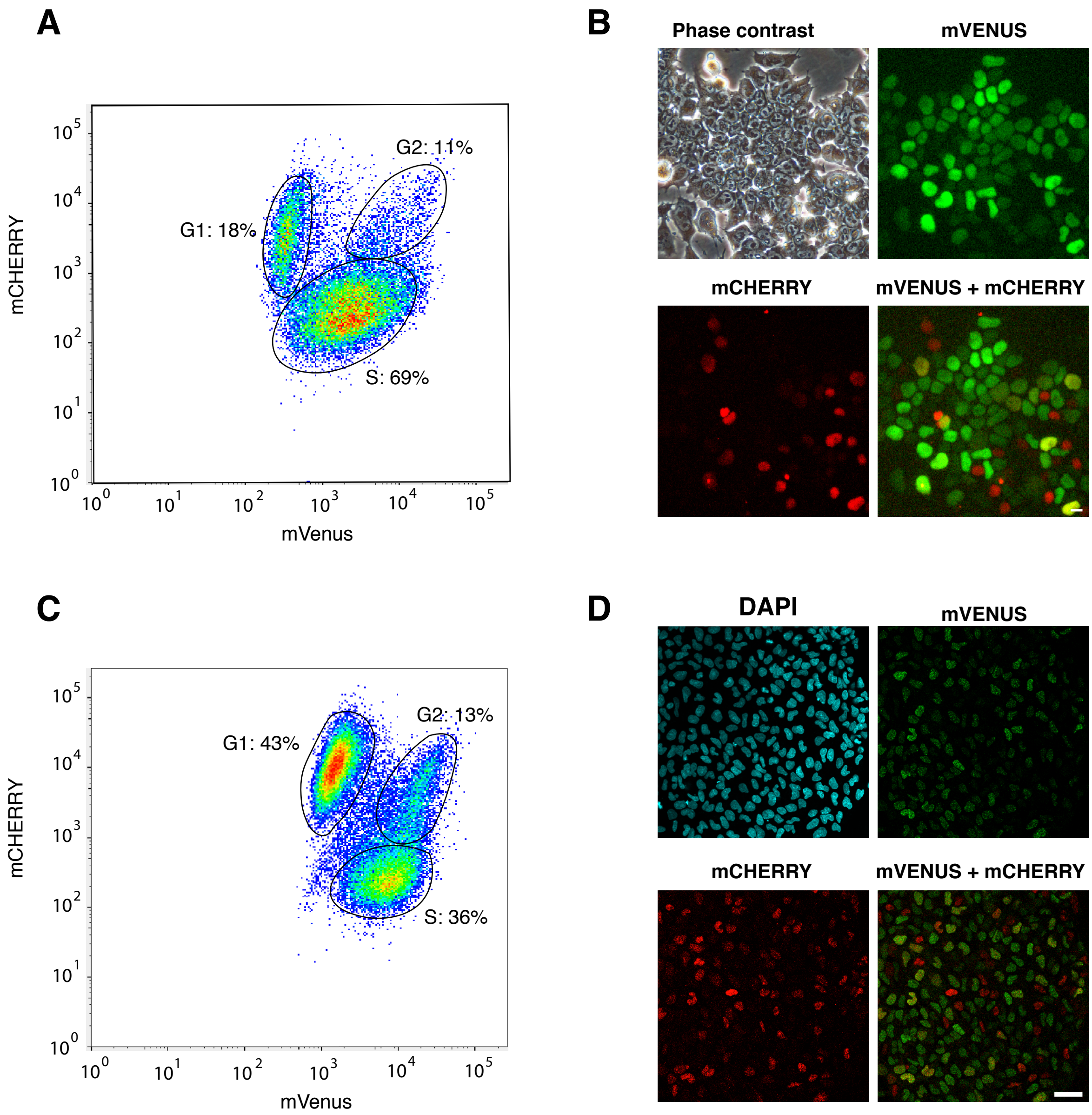


**Figure S7: Characterization of mouse ESCs and rhesus PSCs harboring the FUCCI(CA) cell cycle reporter. Related to Figure 7. (A)** Flow cytometry analysis and **(B)** epifluorescence imaging of mESCs-FUCCI (CA) cells. **(C)** Flow cytometry analysis and **(D)** epifluorescence imaging of rhesus primed-FUCCI (CA) cells (scale bars: 50 $\mu$m).

## Supplemental Experimental Procedures

### Cell lines, media composition, and culture

mESC lines were routinely cultured in Glasgow's modified Eagle's medium (Gibco) supplemented with 10% fetal calf serum (CRC0406; PerbioScience) and 1,000 U/mL LIF on gelatin-coated dishes. They were also cultured in N2B27 supplemented with 1,000 U/mL LIF, 1 μM PD0325901, and 3 μM CHIR99021 (2i/LIF), as indicated. Both the LyonES-tGFP and Lyon-ES-tGFP-(S3) rhesus PSC lines (Wianny et al., 2008, Chen et al., 2015a) and the human iPSC line IR7.1 (Chen et al., 2015a), (Ng et al., 2012) cell lines were routinely cultured at 37 °C in 5% $CO_2$ and 5% $O_2$ in knockout Dulbecco's modified Eagle's medium (KO-DMEM) supplemented with 20% knockout serum replacement (KOSR), 1 mM glutamine, 0.1 mM β-mercaptoethanol (Sigma), 1% non-essential amino acid (Gibco), and 4–8 ng/mL FGF2 (Gibco) on growth-inactivated murine embryonic fibroblasts.

Primed to naïve conversion was performed using previously described protocols, including E-NHSM (https://hannalabweb.weizmann.ac.il) (Gafni et al., 2013), NHSM-v (Chen et al., 2015b), 4i/L/b (Fang et al., 2014), 5iL/A (Theunissen et al., 2014), t2iLGöY (Guo et al., 2017), TL2i (Chen et al., 2015a), TL-CDK8/19i [modified from (Lynch et al., 2020)], and LCDM (EPS) (Yang et al., 2017a). Primed cells were dissociated and plated on fresh feeder cells for 24 h before shifting to the naïve culture media. Fresh medium was added daily to the culture plates. The medium composition for E-NHSM reprogramming (https://hannalabweb.weizmann.ac.il) (Gafni et al., 2013) was as follows: Neurobasal: DMEM-F12 (1:1) supplemented with N2B27 (Gibco), L-ascorbic acid, 0.5% KOSR, 10 ng/mL human LIF (Peprotech), 5 μM IWR1 (Sigma), 1.5 μM CHIR99021 (Miltenyi Biotec), 1 μM PD0325901 (Miltenyi Biotec), 2 μM BIRB796 (Axon), 5 μM SP600125 (Tocris), 2 μM Gö6983 (Tocris), 20 ng/mL activin A (Peprotech), and 1 μM CGP77675 (Axon). Cells were passaged every 3–4 days by single-cell dissociation using Accutase (Gibco). ROCK inhibitor (Y-27632; Miltenyi Biotec) was added during passaging for 24 h. The NHSM-v protocol is a modified version of the NHSM protocol applied to cynomolgus macaque ESCs (Chen et al., 2015b). These cells were cultured in KO-DMEM supplemented with 20% KOSR, 8 ng/mL FGF2 (Gibco), 10 ng/mL hLIF (Peprotech), 3 μM CHIR99021 (Miltenyi Biotec), 1 μM PD0325901 (Miltenyi Biotec), 10μM SP600125 (Tocris), and 10 μM SB203580 (Tocris). Cells were passaged every 3–4 days by single-cell dissociation using 1 mg/mL Accutase (Gibco). ROCK inhibitor (Y-27632; Miltenyi Biotec) was added during passaging for 24 h. The 4i/L/b media was adapted from the protocol published by Fang *et al.* (Fang et al., 2014). Cells were cultured in KO-DMEM supplemented with 20% KOSR (Gibco), 0.5 μM PD0325901 (Miltenyi Biotec), 3 μM CHIR99021 (Miltenyi Biotec), 10 μM SP600125 (Tocris), 10 μM SB203580 (Tocris), 10 ng/mL hLIF (Peprotech), and 2.5 ng/mL FGF2 (Gibco). Cells were passaged every 3–4 days by single-cell dissociation using 0.05% Trypsin-ethylenediaminetetraacetic acid (Trypsin-EDTA; Gibco). ROCK inhibitor (Y-27632; Miltenyi Biotec) was added during passaging for 24 h. Primed cells were converted to 5i/L/A state using the following medium composition (Theunissen et al., 2014): DMEM-F12 (1:1) medium supplemented with N2 (Gibco), B27 (Gibco), 0.5% KOSR (Gibco), 1 μM PD0325901 (Miltenyi Biotec), 1 μM IM-12 (Enzo), 0.5 μM SB590885 (Tocris), 1 μM WH-4-023 (Tocris), and 20 ng/mL activin A (Peprotech). ROCK inhibitor (Y-27632; Miltenyi Biotec) was added during passaging for 24 h. Primed cells were converted to the t2iLGöY state using the following medium composition (Guo et al., 2017): first in cRM1 media for three days with 1 μM PD0325901 (Miltenyi Biotec), 10 ng/mL hLIF (Peprotech) and 1mM Valproic acid (VPA), followed by two days in cRM2 media with DMEM-F12 (1:1) medium supplemented with N2, B27 (Gibco), 1 μM PD0325901 (Miltenyi Biotec) and 250 μM Vitamin C (Sigma-Aldrich) and then the final media containing DMEM-F12 (1:1) medium supplemented with N2, B27 (Gibco), 1 μM PD0325901 (Miltenyi Biotec), 1,000 U/mL LIF, 1 μM Gö6983 (Bio-techne), 2 μM XAV939 (Sigma). Cells were passaged every 4–5 days by single-cell dissociation using Tryple (Gibco). ROCK inhibitor (Y-27632; Miltenyi Biotec) was added during passaging for 24 h. For reprogramming to the TL2i state, PSCs expressing the inducible fusion protein STAT3-$ER^{T2}$ were cultivated in the TL2i medium composed of KO-DMEM (Gibco), 20% KOSR (Gibco), 10,000 U/mL LIF, 3 μM CHIR99021 (Miltenyi Biotec), 1 μM PD0325901 (Miltenyi Biotec), and 250 nM 4′-Hydroxy-Tamoxifen (4′OHT; Calbiochem). Cells were passaged every 3–4 days by single-cell dissociation using 0.05% Trypsin-EDTA (Gibco). ROCK inhibitor (Y-27632; Miltenyi Biotec) was added during passaging for 24 h. For TL-CDK8/19i protocol, cells were cultured in medium composed of KO-DMEM (Gibco), 20% KOSR (Gibco), 10,000 U/mL LIF, and 1 μM CNIO-47799. Cells were passaged every 3–4 days by single-cell dissociation using 0.05% Trypsin-EDTA (Gibco). ROCK inhibitor (Y-27632; Miltenyi Biotec) was added during passaging for 24 h.Primed cells were converted to the LCDM (EPS) (Yang et al., 2017) state using medium with the following composition: DMEM-F12 (1:1), N2 (Gibco), B27 (Gibco), 5% KOSR (Gibco), 10 ng/mL hLIF (Peprotech), 1 μM CHIR99021 (Miltenyi Biotec), 2 μM (S)-(+)-Dimethindene maleate (Tocris), 2 μM minocycline hydrochloride (Santa Cruz Biotechnology), and 0.75 μM IWR1 (Sellekchem). Cells were passaged every 3–4 days by single-cell dissociation using 0.05% Trypsin-EDTA (Gibco). ROCK inhibitor (Y-27632; Miltenyi Biotec) was added during passaging for 24 h. All media compositions are summarized below.

To avoid the accumulation of karyotypic abnormalities, new naïve cell batches were regularly produced and none of the naïve cells were cultured for more than 2 months after reprogramming.

**G-banding karyotyping**

G-banding karyotyping was performed by Cell Guidance Systems (www.cellgs.com).

**Generation of FUCCI reporter rhesus PSCs and mESCs**

The *PB-Puro$^R$-CAG-mVenus : hGeminin-IRES-mCherry : hCdt1* plasmid was generated as follows. First, an EcoRI/XbaI DNA fragment from the *mCherry-hCdt1(1/100)Cy(-)/pcDNA3* plasmid (Sakaue-Sawano et al., 2017) containing the mCherry : hCdt1 cassette was sub-cloned between the EcoRI and XbaI restriction sites in *PB-CAG-cHA-IRES-hyg* to generate *PB-Hygro-mcherry : hCdt1*. Second, a AlfII/XbaI fragment from the *mVenus-hGeminin(1/110)/pcDNA3* plasmid (Sakaue-Sawano et al., 2017) containing the mVenus : hGeminin cassette was subcloned between the SapI and AgeI restriction sites in *PB-Hygro-mcherry : hCdt1* to generate *PB-Hygro-mcherry : hCdt1-mVenus : hGeminin*. Third, a SapI/AgeI fragment containing the *Puro$^R$* gene and the CAG promoter was sub-cloned between the SapI and AgeI restriction sites in *PB-Hygro-mcherry : hCdt1-mVenus : hGeminin* to generate the *PB-Puro$^R$-CAG-mVenus : hGeminin-IRES-mCherry : hCdt1* plasmid. Rhesus PSCs and mESCs were transfected using the NEON transfection system according to the instructions provided by the manufacturer. Briefly, cells were dissociated and resuspended at a density of $10.10^6$ cells/mL. For transfection, 100 µL of the cell suspension was mixed with 2.5 µg of the *PB-Puro$^R$-CAG-mVenus : hGeminin-IRES-mCherry : hCdt1* vector and 2.5 µg of *PBase* plasmid. Transfection parameters used for rhesus PSCs were 1,050 V, 20 ms, and 2 pulses. Cells were plated on growth-inactivated murine embryonic fibroblasts in medium supplemented with 10 µM ROCK inhibitor (Y-27632; Miltenyi Biotec) and selected in 250 ng/mL G418. For mESCs, the parameters used were 1,200 V, 20 ms, and 2 pulses. Cells were analyzed using a FACS LSR II (Becton-Dickinson) equipped with 355, 488, and 561 nm lasers. Data were acquired and analyzed using the DiVa software.

**Generation of BCL2 inducible human PSCs**

The *BCL2* plasmid was generated as follows. First, a DNA fragment from the *pCS2+Flag-BCL2* plasmid was amplified by PCR using primers flanked by *Kpn*I and *Spe*I restriction sites at the 5' and 3' end, respectively. The PCR fragment was then subcloned in pXlone-GFP-Neo3R-P2A-TRE-EF1 plasmid. This plasmid was obtained after modification of the *Xlone-GFP* plasmid (a gift from Xiaojun Lian, Addgene plasmid #96930) in which the Blasticidin resistance gene was replaced by Neomycin. cR-NCRM2-t2iLGöY and IR1.7-TL2i cells were transfected using the NEON transfection system according to the instructions provided by the manufacturer. Briefly, cells were dissociated and resuspended at a density of $10.10^6$ cells/mL. For transfection, 100 µL of the cell suspension was mixed with 2.5 µg of the *pXlone-Flag-BCL2-Neo3R-P2A-TRE-EF1* vector and 2.5 µg of *PBase* plasmid. Transfection parameters used were 1,050 V, 20 ms, and 2 pulses. Cells were plated on growth-inactivated murine embryonic fibroblasts in medium supplemented with 10 µM ROCK inhibitor (Y-27632; Miltenyi Biotec) and selected in 250 ng/mL G418.

**Generation of cynomolgus monkey embryos and cell microinjection**

Macaque cynomolgus embryos were produced through ovarian stimulation, followed by intra-cytoplasmic sperm injection (ICSI) of oocytes, as previously described (Tachibana et al., 2012). Briefly, females received twice-daily injections of 25 IU of recombinant human follicle-stimulating hormone (MSD laboratories) from days 1 to 8 (starting at day 1–4 of the menstrual cycle). They received 30 IU of recombinant human luteinizing hormone (Merck) on days 7 and 8. Females received an injection of gonadotropin-releasing hormone antagonist (MSD laboratories) (0.075 mg/kg body weight) on day 8, and an injection of 1,040 IU of human chorionic gonadotropin (Merck) at day 8. Estradiol, luteinizing hormone, progesterone dosage, and ultrasonographic scans were performed to monitor ovarian response. Follicle aspiration and oocyte retrieval were performed by laparoscopy 36 h after injection of human chorionic gonadotropin. Oocytes were transferred to HEPES-buffered TALP medium containing 3 mg/mL of bovine serum albumin (Sigma). Cumulus and granulosa cells were removed by incubating the oocytes for 30 s with hyaluronidase (300 µg/mL). Metaphase II stage oocytes were selected for ICSI and transferred to drops of hamster embryo culture medium-9 (HECM9) covered by liquid paraffin (Origio) at 37 °C in a 5% $O_2$ + 5% $CO_2$ atmosphere. Four days after fertilization (E4), morula-stage embryos were microinjected with 10 cells and further cultured in drops of PSC medium for 4 h. PSC media were those used for culturing the cells prior to injection, depending on the reprogramming protocols. After 4 h, embryos were transferred into a 1:1 mix of PSC medium and HECM9 and further cultured for 20 h. After 24 h, embryos were transferred to HECM9 and further cultured.

**Production of rabbit embryos, cell microinjection, embryo culture, and transfer**

Rabbit embryos were produced by ovarian stimulation. Briefly, sexually mature New Zealand white rabbits were injected with follicle-stimulating hormone and gonadotropin-releasing hormone, followed by artificial insemination or breeding, as previously described. Eight-cell-stage embryos (E1.5) were flushed from

explanted oviducts 36–40 h after insemination and cultured in a 1:1:1 mixture of RPMI 1640 medium, DMEM, and Ham's F10 (RDH medium; Thermo Fisher Scientific) at 38 °C in 5% $CO_2$ until cell microinjection. For the latter procedure, 10 cells were microinjected under the zona pellucida of morula (E2.5)- or blastocyst (E4)-stage rabbit embryos. The embryos were further cultured using the same experimental procedure as for monkey embryo. For embryo transfer, surrogate mothers were prepared through intramuscular injection of 1.6 µg of buserelin acetate (Intervet). Morula-stage embryos (6–8) were transferred to each oviduct of the recipient by laparoscopy. Four days after transfer, pre-implantation embryos (E6) were recovered by flushing the explanted uterine horns. Post-implantation-stage embryos (E9) were recovered by dissecting the uterine horns.

**Immunoblotting**

For immunoblotting, cells were grown on Geltrex to avoid contamination by mouse embryonic fibroblasts. Frozen cell pellets were lysed in RIPA buffer complemented with protease and phosphatase inhibitors. Protein lysates were then cleared by centrifugation (14,000 r.p.m. for 30 min). After SDS–PAGE and electroblotting on polyvinylidene difluoride, the membranes were incubated with specific primary antibodies (antibodies used in this study are listed below). Blots were incubated with horseradish peroxidase-coupled anti-mouse or -rabbit immunoglobulin G (Jackson ImmunoResearch) and developed with Clarity Western ECL Substrate (BIO-RAD).

**Histology, histochemistry, immunostaining**

Monkey (E7) and rabbit (E3, E4, E5, and E6) pre-implantation embryos were fixed in 2% paraformaldehyde (PFA) for 20 min at room temperature. After three washes in phosphate-buffered saline (PBS) containing 0.1% Tween-20 (PBS-0.1%T), embryos were permeabilized in PBS-1%T overnight at 4 °C on a rotating shaker. Embryos were subsequently placed in blocking solution (PBS-0.1%T) supplemented with 5% donkey serum for 1 h at room temperature. There were incubated with primary antibodies diluted in blocking solution overnight at 4°C (antibodies used in this study are listed below). After four washes (3 × 5 min + 1 × 30 min) in PBS-0.1%T, embryos were incubated in secondary antibodies diluted in blocking solution at a concentration of 1:300 for 1 h at room temperature and transferred through several washes of PBS-0.1%T before staining the nuclei with 4′,6-diamidino-2-phenylindole (DAPI; 0.5 µg/mL). Embryos were analyzed by confocal imaging (DM 6000 CS SP5; Leica). Acquisitions were performed using a water immersion objective (25×/1.25 0.75, PL APO HCX; Leica).

E8 rabbit post-implantation embryos were fixed in 2% PFA at 4 °C for 1 h. For cryoprotection, embryos were placed in 10% sucrose for 1 h followed by 30% sucrose overnight at 4 °C. Embryos were embedded in NEG50 compound (Invitrogen) and frozen at −80 °C. Sections of 15–20 µm were generated using a Microm HM550 cryostat and maintained at −80 °C until immunostaining was performed. For immunostaining, sections were thawed for 30 min at room temperature, saturated with PBS for 15 min at room temperature, and permeabilized (as necessary) with three baths of PBS containing 0.5% Triton X100 (Sigma-Aldrich). Sections were incubated in blocking solution: PBS with 0.1% Triton X100 and 10% donkey serum (Jackson Immunoresearch Laboratory) for 30 min. Subsequently, the sections were incubated with primary antibodies overnight at 4 °C. After several washes, the sections were incubated with the appropriate fluorescence-conjugated secondary antibodies at room temperature for 1 h. Nuclei were stained with DAPI (0.5 µg/mL). Sections were mounted on coverslips with Fluoromount G (Thermo Fisher Scientific). Tiled scans were automatically acquired using the LAS AF software (Leica).

Cells were fixed with 2% PFA in PBS at room temperature for 20 min, washed thrice (10 min each) with PBS and permeabilized in PBS containing 0.5% Triton X100. Non-specific binding sites were blocked using PBS supplemented with 10% donkey serum for 1 h at room temperature. The cells were incubated overnight at 4 °C with primary antibodies. After three rinses (10 min each) with PBS, the cells were incubated with secondary antibodies at room temperature for 1 h. The nuclei were stained with DAPI (0.5 µg/mL). The cells were mounted on coverslips by using the mounting medium M1289 (Sigma). Cells and embryo sections were analyzed by confocal imaging (DM 6000 CS SP5; Leica). Acquisitions were performed using an oil immersion objective (45×/1.25 0.75, PL APO HCX; Leica).

EdU staining was performed using the Click-iT™ Edu Imaging kit (Fisher Scientific). Briefly, embryos microinjected with PSCs were incubated for 1 h with 10 µM EdU in RDH medium. Embryos were fixed with 2–4% PFA, and immunostaining was performed according to the instructions provided by the manufacturer. Embryos were analyzed by confocal imaging (DM 6000 CS SP5; Leica), and acquisitions were performed using a water immersion objective (25×/1.25 0.75, PL APO HCX; Leica).

**Two-photon recording**

Embryos microinjected with rhesus and mESCs were placed in drops (5 µL) of medium and covered with liquid paraffin (Origio). For recording, embryos were cultured under the inverted Axio-Observer Z1 (Zeiss) two-photon microscope for 3 days, until they reach the E5 stage. Images were acquired using the Zeiss Zen software, and image analysis was performed using the ImageJ software.

**Flow cytometry analysis of cell cycle distribution**

Cells were labeled for 15 minutes with 10µM EdU in the culture medium using the Click-it Plus EdU flow cytometry kit (ThermoFischer). Cells were then washed and processed for the detection of incorporated EdU according to the manufacturer's instructions. After immunostaining, cells were incubated for 20 min with 1 mg/ml RNAse in PBS-0.13 mM EGTA. Propidium iodide (1 µg/ml) was added just before analysis. Cells were analyzed using a FACS LSR II (Becton-Dickinson) equipped with 355, 488, and 561 nM lasers. Data were acquired and analyzed using DiVa software.

**RNA sequencing**

RNA from cell lines was extracted from 4–5.$10^6$ cells using the RNeasy mini-kit (Qiagen). The libraries were prepared using 200 ng of RNA with the NextFlex Rapid Directional mRNA-Seq kit (Bioo-Scientific). Samples were sequenced on a NextSeq500 sequencing machine (Illumina) as single reads of 75 bp. The bcl2fastq conversion software was used for demultiplexing (Illumina), and data trimming was performed using Cutadapt. The sequencing depth for each sample was approximately 30 million reads, which were subsequently mapped to the Mmul8 rhesus macaque genome using the HISAT2 alignment program and quantified with the HTseq script.

**Bioinformatics analysis**

RNA-seq data obtained from rhesus primed and naïve cell lines were analyzed using the R software. PCA, differential expression analysis (with false discovery rate <0.1), and hierarchical clustering were performed with DESeq2 (Love et al., 2014). For cell lines, PCA was performed using the top 500 genes selected by highest row variance. For the comparison of cell lines with embryo data, we used the Seurat R package (version 3.1). Briefly, the two compendia of datasets were transformed to a Seurat object and merged. The transcript counts were subsequently log-transformed and normalized. Finally, the variable genes (3,000) were used as input for PCA.

**Table S1:** Summary of interspecies chimera experiments. Related to Figure 1, 2, 4 and 5.

| | Rabbit host | | |
|---|---|---|---|
| | E3 (1 DIV) | E4 (2 DIV) | E5 (3 DIV) |
| Rhesus PSCs | | | |
| Primed | | | |
| **Nb GFP+ embryos/injected embryos** | **10/104** | **0/89** | **0/90** |
| Nb NANOG+ embryos/GFP+ embryos | 0/7 | | |
| Nb SOX2+ embryos/GFP+ embryos | 0/2 | | |
| Nb GATA6+ embryos/GFP+ embryos | 0/1 | | |
| TL2i | | | |
| **Nb GFP+ embryos/injected embryos** | **109/141** | **78/145** | **79/204** |
| Nb NANOG+ embryos/GFP+ embryos | 14/16 | 7/12 | 2/7 |
| Nb SOX2+ embryos/GFP+ embryos | 45/53 | 30/52 | 8/40 |
| Nb GATA6+ embryos/GFP+ embryos | 6/31 | 8/22 | 14/33 |
| 4i/L/b | | | |
| **Nb GFP+ embryos/injected embryos** | **45/59** | **32/65** | **30/104** |
| Nb NANOG+ embryos/GFP+ embryos | 5/7 | 3/12 | 1/7 |
| Nb SOX2+ embryos/GFP+ embryos | 3/5 | 0/1 | 1/6 |
| Nb GATA6+ embryos/GFP+ embryos | 4/33 | 5/19 | 6/17 |
| t2iLGoY | | | |
| **Nb GFP+ embryos/injected embryos** | **92/96** | **72/97** | **69/114** |
| Nb NANOG+ embryos/GFP+ embryos | 10/40 | 0/21 | 0/11 |
| Nb SOX2+ embryos/GFP+ embryos | 14/43 | 6/31 | 3/10 |
| Nb GATA6+ embryos/GFP+ embryos | 3/39 | 10/38 | 8/20 |
| LCDM (EPS) | | | |
| **Nb GFP+ embryos/injected embryos** | **66/89** | **36/89** | **44/157** |
| Nb NANOG+ embryos/GFP+ embryos | 0/19 | 2/12 | 10/17 |
| Nb SOX2+ embryos/GFP+ embryos | 20/31 | 11/15 | 5/20 |
| Nb GATA6+ embryos/GFP+ embryos | 1/24 | 0/13 | 2/19 |
| E_NHSM | | | |
| **Nb GFP+ embryos/injected embryos** | **15/71** | **1/70** | **0/71** |
| Nb NANOG+ embryos/GFP+ embryos | 0/24 | | |
| Nb SOX2+ embryos/GFP+ embryos | 0/20 | | |
| Nb GATA6+ embryos/GFP+ embryos | 2/27 | | |
| NHSM_v | | | |
| **Nb GFP+ embryos/injected embryos** | **11/68** | **8/84** | **10/118** |
| Nb NANOG+ embryos/GFP+ embryos | 0/3 | | |
| Nb SOX2+ embryos/GFP+ embryos | 1/3 | 0/3 | 0/7 |
| Nb GATA6+ embryos/GFP+ embryos | 0/5 | 0/5 | 1/3 |
| TL_CDK8/19ii | | | |
| **Nb GFP+ embryos/injected embryos** | **43/79** | **19/80** | **10/101** |
| Nb NANOG+ embryos/GFP+ embryos | 10/20 | 2/7 | 1/4 |
| Nb SOX2+ embryos/GFP+ embryos | 2/12 | 2/11 | 1/5 |
| Nb GATA6+ embryos/GFP+ embryos | 6/15 | 1/3 | 0/1 |
| Mouse ESCs | | | |
| Serum + LIF | | | |
| **Nb GFP+ embryos/injected embryos** | | | **235/238** |
| Nb NANOG+ embryos/GFP+ embryos | | | 77/77 |
| Nb SOX2+ embryos/GFP+ embryos | | | 58/58 |
| Nb SOX17+ embryos/GFP+ embryos | | | 0/30 |
| 2iLIF | | | |
| Nb GFP+ embryos/injected embryos | | | 19/29 |
| Nb SOX2+ embryos/GFP+ embryos | | | 7/7 |
| Nb SOX17+ embryos/GFP+ embryos | | | 0/7 |
| **Total** | **775** | **786** | **1226** |
| **Total injected embryos** | **2787** | | |

| | Rabbit host | | |
|---|---|---|---|
| | E3 (1 DIV) | E4 (2 DIV) | E5 (3 DIV) |
| Human PSCs | | | |
| hiPSCs_t2iLGoY | | | |
| **Nb GFP+ embryos/injected embryos** | **27/29** | **20/31** | **97/350** |
| Nb NANOG+ embryos/GFP+ embryos | | | 5/23 |
| Nb SOX2+ embryos/GFP+ embryos | 24/29' | 11/20 | 45/97 |
| Nb GATA6+ embryos/GFP+ embryos | | | 3/14 |
| Nb SOX17+ embryos/GFP+ embryos | | | 15/30 |
| Nb T-BRA+ embryos/GFP+ embryos | | | 4/11 |
| Nb NESTIN+ embryos/GFP+ embryos | | | 0/5 |
| hiPSCs_TL2i | | | |
| **Nb GFP+ embryos/injected embryos** | **33/39** | **18/36** | **75/298** |
| Nb NANOG+ embryos/GFP+ embryos | 15/15 | 3/7 | 0/9 |
| Nb SOX2+ embryos/GFP+ embryos | 9/11 | 4/8 | 2/33 |
| Nb GATA6+ embryos/GFP+ embryos | 1/7 | 2/3 | 8/22 |
| Nb SOX17+ embryos/GFP+ embryos | | | 26/30 |
| Nb T-BRA+ embryos/GFP+ embryos | | | 0/7 |
| Nb NESTIN+ embryos/GFP+ embryos | | | 0/5 |
| **Total** | **68** | **67** | **648** |
| **Total injected embryos** | **783** | | |

**BCL2 inducible cell lines**

| | Rabbit host | |
|---|---|---|
| | E5 (3 DIV) | |
| hiPSCs_t2iLGoY_BCL2 | | |
| | **-Dox** | **+Dox** |
| **Nb GFP+ embryos/injected embryos** | **20/66** | **82/82** |
| Nb NANOG+ embryos/GFP+ embryos | 10/26 | 15/27 |
| Nb SOX2+ embryos/GFP+ embryos | 8/20 | 0/28 |
| Nb GATA6+ embryos/GFP+ embryos | 4/20 | 4/27 |
| hiPSCs_TL2i_BCL2 | | |
| | **-Dox** | **+Dox** |
| **Nb GFP+ embryos/injected embryos** | **26/77** | **62/76** |
| Nb NANOG+ embryos/GFP+ embryos | 0/26 | 0/26 |
| Nb SOX2+ embryos/GFP+ embryos | 13/26 | 3/25 |
| Nb GATA6+ embryos/GFP+ embryos | 25/25 | 19/25 |
| **Total** | **143** | **158** |
| **Total injected embryos** | **301** | |

| | Rabbit host | |
|---|---|---|
| | E6 | E9 |
| Mouse ESCs | | |
| **Nb GFP+ embryos/injected embryos** | **9/9** | **12/20** |
| Nb SOX2+ embryos/GFP+ embryos | 6/6 | |
| Nb SOX17+ embryos/GFP+ embryos | 3/3 | |
| Rhesus TL2i | | |
| **Nb GFP+ embryos/injected embryos** | | **0/16** |
| **Total** | **18** | **36** |
| **Total injected embryos** | **54** | |

| | Cynomolgus host |
|---|---|
| | E7 |
| Rhesus TL2i | |
| **Nb GFP+ embryos/injected embryos** | **2/7** |
| Nb NANOG+ embryos/GFP+ embryos | 0/2 |
| hiPSCs_TL2i | |
| **Nb GFP+ embryos/injected embryos** | **2/7** |
| Nb NANOG+ embryos/GFP+ embryos | 0/2 |
| hiPSCs_t2iLGoY | |
| **Nb GFP+ embryos/injected embryos** | **2/15** |
| Nb NANOG+ embryos/GFP+ embryos | 1/2 |
| Mouse ESCs | |
| **Nb GFP+ embryos/injected embryos** | **3/7** |
| Nb NANOG+ embryos/GFP+ embryos | 1/1 |
| Nb SOX2+ embryos/GFP+ embryos | 2/2 |
| **Total injected embryos** | **36** |

Total embryos injected in this study : 3961

## Summary of culture media used in this study

| | E-NHSM | NHSM-v | 4i/L/b | TL2i | t2iLGöY | LCDM (EPS) | TL-CDK8/19i | 5i/L/A |
|---|---|---|---|---|---|---|---|---|
| **Media** | | | | | | | | |
| Neurobasal:DMEMF12 (1:1) | ✓ | | | | ✓ | ✓ | | ✓ |
| KO-DMEM | | ✓ | ✓ | ✓ | | | ✓ | |
| N2B27 | ✓ | | | | ✓ | ✓ | | ✓ |
| KOSR | 0.5% | 20% | 20% | 20% | | | 20% | 0.5% |
| | | | | | | | | |
| **Transgene** | | | | | | | | |
| STAT3-ERT2 | | | | ✓ | | | ✓ | |
| | | | | | | | | |
| **Growth factors** | | | | | | | | |
| LIF | 10ng/ml | 10ng/ml | 10ng/ml | 10,000U/ml | 10ng/ml | 10ng/ml | 10,000U/ml | 10ng/ml |
| Activin A | 20ng/ml | | | | | | | 20ng/ml |
| bFGF | | 8ng/ml | 4ng/ml | | | | | |
| TGFb1 | | 1ng/ml | | | | | | |
| | | | | | | | | |
| **Small molecules** | | | | | | | | |
| BIRB796 | 2µM | | | | | | | |
| CGP77675 | 1µM | | | | | | | |
| CHIR99021 | 1.5µM | 3µM | 3µM | 3µM | | 1µM | | |
| PD0325901 | 1µM | 1µM | 0.5µM | 1µM | 1µM | | | 1µM |
| SP600125 | 5µM | 10µM | 10µM | | | | | |
| Gö6983 | 2µM | | | | 1µM | | | |
| SB202190 | | 5µM | | | | | | |
| SB203580 | | 10µM | 10µM | | | | | |
| (S)-(+)-Dimethindene maleate | | | | | | 2µM | | |
| Minocycline hydrochloride | | | | | | 2µM | | |
| IWR1 | 5µM | | | | | 0.75µM | | |
| XAV939 | | | | | 2µM | | | |
| CDK8/19i | | | | | | | 1.1µM | |
| IM-12 | | | | | | | | 1µM |
| SB590885 | | | | | | | | 0.5µM |
| WH-4-023 | | | | | | | | 1µM |

## List of antibodies

| Target | Supplier | Reference | Host species | Dilution for cells | Dilution for pre-implantation embryo | Dilution for cryosection | Dilution for immunoblotting |
|---|---|---|---|---|---|---|---|
| GFP | Thermo Fischer | A10262 | Chicken | 1:300 | 1:300 | 1:300 | NA |
| mCherry | Thermo Fischer | M11217 | Rat | 1:300 | 1:300 | NA | NA |
| OCT4 | Santa Cruz | sc-9081 | Rabbit | 1:300 | 1:300 | 1:300 | NA |
| NANOG | R&D Systems | AF1997 | Goat | 1:100 | 1:100 | 1:100 | NA |
| NANOG | R&D Systems | AF2729 | Goat | NA | 1:100 | 1:100 | NA |
| SOX2 | R&D Systems | AF2018 | Goat | 1:100 | 1:100 | 1:100 | NA |
| SOX17 | R&D Systems | AF1924 | Goat | 1:100 | 1:100 | NA | NA |
| GATA6 | R&D Systems | AF1700 | Goat | 1:100 | 1:100 | NA | NA |
| SUSD2-PE | BioLegend | 327406 | Mouse | 1:100 | NA | NA | NA |
| KLF4 | Tebu-Bio | 09-0021 | Mouse | 1:100 | NA | NA | NA |
| TFE3 | Thermo Fischer | PA5-21615 | Rabbit | 1:250 | NA | NA | NA |
| KLF17 | Sigma-Aldrich | HPA024629 | Rabbit | 1:100 | NA | NA | NA |
| HuN | Sigma-Aldrich | MAB1281 | Mouse | NA | 1:100 | NA | NA |
| TuJ1 | Sigma-Aldrich | T2200 | Rabbit | NA | NA | 1:100 | NA |
| H3K27me3 | Cell Signalling | 9733 | Rabbit | 1:1000 | NA | NA | 1/1000 |
| TFAP2C | R&D Systems | AF5059 | Goat | 1:100 | NA | NA | 1/1000 |
| TFCP2L1 | R&D Systems | AF5726 | Goat | 1:100 | NA | NA | 1/1000 |
| CDK2 | Cell Signalling | E8J9T | Rabbit | NA | NA | NA | 1/1000 |
| CDK4 | Abcam | Ab199728 | Rabbit | NA | NA | NA | 1/1000 |
| CYCLIN E1 | Cell Signalling | 20808 | Rabbit | NA | NA | NA | 1/1000 |
| CYCLIN A2 | Thermo Fischer | MA1-154 | Mouse | NA | NA | NA | 1/1000 |
| CYCLIN D1 | Cell Signalling | 55506 | Rabbit | NA | NA | NA | 1/1000 |
| CYCLIN D2 | Cell Signalling | 3741 | Rabbit | NA | NA | NA | 1/1000 |
| CYCLIN D3 | Cell Signalling | 2936 | Mouse | NA | NA | NA | 1/1000 |
| p27kip1 | Cell Signalling | 3688 | Rabbit | NA | NA | NA | 1/1000 |
| ACTINB | Sigma-Aldrich | A3854 | Mouse | NA | NA | NA | 1/10000 |

## References


CHEN, H., AKSOY, I., GONNOT, F., OSTEIL, P., AUBRY, M., HAMELA, C., ROGNARD, C., HOCHARD, A., VOISIN, S., FONTAINE, E., MURE, M., AFANASSIEFF, M., CLEROUX, E., GUIBERT, S., CHEN, J., VALLOT, C., ACLOQUE, H., GENTHON, C., DONNADIEU, C., DE VOS, J., SANLAVILLE, D., GUÉRIN, J.-F., WEBER, M., STANTON, L. W., ROUGEULLE, C., PAIN, B., BOURILLOT, P. & SAVATIER, P. 2015a. Reinforcement of STAT3 activity reprogrammes human embryonic stem cells to naïve-like pluripotency. *Nat Commun,* 6**,** 7095-7112.

CHEN, Y., NIU, Y., LI, Y., AI, Z., KANG, Y., SHI, H., XIANG, Z., YANG, Z., TAN, T., SI, W., LI, W., XIA, X., ZHOU, Q., JI, W. & LI, T. 2015b. Generation of Cynomolgus Monkey Chimeric Fetuses using Embryonic Stem Cells. *Cell Stem Cell,* 17**,** 116-124.

FANG, R., LIU, K., ZHAO, Y., LI, H., ZHU, D., DU, Y., XIANG, C., LI, X., LIU, H., MIAO, Z., ZHANG, X., SHI, Y., YANG, W., XU, J. & DENG, H. 2014. Generation of naive induced pluripotent stem cells from rhesus monkey fibroblasts. *Cell Stem Cell,* 15**,** 488-96.

GAFNI, O., WEINBERGER, L., MANSOUR, A. A., MANOR, Y. S., CHOMSKY, E., BEN-YOSEF, D., KALMA, Y., VIUKOV, S., MAZA, I., ZVIRAN, A., RAIS, Y., SHIPONY, Z., MUKAMEL, Z., KRUPALNIK, V., ZERBIB, M., GEULA, S., CASPI, I., SCHNEIR, D., SHWARTZ, T., GILAD, S., AMANN-ZALCENSTEIN, D., BENJAMIN, S., AMIT, I., TANAY, A., MASSARWA, R., NOVERSHTERN, N. & HANNA, J. H. 2013. Derivation of novel human ground state naive pluripotent stem cells. *Nature,* 504**,** 282-286.

GUO, G., VON MEYENN, F., ROSTOVSKAYA, M., CLARKE, J., DIETMANN, S., BAKER, D., SAHAKYAN, A., MYERS, S., BERTONE, P., REIK, W., PLATH, K. & SMITH, A. 2017. Epigenetic resetting of human pluripotency. *Development,* 144**,** 2748-2763.

LOVE, M. I., HUBER, W. & ANDERS, S. 2014. Moderated estimation of fold change and dispersion for RNA-seq data with DESeq2. *Genome Biol,* 15**,** 550.

NG, S. Y., JOHNSON, R. & STANTON, L. W. 2012. Human long non-coding RNAs promote pluripotency and neuronal differentiation by association with chromatin modifiers and transcription factors. *EMBO J,* 31**,** 522-33.

SAKAUE-SAWANO, A., YO, M., KOMATSU, N., HIRATSUKA, T., KOGURE, T., HOSHIDA, T., GOSHIMA, N., MATSUDA, M., MIYOSHI, H. & MIYAWAKI, A. 2017. Genetically Encoded Tools for Optical Dissection of the Mammalian Cell Cycle. *Mol Cell,* 68**,** 626-640 e5.

THEUNISSEN, T. W., POWELL, B. E., WANG, H., MITALIPOVA, M., FADDAH, D. A., REDDY, J., FAN, Z. P., MAETZEL, D., GANZ, K., SHI, L., LUNGJANGWA, T., IMSOONTHORNRUKSA, S., STELZER, Y., RANGARAJAN, S., D'ALESSIO, A., ZHANG, J., GAO, Q., DAWLATY, M. M., YOUNG, R. A., GRAY, N. S. & JAENISCH, R. 2014. Systematic Identification of Culture Conditions for Induction and Maintenance of Naive Human Pluripotency. *Cell Stem Cell,* 15**,** 471-487.

WIANNY, F., BERNAT, A., MARCY, G., HUISSOUD, C., MARKOSSIAN, S., LEVIEL, V., KENNEDY, H., SAVATIER, P. & DEHAY, C. 2008. Derivation and cloning of a novel Rhesus ES cell line stably expressing tau-GFP. *Stem Cells,* 26**,** 1444-1453.

YANG, J., RYAN, D. J., WANG, W., TSANG, J. C., LAN, G., MASAKI, H., GAO, X., ANTUNES, L., YU, Y., ZHU, Z., WANG, J., KOLODZIEJCZYK, A. A., CAMPOS, L. S., WANG, C., YANG, F., ZHONG, Z., FU, B., ECKERSLEY-MASLIN, M. A., WOODS, M., TANAKA, Y., CHEN, X., WILKINSON, A. C., BUSSELL, J., WHITE, J., RAMIREZ-SOLIS, R., REIK, W., GOTTGENS, B., TEICHMANN, S. A., TAM, P. P. L., NAKAUCHI, H., ZOU, X., LU, L. & LIU, P. 2017. Establishment of mouse expanded potential stem cells. *Nature,* 550**,** 393-397.
